# Pd/Xiang-Phos-catalyzed enantioselective intermolecular carboheterofunctionalization under mild conditions†
†Electronic supplementary information (ESI) available. CCDC 1981382 (**3ac**) and 1981384 (**3aa**). For ESI and crystallographic data in CIF or other electronic format see DOI: 10.1039/d0sc01391a


**DOI:** 10.1039/d0sc01391a

**Published:** 2020-05-29

**Authors:** Mengna Tao, Youshao Tu, Yu Liu, Haihong Wu, Lu Liu, Junliang Zhang

**Affiliations:** a Shanghai Key Laboratory of Green Chemistry and Chemical Processes , School of Chemistry and Molecular Engineering , East China Normal University , 3663 N. Zhongshan Road , Shanghai 200062 , P. R. China . Email: lliu@chem.ecnu.edu.cn; b College of Chemistry and Life Science , Advanced Institute of Materials Science , Changchun University of Technology , 2055 Yanan Street , Changchun , 130012 , P. R. China; c Department of Chemistry , Fudan University , 2005 Songhu Road , Shanghai 200438 , P. R. China . Email: junliangzhang@fudan.edu.cn; d State Key Laboratory of Organometallic Chemistry , Shanghai Institute of Organic Chemistry , CAS , 345 Lingling Road , Shanghai 200032 , P. R. China

## Abstract

A mild and practical Pd/**Xiang-Phos**-catalyzed enantioselective intermolecular carboheterofunctionalization reaction of 2,3-dihydrofurans is developed, leading to various optically active fused furoindolines and tetrahydrofurobenzofurans. The key to this transformation is employing two newly modified *N*-Me-Xiang-Phos ligands ((*S*, *R*_S_)-***N*-Me-X4**/**X5**) as chiral ligands under mild conditions. Moreover, this synthetic methodology can be efficiently applied to a variety of complex polysubstituted heterocycles with high chemo-, regio-, and enantio-selectivities *via* introducing diverse substituents on furan rings, which were hard to access by other routes.

## Abstract 2

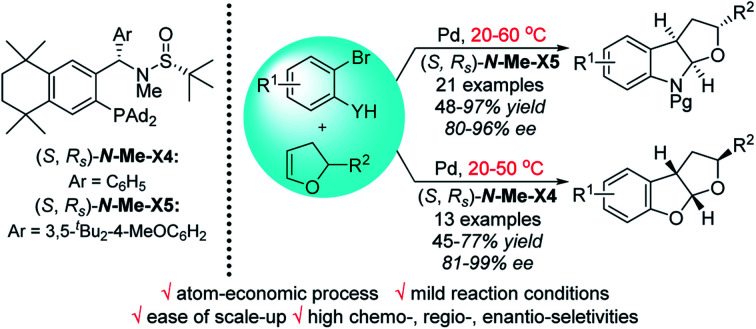
A highly chemo-, regio-, enantio-selectivitive Pd/**Xiang-Phos**-catalyzed intermolecular carboheterofunctionalization of 2,3-dihydrofurans for the synthesis of polysubstituted benzofused heterocycles mild conditions.

## Introduction

**Fig. 1:**
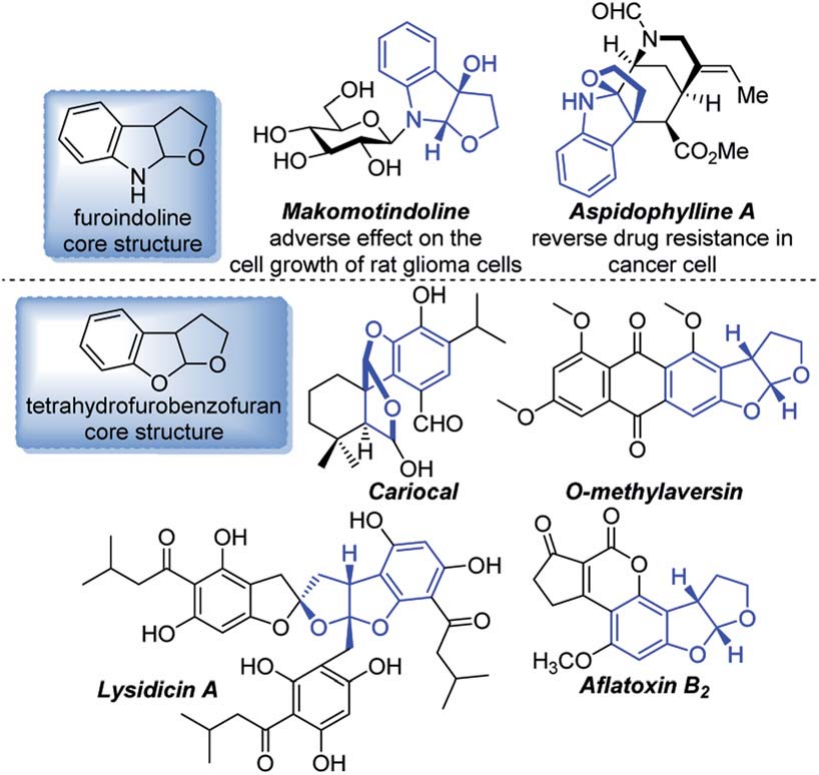
Representative examples of biologically active furoindolines and tetrahydrofurobenzofurans.

Benzofused heterocycles are ubiquitous moieties in natural products, pharmaceuticals, dyes and herbicides, in which furoindolines and tetrahydrofurobenzofurans are prevalent as key core structures (Fig. 1).^1^ These derivatives have shown significant anticancer, antimalarial and antimicrobial activities, as well as antioxidant properties, for instance, Makomotindoline and Aspidophylline A have shown a distinct effect on mammalian cells.^2^,^3^

The first enantioselective total synthesis of Aspidophylline A was described by Garg *via* a reductive interrupted Fischer indolization.^4^ You and co-workers developed a copper-catalyzed intermolecular dearomative cascade reaction of indoles, which also provided a powerful synthetic method for the construction of furoindolines.^5^ E. J. Corey achieved a short, asymmetric total synthesis of Aflatoxin B_2_*via* an aromative cascade reaction.^6^ Despite these seminal reports, hetero-annulation of alkenes developed by Catellani and Larock has become a classic and useful strategy for the construction of various heterocycles from readily available starting materials.^7^ Although various methods have been developed to construct these two skeletons, it still remains a considerable challenge to extend the substrate scope of asymmetric variants, particularly those that enable access to poly-substituted benzofused heterocycles.

**Scheme 1:**
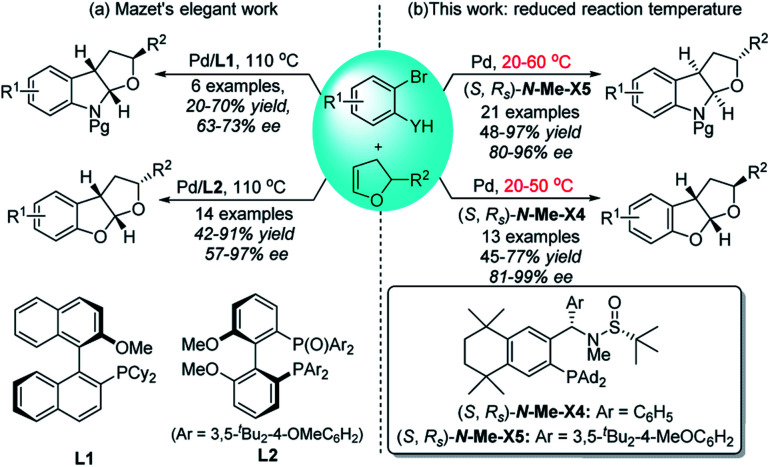
Pd-catalyzed intermolecular carbohetero-functionalization of 2,3-dihydrofurans.

Over the past two decades, palladium-catalyzed carbo-heterofunctionalization of alkenes has been proved to be a reliable and efficient method for the synthesis of a variety of poly-cyclic heterocycles.^8^ The majority of these reactions proceeded through a crucial hetero-palladation of alkenes with aryl halides along with N- or O-nucleophiles.^9^,^10^ However, the development of an enantioselective version, especially under mild conditions, poses a considerable challenge due to the lack of any suitable robust chiral catalyst. Recently, Mazet and co-workers reported the first asymmetric Pd-catalyzed *syn*-carboetherification and *syn*-carboamination of 2,3-dihydrofurans (2,3-dhfs) at 110 °C by utilizing two different chiral ligands (Scheme 1a).^11^ Inspired by the good performance of our chiral sulfinamide phosphine (**Sadphos**) ligands in the asymmetric construction of C–C and C–X bonds,^12^ we wondered whether **Sadphos** could realize the highly enantioselective carboetherification and carboamination of 2,3-dhfs under mild conditions and also address the low enantioselectivity issue of the carboamination reaction. Herein, we report a highly chemo-, regio-, and enantioselective palladium-catalyzed carbohetero-functionalization of 2,3-dhfs employing two newly modified **Xiang-Phos** ligands as chiral ligands, which can give direct access to enantioenriched poly-substituted functionalized furoindolines and tetrahydrofurobenzofurans in moderate to high yields with high enantio-selectivities at a reduced reaction temperature (Scheme 1b).

## Results and discussion

**Fig. 2:**
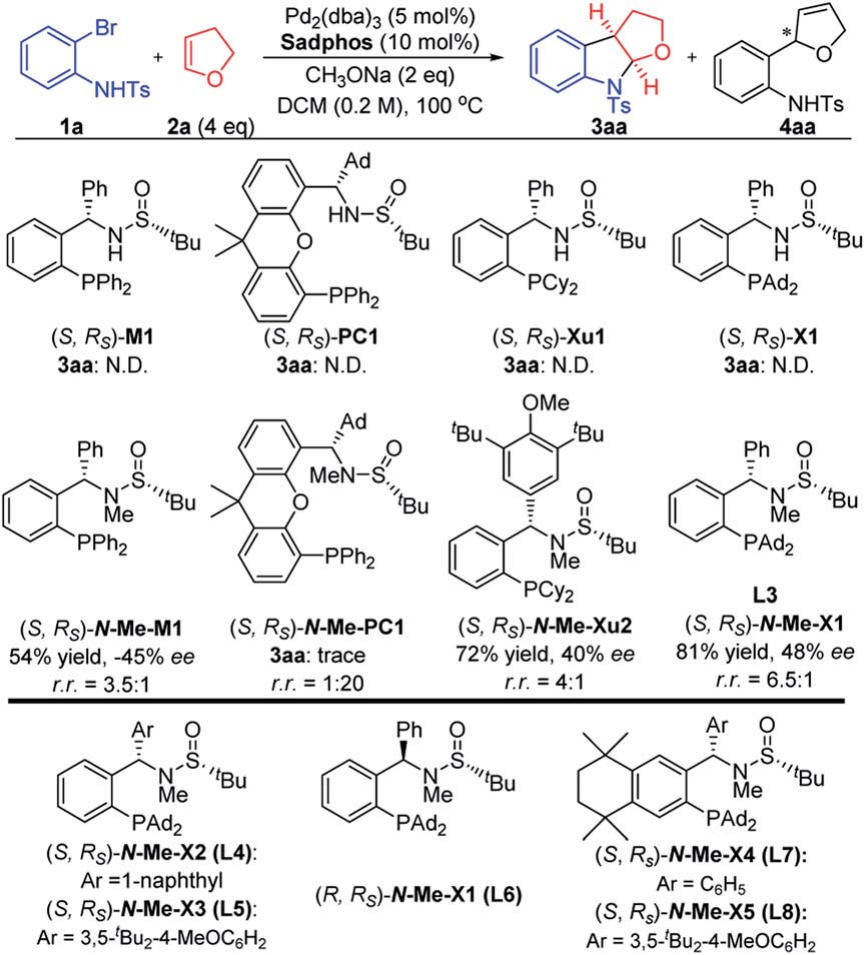
Screened **Sadphos** ligands.

With the use of our developed chiral sulfinamide phosphine ligands as the chiral ligands,^13^–^16^ the carboamination reaction of 2-bromoaniline derivative **1a** and 2,3-dhf **2a** was investigated. It was found that Ming-Phos **M1**, PC-Phos **PC1**, Xu-Phos **Xu1** and Xiang-Phos **X1** did not efficiently deliver the desired product. As observed in our previous work, the N–H bond in ligands could greatly affect the reactivity as well as enantioselectivity in some cases.^16^ Several representative *N*-Me sulfinamide phosphine ligands lacking the hydrogen-bonding site were further investigated. We were pleased to find that the desired product **3aa** could be obtained in 81% yield with a 48% ee value in the presence of (*S*, *R*_S_)-***N*-Me-X1** and CH_3_ONa, albeit with a small amount of the Heck byproduct **4aa**. Other chiral ligands such as Ming-Phos ***N*-Me-M1** and Xu-Phos ***N*-Me-Xu2** showed less efficiency comparatively, leading to a lower yield and enantioselectivity along with a poor regioselectivity (Fig. 2).

Under the conditions of **Xiang-Phos** (*S*, *R*_S_)-***N*-Me-X1** utilized as the chiral ligand, NaOPh appeared to be the optimal base, affording **3aa** in 78% ee albeit with a 2 : 1 regioselectivity ratio (r.r.) (Table 1, entries 1–5). Solvent screening showed that 1,2-DCE gave a better yield (81%) and r.r. (9 : 1) with 87% ee (Table 1, entries 6–9). The result obtained employing other chiral ***N*-Me-Xiang-Phos** ligands indicated that the introduction of steric hindrance on the phenyl backbone and enhancement of the electron-donating character were beneficial for the catalytic enantio-selectivity and regioselectivity (Table 1, entries 10–14). Employing the newly modified ***N*-Me-X5** as the chiral ligand, a series of Pd precursors were then screened, showing that a five-membered cyclic palladium precatalyst was competent for the carboamination cyclization (Table 1, entries 15–19). Comparably better outcomes were obtained under mild conditions by lowering the temperature to 20 °C (Table 1, entries 20–23). Inspired by previous findings that the addition of a trace amount of water may help to increase the reactivity and the stereoselectivity,^17^ the water effect was studied, and indeed, we found that the addition of 2 equivalents of water to the system led to a significantly improved reactivity and reproducible enantioselectivity. In terms of the reactivity and enantio- and regio-selectivity, the reaction conditions illustrated in entry 22 were utilized in the following substrate scope investigations (please see Table S1 in the ESI for details†).

**Table 1:** Optimization of the carboamination conditions^*a*^

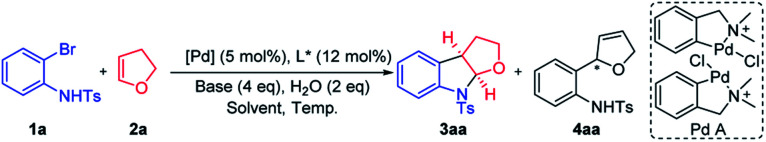					
Entry	[Pd]/**L**	Base	Solvent/*T* (°C)	**3**/**4**	Yield^*b*^ ^,^^*c*^ (ee) [%]
1	Pd_2_(dba)_3_/**L3**	NaO^*t*^Bu	DCM/100	5 : 1	73(47)
2	Pd_2_(dba)_3_/**L3**	LiO^*t*^Bu	DCM/100	—	Trace
3	Pd_2_(dba)_3_/**L3**	KO^*t*^Bu	DCM/100	—	Mix
4	Pd_2_(dba)_3_/**L3**	NaOEt	DCM/100	2 : 1	52(40)
5	Pd_2_(dba)_3_/**L3**	NaOPh	DCM/100	2 : 1	63(78)
6	Pd_2_(dba)_3_/**L3**	NaOPh	MTBE/100	1 : 1	44(60)
7	Pd_2_(dba)_3_/**L3**	NaOPh	1,2-DCE/100	9 : 1	81(76)
8	Pd_2_(dba)_3_/**L3**	NaOPh	Toluene/100	1 : 1	42(53)
9	Pd_2_(dba)_3_/**L3**	NaOPh	MeOH/100	1 : 1	39(59)
10	Pd_2_(dba)_3_/**L4**	NaOPh	1,2-DCE/100	9 : 1	78(87)
11	Pd_2_(dba)_3_/**L5**	NaOPh	1,2-DCE/100	>30 : 1	81(93)
12	Pd_2_(dba)_3_/**L6**	NaOPh	1,2-DCE/100	—	Trace
13	Pd_2_(dba)_3_/**L7**	NaOPh	1,2-DCE/100	15 : 1	77(77)
14	Pd_2_(dba)_3_/**L8**	NaOPh	1,2-DCE/100	>30 : 1	83(93)
15	Pd(dba)_2_/**L8**	NaOPh	1,2-DCE/100	>30 : 1	79(94)
16	Pd_2_(dba)_3_·CHCl_3_/**L8**	NaOPh	1,2-DCE/100	>30 : 1	81(94)
17	Pd(OAc)_2_/**L8**	NaOPh	1,2-DCE/100	>30 : 1	74(94)
18	(η^3^-C_3_H_5_)_2_Pd_2_Cl_2_/**L8**	NaOPh	1,2-DCE/100	>30 : 1	69(94)
19	Pd A/**L8**	NaOPh	1,2-DCE/100	>30 : 1	82(94)
20	Pd A/**L8**	NaOPh	1,2-DCE/80	>30 : 1	81(93)
21	Pd A/**L8**	NaOPh	1,2-DCE/50	>30 : 1	81(95)
22	Pd A/**L8**	NaOPh	1,2-DCE/20	>30 : 1	84(96)
23^*d*^	Pd A/**L8**	NaOPh	1,2-DCE/20	>30 : 1	79(96)

^*a*^Unless otherwise specified, all reactions were carried out with **1a** (0.2 mmol), **2a** (0.8 mmol, 4 eq.), a [Pd] source (0.01 mmol, 5 mol%), ***N*-Me-Xiang-phos** (0.024 mmol, 12 mol%), base (0.8 mmol, 4 eq.), and H_2_O (7.2 μL, 2 eq.) in a solvent (1 mL, 0.2 M).

^*b*^Yield of isolated product **3aa**.

^*c*^Determined by chiral HPLC.

^*d*^2 eq. H_2_O were removed.

**Scheme 2:**
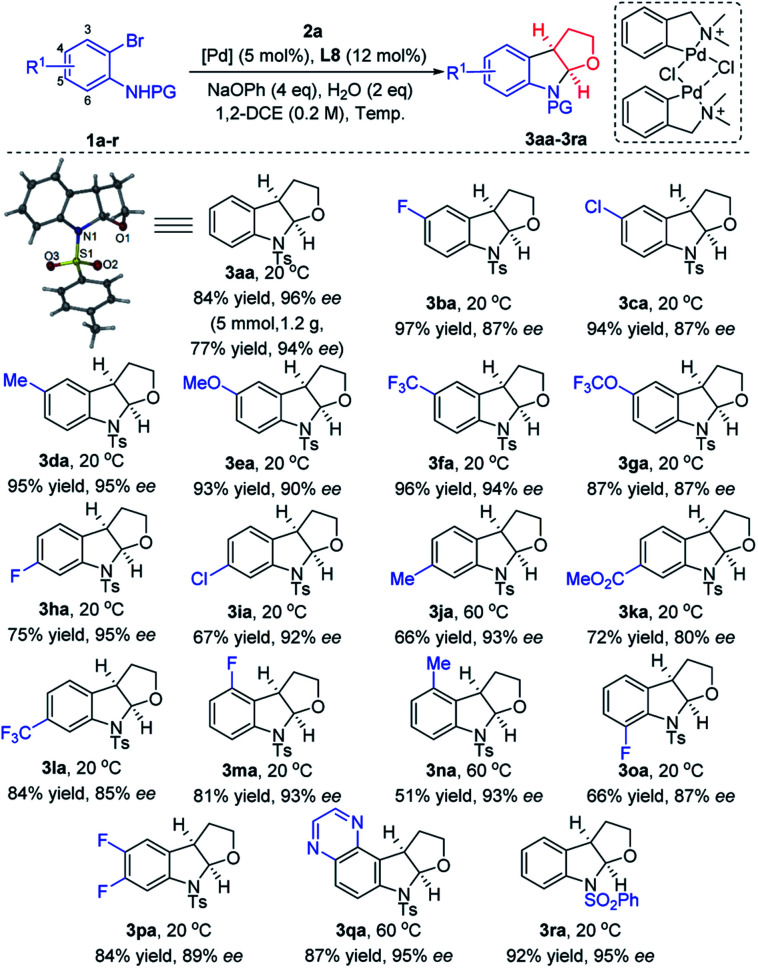
Scope of carboamination of 2,3-dhf with 2-bromoanilines.

Various substituted *N*-(2-bromophenyl)-*p*-tolylsulfonamide derivatives **1a–r** were subsequently employed as coupling partners in the enantioselective intermolecular carboamination of 2,3-dhf (Scheme 2). Remarkably, a wide range of 2-Br-anilines bearing electronically diverse substituents at C4 and C5 such as halogens, –Me, –OMe, –CF_3_, –OCF_3_, and –CO_2_Me reacted smoothly and furnished the corresponding furoindolines **3aa–3la** in good yields (up to 97%) and ee's (up to 96%). Further substrate scope investigations demonstrated that the electronic properties of substituents at C3 and C6 did affect the yields and enantioselectivities, and produced products **3na** and **3oa** in lower yields comparatively. Notably, the disubstituents on phenyl rings were also applicable in this cyclization reaction, affording **3pa** in 84% yield with 89% ee, as well as **3qa** containing a heterocycle in 87% yield and 95% ee. When *N*-(2-bromophenyl)-benzenesulfonamide was explored as an alternative to **1a**, to our delight, the furoindoline product **3ra** was formed in a nearly quantitative yield (92%) with high enantioselectivity (95%). We also replaced the protective groups on the nitrogen atom with Ms and Ns, but only trace products could be detected by NMR. Other nitrogen protecting groups on aniline, such as Boc, Cbz and Bz, were not tolerated, and in these cases the desired product was not observed. To our delight, a gram-scale reaction was conducted to further demonstrate the potential synthetic utility of this methodology, delivering 1.2 g of **3aa** in 77% yield and 94% ee with 2.5 mol% palladium catalyst at 20 °C for 6 days. The absolute configuration of this series of products was confirmed by the X-ray diffraction analysis of **3aa**.^18^

**Scheme 3:**
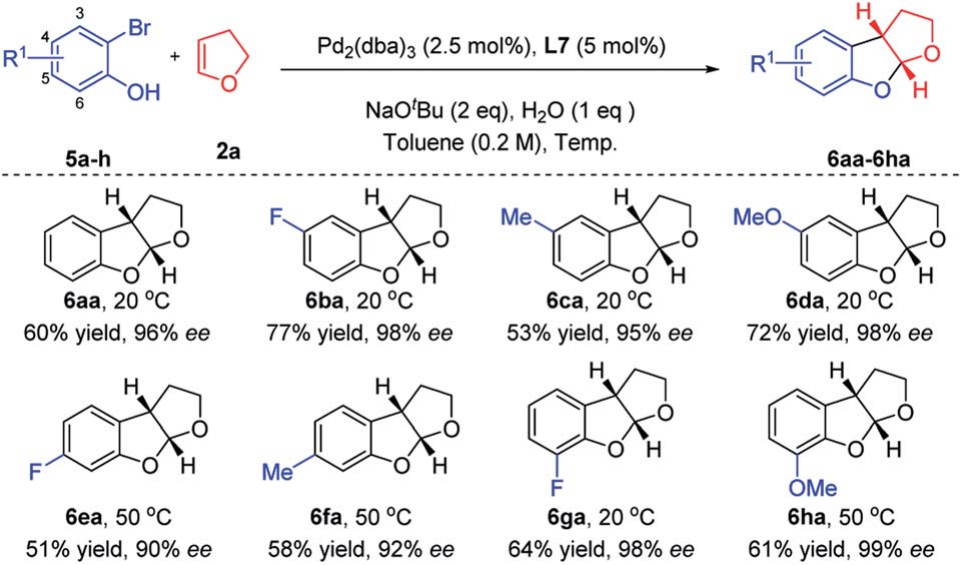
Scope of carboetherification of 2,3-dhf with 2-bromophenols.

After a quick survey of the construction of tetrahydrofuro-benzofuran (Table 2, please see Table S2 in the ESI for details†), the optimal reaction conditions were identified (Table 2, entry 7). A series of substituted 2-bromophenol derivatives **5a–h** were subsequently employed as coupling partners in the enantio-selective intermolecular carboetherification of 2,3-dhf with the use of ***N*-Me-X4** under mild conditions (Scheme 3). Of note, an exciting enantioselective cyclization was realized when substrates containing diverse substituents at C4, C5 and C6 with different electronic properties (–F, –Me, and –OMe) participated smoothly, delivering products in 90% to 99% ee's. It is a pity that only a trace amount of product was observed when substituents were present at C3 of the phenyl ring.

**Table 2:** Selective optimization of the carboetherification conditions^*a*^

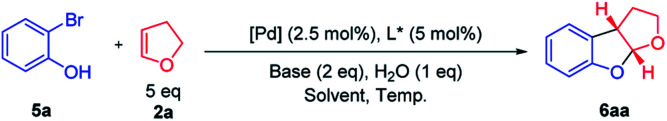					
Entry	[Pd]/**L**	Base	Solvent	*T* (°C)	Yield^*b*^ ^,^^*c*^ (ee) [%]
1	Pd_2_(dba)_3_/**L3**	NaO^*t*^Bu	Toluene	80	40(87)
2	Pd_2_(dba)_3_/**L3**	NaOPh	Toluene	80	30(38)
3^*d*^	Pd_2_(dba)_3_/**L3**	NaO^*t*^Bu	DCM	80	45(33)
4	Pd_2_(dba)_3_/**L3**	NaO^*t*^Bu	Toluene	20	55(95)
5	Pd A/**L3**	NaO^*t*^Bu	Toluene	20	51(95)
6	Pd_2_(dba)_3_/**L5**	NaO^*t*^Bu	Toluene	20	44(85)
7	Pd_2_(dba)_3_/**L7**	NaO^*t*^Bu	Toluene	20	60(96)
8	Pd_2_(dba)_3_/**L8**	NaO^*t*^Bu	Toluene	20	52(82)

^*a*^Unless otherwise specified, all reactions were carried out with **5a** (0.2 mmol), **2a** (1 mmol, 5 eq.), a [Pd] source (0.005 mmol, 2.5 mol%), ***N*-Me-Xiang-Phos** (0.01 mmol, 5 mol%), base (0.4 mmol, 2 eq.), and H_2_O (3.6 μL, 1 eq.) in a solvent (1 mL, 0.2 M).

^*b*^Yield of isolated product.

^*c*^Determined by chiral HPLC.

^*d*^Pd_2_(dba)_3_ was added to 5 mol%, and **L3** was added to 10 mol%.

**Scheme 4:**
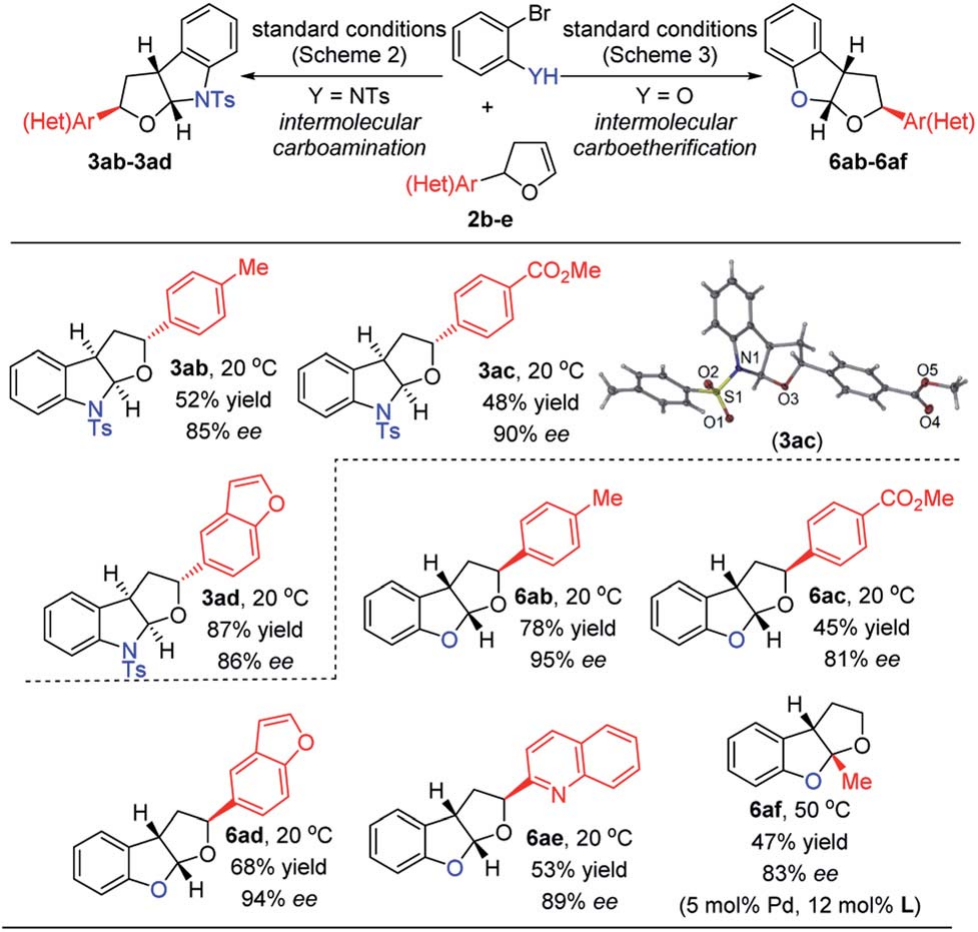
Asymmetric carboheterofunctionalization of substituted 2,3-dhfs.

To delve into the construction of poly-substituted fused furo-indolines and tetrahydrofurobenzofurans, a variety of dihydrofuran derivatives **2b–2e**, which could be readily prepared by a classic Heck reaction, were subjected to carbofunctionalization. In these two cases, variation of the electronic parameters of the phenyl groups on dhf rings had a slight influence on the yields and enantiocontrol of the carbohetero-functionalizations (Scheme 4, **3ab**/**6ab**, **3ac**/**6ac**). Considering the O- and N-containing heterocycle substituents on dhf rings, the phenyl groups could be swapped for the benzofuran and quinoline substituents (**3ad**/**6ad**, **6ae**), still maintaining the efficiency of the transformations. 5-Methyl-2,3-dhf was next examined to investigate the formation of an all-carbon quaternary stereocenter. The carboetherification reaction took place smoothly when increasing the loading of the palladium precatalyst and chiral ligand at a higher temperature (**6af**). However, only the corresponding debromination product was detected in the carboamination reaction system. The absolute configuration of the poly-substituted carboamination products was confirmed by X-ray diffraction analysis of **3ac**,^18^ while the absolute configuration of poly-substituted carbo-etherification products was assigned by comparing the rotational value and ^1^H,^1^H-NOESY-NMR spectrum (please see the ESI for details†) of **6ab** between our work and Mazet's work.^11^ The substituted aromatic ring on the tetrahydrofuran ring was in the (*S*)-configuration, and is a diastereomer of the corresponding product in Mazet's work.

**Scheme 5:**
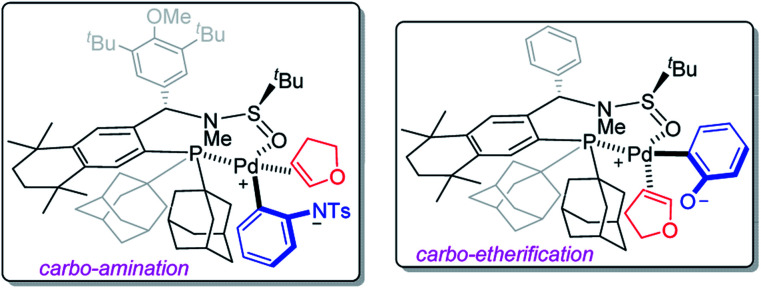
Chirality-induction models.

Based on Mazet's studies, as well as our observations on Pd/**Sadphos** catalytic systems, the chirality-induction models of carbo-amination and -etherification were proposed according to the absolute configuration of products **3aa** and **6aa**, as shown in Scheme 5. We supposed that the reaction was initiated by a classic oxidative addition, which would be followed by ligand exchange, deprotonation and coordination of 2,3-dhf. The key step of asymmetric hetero-palladation was hypothesized to occur to ultimately construct optically active benzofused heterocycles with high regio- and enantio-selectivities.

## Conclusions

In summary, we have demonstrated an efficient Pd-catalyzed enantioselective intermolecular carboheterofunctionalization of 2,3-dihydrofurans for the synthesis of poly-substituted benzofused heterocycles. The new ***N*-Me-Xiang-Phos X4/X5** ligands are responsible for the high reactivity and enantioselectivity. This strategy could be conducted under mild conditions and easily extended to a wide range of chiral fused furoindolines and tetrahydrofurobenzofurans with high chemo-, regio-, and enantio-selectivities, which made the method extremely attractive. In addition, a gram-scale reaction of the representative product **3aa** was investigated to further demonstrate the potential synthetic applications of this method. Further applications of **Sadphos** in other transition-metal-catalyzed reactions are underway in our group and will be reported in due course.

## Conflicts of interest

There are no conflicts to declare.

## Supplementary Material

Supplementary information

Crystal structure data
