# Supplementary material for: Pd/Xiang-Phos-catalyzed enantioselective intermolecular carboheterofunctionalization under mild conditions
Source: Chem Sci. 2020 May 29;11(24):6283–8. doi: 10.1039/d0sc01391a (PMC7473404; doi:10.1039/d0sc01391a)

Electronic Supplementary Material (ESI) for Chemical Science.
This journal is © The Royal Society of Chemistry 2020

Supporting Information

Pd/Xiang-Phos-Catalyzed Enantioselective Intermolecular Carboheterofunctionalizations Under Mild Conditions

Mengna Tao[a], Youshao Tu[b], Yu Liu[b], Haihong Wu[a], Lu Liu*, [a], and Junliang Zhang*, [c],[d]

[a] Shanghai Key Laboratory of Green Chemistry and Chemical Processes, School of Chemistry and Molecular Engineering, East China Normal University, 3663 N. Zhongshan Road, Shanghai 200062, P. R. China.

[b] College of Chemistry and Life Science, Advanced Institute of Materials Science, Changchun University of Technology, Changchun, 130012, P. R. China.

[c] Department of Chemistry, Fudan University, 2005 Songhu Road, Shanghai 200438, P. R. China
[d] State Key Laboratory of Organometallic Chemistry, Shanghai Institute of Organic Chemistry, Chinese Academy of Science.

E-mail: lliu@chem.ecnu.edu.cn
junliangzhang@fudan.edu.cn

Table of Contents

1 General Information……………………………………………….........................… S3

2 Optimization of the intermolecular carboheterofunctionalizations………....…S4

2.1 Optimization of the intermolecular carboamination of 2,3-dihydrofuran…...……....S4

2.2 Optimization of the intermolecular carboetherification of 2,3-dihydrofuran…….....S6

3 Experimental procedures…………………………………………….....…..…..S8

3.1 General procedure for the synthesis of (*S, R$_S$*)-***N*-Me-X4/X5**…………………....…S8

3.2 General procedure for the intermolecular carboamination of 2,3-dihydrofuran using 2-bromoaniline derivatives (GP1)…………………………………………....………..S9

3.3 General procedure for the intermolecular carboetherification of 2,3-dihydrofuran using 2-bromophenol derivatives (GP2)………………………….……………....……...…S9

3.4 General procedure for the synthesis of 2-substituted-2,3-dihydrofurans………..…S10

4 General Data for (*S, R$_S$*)-*N*-Me-X4/X5, 3 and 6………………………….………..…...S11

5 Absolute Configuration of 3 and 6……..S37

6 References………………………………...……………………….……..…..….S39

7 ^{1}H, ^{13}C, ^{19}F, ^{31}P Spectra for (*S, R$_S$*)-*N*-Me-X4/X5, 3 and 6………..……..……....S40

1. General Information

Unless otherwise noted, all reactions were carried out under a nitrogen atmosphere; materials obtained from commercial suppliers were used directly without further purification. The $[\alpha]_D$ was recorded using PolAAr 3005 High Accuracy Polarimeter. ^{1}H NMR spectra and ^{13}C NMR spectra were recorded on a Bruker 400 MHz or 500 MHz spectrometer in chloroform-d_3, and were calibrated with $CDCl_3$ (δ = 77.00 ppm). ^{19}F NMR spectra were recorded on a Bruker 400 MHz spectrometer in chloroform-d_3. Chemical shifts (in ppm) were referenced to tetramethylsilane (δ = 0 ppm) in $CDCl_3$ as an internal standard. The data is being reported as (s = singlet, d = doublet, dd = doublet of doublet, t = triplet, m = multiplet or unresolved, br = broad signal, coupling constant(s) in Hz, integration).

Trichloromethane ($CHCl_3$)， dichloromethane, dichloroethane and acetonitrile were freshly distilled from CaH_2; tetrahydrofuran (THF), toluene and ether were dried with sodium benzophenone and distilled before use.

Reactions were monitored by thin layer chromatography (TLC) using silicycle pre-coated silica gel plates. Flash column chromatography was performed on silica gel 60 (particle size 200-400 mesh ASTM, purchased from Yantai, China) and eluted with petroleum ether/ethyl acetate. All reagents and solvents were used as received from commercial sources (*Energy Chemical, J&K®, Adamas-beta®, Bidepharm*) without further purification. The substrates **2b-f** were synthesized according to published procedures[1]. The spectral data of the substrates were consisted with that reported in the literature[2]. The enantionmeric excesses of the products were determined by chiral stationary phase HPLC using a Chiralpak IA, IB, IC , IF, ADH, ODH, OJH, OJ3.

2. Optimization of the intermolecular carboheterofunctionalizations

2.1 Table S1. Detailed optimization of the enantioselective intermolecular carboamination of 2,3-dihydrofuran and **1a**[a]

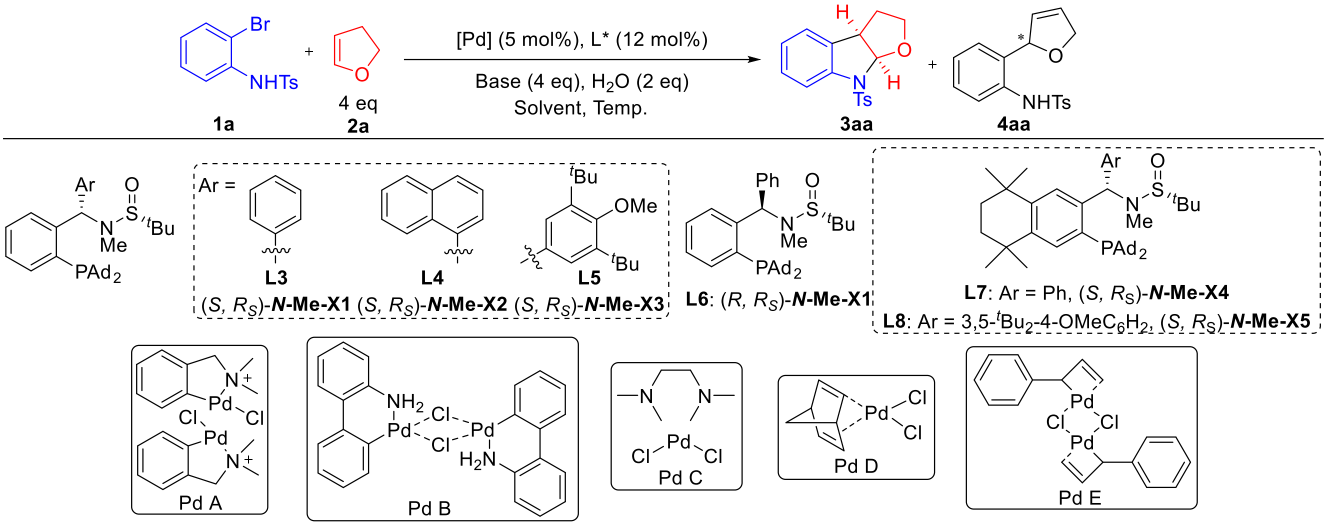

Entry	Pd	L*	Base	Solvent	Temp. (°C)	Yield (Ee) (%)[b,c]	r.r.[d]
1	$Pd_2(dba)_3$	**L3**	CH_3ONa	DCM	100	81(48)	13:2
2	$Pd_2(dba)_3$	**L3**	NaO^{t}Bu	DCM	100	73(47.3)	5:1
3	$Pd_2(dba)_3$	**L3**	LiO^{t}Bu	DCM	100	trace	-
4	$Pd_2(dba)_3$	**L3**	KO^{t}Bu	DCM	100	mix	-
5	$Pd_2(dba)_3$	**L3**	NaOEt	DCM	100	52(40.3)	2:1
6	$Pd_2(dba)_3$	**L3**	NaOPh	DCM	100	63(77.5)	2:1
7	$Pd_2(dba)_3$	**L3**	Cs_2CO_3	DCM	100	mix	-
8	$Pd_2(dba)_3$	**L3**	NaOPh	MTBE	100	44(60)	1:1
9	$Pd_2(dba)_3$	**L3**	NaOPh	THF	100	41(23)	1:2
10	$Pd_2(dba)_3$	**L3**	NaOPh	1,2-DCE	100	81(76)	9:1
11	$Pd_2(dba)_3$	**L3**	NaOPh	$CHCl_3$	100	mix	-
12	$Pd_2(dba)_3$	**L3**	NaOPh	Toluene	100	42(53)	1:1
13	$Pd_2(dba)_3$	**L3**	NaOPh	MeOH	100	39(59)	1:1
14	$Pd_2(dba)_3$	**L3**	NaOPh	MeCN	100	mix	-
15	$Pd_2(dba)_3$	**L3**	NaOPh	DMF	100	69(0)	6:1
16	$Pd_2(dba)_3$	**L4**	NaOPh	1,2-DCE	100	78(87)	9:1
17	$Pd_2(dba)_3$	**L5**	NaOPh	1,2-DCE	100	81(93.1)	>30:1
18	$Pd_2(dba)_3$	**L6**	NaOPh	1,2-DCE	100	trace	-
19	$Pd_2(dba)_3$	**L7**	NaOPh	1,2-DCE	100	77(77)	15:1
20	$Pd_2(dba)_3$	**L8**	NaOPh	1,2-DCE	100	83(93)	>30:1
21	$Pd(dba)_2$	**L8**	NaOPh	1,2-DCE	100	79(93.7)	>30:1

22	$Pd_2(dba)_3$•$CHCl_3$	**L8**	NaOPh	1,2-DCE	100	81(93.9)	>30:1
23	$Pd(OAc)_2$	**L8**	NaOPh	1,2-DCE	100	74(94.1)	>30:1
24	$(\eta^3\text{-}C_3H_5)_2Pd_2Cl_2$	**L8**	NaOPh	1,2-DCE	100	69(93.7)	>30:1
25	Pd A	**L8**	NaOPh	1,2-DCE	100	82(94.1)	>30:1
26	Pd B	**L8**	NaOPh	1,2-DCE	100	74(86.9)	>30:1
27	Pd C	**L8**	NaOPh	1,2-DCE	100	N.D.	-
28	Pd D	**L8**	NaOPh	1,2-DCE	100	77(81.3)	>30:1
29	Pd E	**L8**	NaOPh	1,2-DCE	100	trace	-
30	Pd A	**L8**	NaOPh	1,2-DCE	80	81(93.1)	>30:1
31	Pd A	**L8**	NaOPh	1,2-DCE	50	81(95.3)	>30:1
32	Pd A	**L8**	NaOPh	1,2-DCE	20	84(95.5)	>30:1
34[e]	Pd A	**L8**	NaOPh	1,2-DCE	20	73(91.6)	>30:1
35[f]	Pd A	**L8**	NaOPh	1,2-DCE	20	77(93.8)	>30:1
35[g]	Pd A	**L8**	NaOPh	1,2-DCE	20	81(95.3)	>30:1
35[h]	Pd A	**L8**	NaOPh	1,2-DCE	20	79(94.9)	>30:1
33[i]	Pd A	**L8**	NaOPh	1,2-DCE	20	79(95.7)	>30:1

[a] Unless otherwise specified, all reactions were carried out with **1a** (0.2 mmol), **2a** (0.8 mmol, 4 eq), [Pd] source (0.01 mmol, 5 mol%), ***N*-Me-Xiang-Phos** (0.024 mmol, 12 mol%), Base (0.8 mmol, 4 eq), H_2O (7.2 μL, 2 eq) in solvent (1 mL, 0.2 M). [b] Yield of isolated product. [c] Determined by chiral HPLC. [d] Reaction *r.r.*s of **3aa**:**4aa**, determined by chiral HPLC. [e] 2.5 mol% Pd A, 6 mol% **L8** were employed. [f] 2 eq NaOPh and 1 eq H_2O were employed. [g] 1 eq H_2O were employed. [h] 50 mol% H_2O were employed. [i] 2 eq H_2O was removed.

2.2 Table S2. Detailed optimization of the enantioselective intermolecular carbo-etherification of 2,3-dihydrofuran and **5a**[a]

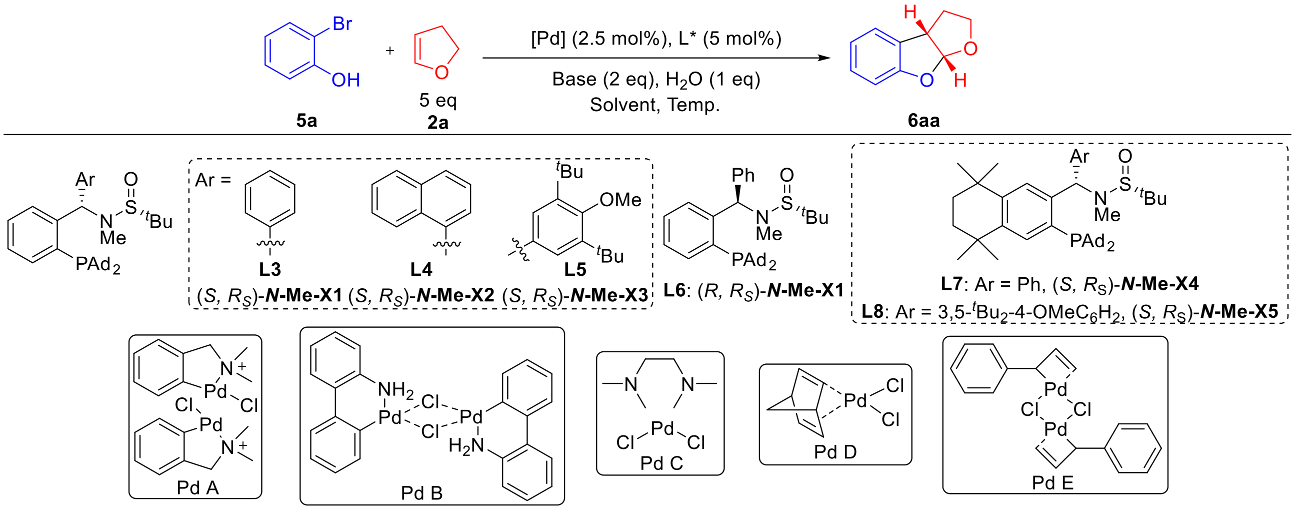

Entry	Pd	L*	Base	Solvent	Temp. (°C)	Yield (Ee) (%)[b,c]
1	$Pd_2(dba)_3$	**L3**	NaO*t*Bu	Toluene	80	40(87.1)
2	$Pd_2(dba)_3$	**L3**	NaOPh	Toluene	80	30(37.9)
3	$Pd_2(dba)_3$	**L3**	CH_3ONa	Toluene	80	trace
4	$Pd_2(dba)_3$	**L3**	CH_3OLi	Toluene	80	trace
5	$Pd_2(dba)_3$	**L3**	LiO*t*Bu	Toluene	80	trace
6	$Pd_2(dba)_3$	**L3**	KO*t*Bu	Toluene	80	mix
7	$Pd_2(dba)_3$	**L3**	Cs_2CO_3	Toluene	80	mix
8[d]	$Pd_2(dba)_3$	**L3**	NaO*t*Bu	THF	80	30(74.5)
9[d]	$Pd_2(dba)_3$	**L3**	NaO*t*Bu	MTBE	80	34(67.1)
10[d]	$Pd_2(dba)_3$	**L3**	NaO*t*Bu	DCM	80	45(32.5)
11[d]	$Pd_2(dba)_3$	**L3**	NaO*t*Bu	1,2-DCE	80	39(20.3)
12[d]	$Pd_2(dba)_3$	**L3**	NaO*t*Bu	Toluene	80	40(71.5)
13	$Pd_2(dba)_3$	**L3**	NaO*t*Bu	Toluene	20	55(95.3)
14	$Pd_2(dba)_3$	**L3**	NaO*t*Bu	THF	20	30(97)
15	$Pd(dba)_2$	**L3**	NaO*t*Bu	Toluene	20	23(94.3)
16	$Pd_2(dba)_3•CHCl_3$	**L3**	NaO*t*Bu	Toluene	20	38(96.5)
17	$Pd(OAc)_2$	**L3**	NaO*t*Bu	Toluene	20	49(91.5)
18	$(\eta^3\text{-}C_3H_5)_2Pd_2Cl_2$	**L3**	NaO*t*Bu	Toluene	20	53(94.3)
19	Pd A	**L3**	NaO*t*Bu	Toluene	20	51(94.7)
20	Pd B	**L3**	NaO*t*Bu	Toluene	20	42(83.1)
21	Pd C	**L3**	NaO*t*Bu	Toluene	20	trace
22	Pd D	**L3**	NaO*t*Bu	Toluene	20	33(77.2)
23	Pd E	**L3**	NaO*t*Bu	Toluene	20	mix
24	$Pd_2(dba)_3$	**L4**	NaO*t*Bu	Toluene	20	49(94.3)

25	$Pd_2(dba)_3$	**L5**	NaO*t*Bu	Toluene	20	44(85)
26	$Pd_2(dba)_3$	**L6**	NaO*t*Bu	Toluene	20	trace
27	$Pd_2(dba)_3$	**L7**	NaO*t*Bu	Toluene	20	60(96.3)
28	$Pd_2(dba)_3$	**L8**	NaO*t*Bu	Toluene	20	52(81.9)
29[e]	$Pd_2(dba)_3$	**L7**	NaO*t*Bu	Toluene	20	21(91.1)
30[f]	$Pd_2(dba)_3$	**L7**	NaO*t*Bu	Toluene	20	35(94.5)

[a] Unless otherwise specified, all reactions were carried out with **5a** (0.2 mmol), **2a** (1 mmol, 5 eq), [Pd] source (0.005 mmol, 2.5 mol%), ***N*-Me-Xiang-Phos** (0.01 mmol, 5 mol%), Base (0.4 mmol, 2 eq), H_2O (3.6 µL, 1 eq) in solvent (1 mL, 0.2 M). [b] Yield of isolated product. [c] Determined by chiral HPLC. [d] $Pd_2(dba)_3$ was added to 5 mol%, also **L3** was added to 10 mol%. [e] 1 eq H_2O was removed. [f] 4 eq NaO*t*Bu and 1 eq H_2O were employed.

3. Experimental procedures

3.1 General procedure for the synthesis of (*S*, R_S)-***N*-Me-X4/X5**.

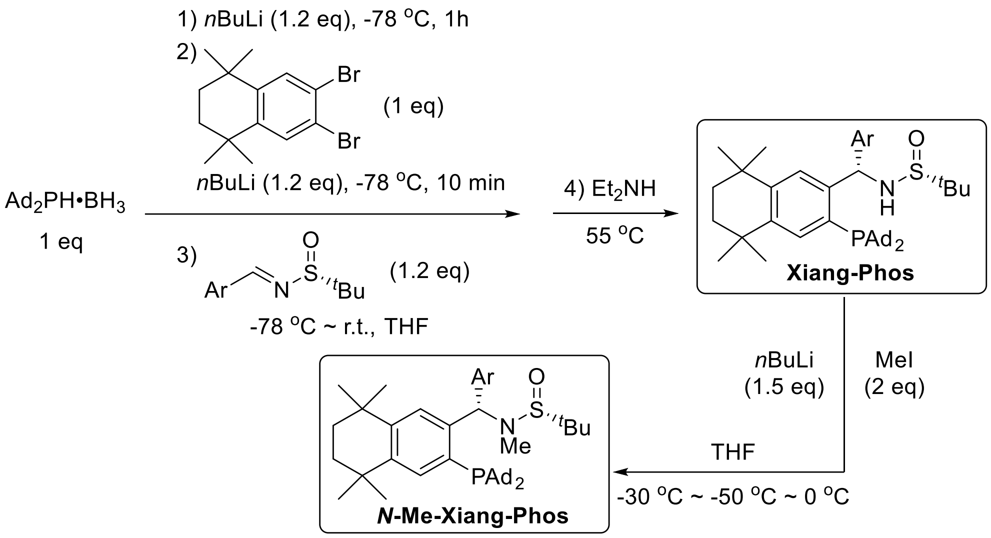

To a solution of di-1-adamantylphosphine borane (5 mmol) in dry THF (25 mL) was added ^{n}BuLi (1.2 eq, 1.6 M in hexane) dropwise under argon at -78 ℃. The resulting solution at this temperature during 1 hour and 1,2-dibromo compound (5 mmol) was added dropwise followed by ^{n}BuLi (1.2 eq, 1.6 M in hexane). After 10 minutes at -78 ℃, (R_S)-sulfinyl imine (6 mmol) was added and the reaction mixture was warmed to room temperature overnight. The reaction mixture was quenched by the addition of NH_4Cl (aq.) and diluted with EtOAc. The organic layer was separated, and the aqueous layer was extracted twice with EtOAc. The combined organic layers were dried over Na_2SO_4, filtered, concentrated. The crude product was dealed with Et_2NH (15 mL) and the resulting solution was stirred under argon at 55 ℃. After the reaction was complete (monitored by TLC), solvent was removed under reduced pressure. The crude product was then purified by flash column chromatography on silica gel (Petroleum ether : EtOAc = 10:1) to afford the desired **Xiang-Phos**.

To a solution of **Xiang-Phos** (2 mmol) in dry THF (5 mL) was added ^{n}BuLi (1.5 eq, 1.6 M in hexane) dropwise under argon at -30 ℃. The resulting solution was stilled at this temperature for 1 hour and then MeI (2 eq) was added dropwise at -50 ℃. The resulting solution was stilled at this temperature for 1.5 hours and then stilled at 0 ℃ for another 1.5 hours. The reaction mixture was quenched by the addition of NH_4Cl (aq.) and diluted with EtOAc. The organic layer was separated, and the aqueous layer was extracted twice with EtOAc. The combined organic layers were dried over Na_2SO_4, filtered, concentrated. The crude product was then purified by flash column chromatography on silica gel (Petroleum ether: EtOAc = 10:1) to afford the desired ***N*-Me-Xiang-Phos**.

3.2 General procedure for the intermolecular carboamination of 2,3-dihydrofuran using 2-bromoaniline derivatives (GP1)

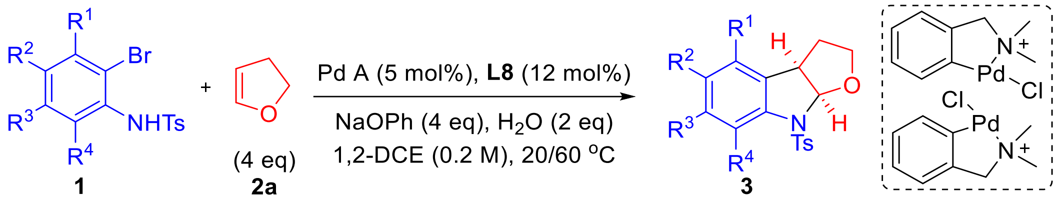

To a sealed tube was added Pd A (5 mol%), ***N*-Me-X5** (12 mol%). The flask was evacuated and refilled with argon. Then 2-Br-anilines **1** (0.2 mmol) and dry 1,2-DCE (1 mL) were added to the tube. NaOPh (4 eq) and H_2O (2 eq) were subsequently added under a flow of argon, followed by **2a** (4 eq). The mixture was stirred at 20 or 60 °C for 12-36 h. After the reaction was complete (monitored by TLC), solvent was removed under reduced pressure. The crude product was then purified by flash column chromatography on silica gel using hexane/EtOAc as the eluent to afford the desired product **3**.

3.3 General procedure for the intermolecular carboetherification of 2,3-dihydrofuran using 2-bromophenol derivatives (GP2)

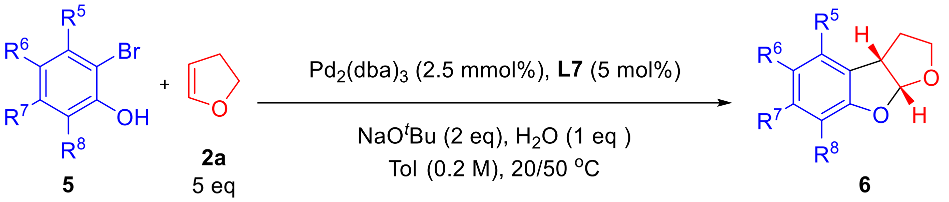

To a sealed tube was added $Pd_2(dba)_3$ (2.5 mol%), ***N*-Me-X4** (5 mol%). The flask was evacuated and refilled with argon. Then 2-Br-phenols **5** (0.3 mmol) and dry toluene (1.5 mL) were added to the tube. NaO^{t}Bu (2 eq) and H_2O (1 eq) were subsequently added under a flow of argon, followed by **2a** (5 eq). The mixture was stirred at 20 or 50 °C for 24-48 h. After the reaction was complete (monitored by TLC), solvent was removed under reduced pressure. The crude product was then purified by flash column chromatography on silica gel using hexane/Et_2O as the eluent to afford the desired product **6**.

3.4 General procedure for the synthesis of 2-substituted-2,3-dihydrofurans (GP3)[1]

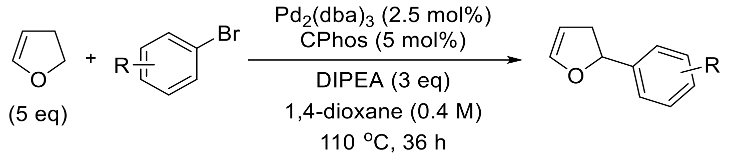

In a glovebox, a 50 mL Young valve Schlenk was charged with $Pd_2(dba)_3$ (126 mg, 0.138 mmol, 2.5 mol%), CPhos (120 mg, 0.275 mmol, 5 mol%) and distilled and degassed 1,4-dioxane (10 mL). The Schlenk was taken outside the glovebox, connected to a two-manifold line and the mixture was stirred at room temperature for 10 minutes. Next, the corresponding aryl bromide (5.5 mmol, 1 equiv.), DIPEA (2.8 mL, 16.5 mmol, 3.0 equiv.) and 2,3-dihydrofuran (2.0 mL, 27.5 mmol, 5 equiv.) were added consecutively under a flow of N_2 gas. The sealed reaction tube was immerged in an oil bath pre-heated at 110 ℃ for 36 h. After cooling to room temperature, the reaction mixture was poured into Et_2O (20 mL) under vigorous stirring and the resulting precipitate was removed passing the suspension through a short pad of Celite. The volatiles were evaporated and the resulting oil was directly subjected to flash chromatography (Pentane/Et_2O).

4. General Data for (*S*, *R*$_S$)-*N*-Me-X4/X5, 3 and 6

(*R*)-*N*-((*S*)-(3-(di((1*s*,3*R*,5*S*,7*S*)-adamantan-1-yl)phosphanyl)-5,5,8,8-tetramethyl-5,6,7,8-tetrahydronaphthalen-2-yl)(phenyl)methyl)-*N*,2-dimethylpropane-2-sulfinamide

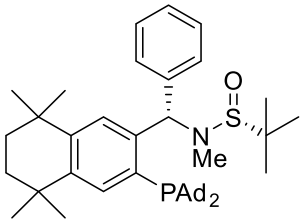

(*S*, *R*$_S$)-***N*-Me-X4**; colorless solid (hexane/EtOAc/DCM = 3:1:1, 38% overall yield); m.p. = 227-229 ^oC; $[\alpha]_D^{20}$ = 85.438 (*c* = 0.375, CH_2Cl_2); ^1H NMR (500 MHz, $CDCl_3$) δ 7.77 (d, *J* = 4.5 Hz, 1H), 7.60 (d, *J* = 2.1 Hz, 1H), 7.21 – 7.18 (m, 2H), 7.14 – 7.11 (m, 3H), 6.88 (d, *J* = 9.7 Hz, 1H), 2.58 (s, 3H), 1.98 (d, *J* = 11.9 Hz, 3H), 1.90 (s, 3H), 1.85 (d, *J* = 11.8 Hz, 3H), 1.73 (d, *J* = 2.8 Hz, 3H), 1.68 (d, *J* = 15.2 Hz, 10H), 1.50 (s, 6H), 1.44 (s, 6H), 1.39 (d, *J* = 19.3 Hz, 6H), 1.32 (s, 6H), 1.05 (s, 9H). ^{13}C NMR (126 MHz, $CDCl_3$) δ 145.69, 144.40 (d, *J* = 23.8 Hz), 141.43, 139.89, 135.50 (d, *J* = 2.6 Hz), 131.89, 129.09 (d, *J* = 25.4 Hz), 127.43, 126.80, 125.72 (d, *J* = 5.8 Hz), 70.94 (d, *J* = 33.3 Hz), 58.56, 41.83, 41.76 (dd, *J* = 12.6, 7.1 Hz), 41.68, 37.65, 37.47, 37.00, 36.82, 36.62, 36.44, 35.06 (d, *J* = 5.5 Hz), 34.34, 33.96, 31.83 (dd, *J* = 24.9, 15.2 Hz), 30.41, 28.80 (dd, *J* = 8.7, 6.1 Hz), 24.22. ^{31}P NMR (202 MHz, $CDCl_3$) δ 15.94. HRMS (ESI) m/z calcd. For $C_{46}H_{67}NOPS$ $[M+H]^+$ = 712.4675, found = 712.4666; IR spectrum (neat) (cm^{-1}) = 2980, 2909, 2359, 1198, 1167, 1086, 961, 949, 928, 880, 733, 669.

(*R*)-*N*-((*S*)-(3-(di((1*s*,3*R*,5*S*,7*S*)-adamantan-1-yl)phosphanyl)-5,5,8,8-tetramethyl-5,6,7,8-tetrahydonaphthalen-2-yl)(3,5-di-tert-butyl-4-methoxyphenyl)methyl)-*N*,2-dimethylpropane-2-sulfinamide

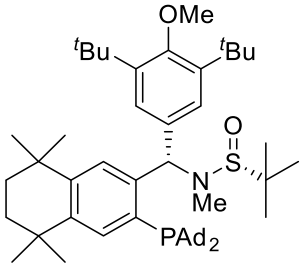

(*S*, *R*$_S$)-***N*-Me-X5**; colorless solid (hexane/EtOAc/DCM = 3:1:1, 31% overall yield); m.p. = 159-161 ^oC; $[\alpha]_D^{20}$ = 96.185 (*c* = 0.375, CH_2Cl_2); ^1H NMR (500 MHz, $CDCl_3$) δ 7.81 (d, *J* = 4.5 Hz, 1H), 7.60 (d, *J* = 2.1 Hz, 1H), 6.97 (s, 2H), 6.75 (d, *J* = 9.9 Hz, 1H), 3.57 (s, 3H), 2.59 (s, 3H), 1.99 (d, *J* = 11.9 Hz, 3H), 1.90– 1.85 (m, 6H), 1.76 – 1.71 (m, 4H), 1.69 – 1.64 (m, 6H), 1.52– 1.46 (m, 7H), 1.43 – 1.40 (m, 12H), 1.32 – 1.29 (m, 26H), 1.00 (s, 9H). ^{13}C NMR (126 MHz, $CDCl_3$) δ 158.23, 145.62, 144.58 (d, *J* = 23.9 Hz), 141.86, 141.19, 135.45 (d, *J* = 2.5 Hz), 133.51, 130.90, 129.12 (d, *J* = 25.4 Hz), 125.12 (d, *J* = 5.8 Hz), 71.12 (d, *J* = 34.4 Hz), 64.23, 58.40, 41.68 (dd, *J* = 12.8, 7.5 Hz), 37.51 (d, *J* = 23.3 Hz), 36.94 (d, *J*

= 22.0 Hz), 36.55 (d, J = 24.1 Hz), 35.57, 35.12, 34.41, 33.94, 32.45, 32.07, 32.02, 31.62, 31.42, 30.73, 28.82 (dd, J = 8.6, 6.9 Hz), 24.15. ^{31}P NMR (202 MHz, $CDCl_3$) δ 15.24. HRMS (ESI) m/z calcd. For $C_{55}H_{85}NO_2PS$ $[M+H]^+$ = 854.6033, found = 854.6048; IR spectrum (neat) (cm^{-1}) = 2895, 1450, 1362, 1250, 1198, 1167, 1088, 961, 930, 880, 777, 733.

(3a*R*,8a*R*)-8-tosyl-3,3a,8,8a-tetrahydro-2H-furo[2,3-*b*]indole

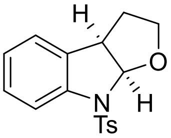

3aa; colorless solid (hexane/EtOAc = 8:1, 84% isolated yield); m.p. = 97-98 °C; $[\alpha]_D^{20}$ = 24.960 (c = 0.625, $CHCl_3$); ^{1}H NMR (500 MHz, $CDCl_3$) δ 7.86 (d, J = 8.3 Hz, 2H), 7.36 (d, J = 8.1 Hz, 1H), 7.24 (d, J = 8.2 Hz, 2H), 7.18 – 7.12 (m, 2H), 6.98 (t, J = 7.5 Hz, 1H), 6.26 (d, J = 6.6 Hz, 1H), 3.97 (t, J = 8.0 Hz, 1H), 3.90 (t, J = 7.5 Hz, 1H), 3.33 – 3.28 (m, 1H), 2.37 (s, 3H), 2.33 – 2.25 (m, 1H), 2.01 (dd, J = 12.2, 4.7 Hz, 1H). ^{13}C NMR (126 MHz, $CDCl_3$) δ 143.82, 141.43, 136.46, 131.32, 129.50, 128.30, 127.32, 124.83, 123.48, 112.74, 95.71, 66.35, 45.45, 33.62, 21.44. Enantiomeric excess: 96%, determined by HPLC (Chiralpak OJ-3, hexane/*i*-PrOH = 80/20; flow rate 0.8 ml/min; 25 ℃; 254 nm), first peak: t_R = 20.2 min, second peak: t_R = 28.4 min; HRMS (ESI) m/z calcd. for $C_{17}H_{17}NNaO_3S$ $[M+Na]^+$ = 338.0821, found = 338.0820; IR spectrum (neat) (cm^{-1}) = 2878, 1481, 1460, 1354, 1169, 1091, 949, 881, 752, 663.

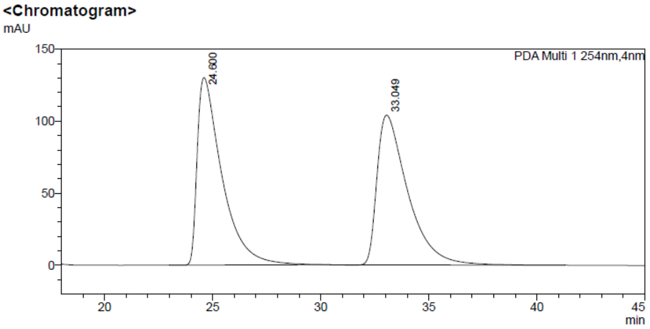

<Peak Table>
PDA Ch1 254nm

Peak#	Ret. Time	Height	Height%	Area	Area%
1	24.600	130241	55.604	10376534	50.105
2	33.049	103987	44.396	10333132	49.895
Total		234228	100.000	20709665	100.000

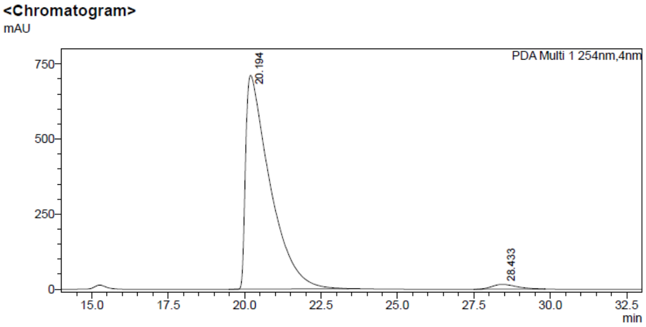

<Peak Table>
PDA Ch1 254nm

Peak#	Ret. Time	Height	Height%	Area	Area%
1	20.194	711984	97.831	39400130	97.804
2	28.433	15783	2.169	884579	2.196
Total		727767	100.000	40284709	100.000

(3a*R*,8a*R*)-5-fluoro-8-tosyl-3,3a,8,8a-tetrahydro-2H-furo[2,3-*b*]indole

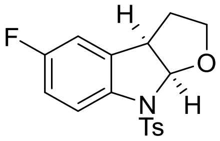

3ba; colorless solid (hexane/EtOAc = 8:1, 97% isolated yield); m.p. = 68-70 °C; $[\alpha]_D^{20}$ = 34.672 (c = 0.55, $CHCl_3$); ^{1}H NMR (500 MHz, $CDCl_3$) δ 7.81 (d, J = 8.3 Hz, 2H), 7.33 (dd, J = 8.8, 4.4 Hz, 1H), 7.25 (d, J = 8.1 Hz, 2H), 6.89 – 6.83 (m, 2H), 6.24 (d, J = 6.6 Hz, 1H), 3.98 (dd, J = 12.1, 4.2 Hz, 1H), 3.86 (t, J

= 7.6 Hz, 1H), 3.35 – 3.30 (m, 1H), 2.38 (s, 3H), 2.33 – 2.25 (m, 1H), 1.99 (dd, *J* = 12.3, 4.7 Hz, 1H). ^{13}C NMR (126 MHz, $CDCl_3$) δ 160.47, 158.55, 144.03, 137.51 (d, *J* = 2.0 Hz), 136.10, 133.41 (d, *J* = 8.1 Hz), 129.59, 127.25, 114.87 (d, *J* = 23.4 Hz), 113.89 (d, *J* = 8.3 Hz), 112.05 (d, *J* = 24.1 Hz), 96.22, 66.36, 45.47 (d, *J* = 1.7 Hz), 33.45, 21.46. ^{19}F NMR (376 MHz, $CDCl_3$) δ -119.61. Enantiomeric excess: 87%, determined by HPLC (Chiralpak OJ-3, hexane/*i*-PrOH = 70/30; flow rate 0.8 ml/min; 25 ℃; 254 nm), first peak: t_R = 19.0 min, second peak: t_R = 29.5 min; HRMS (ESI) m/z calcd. for $C_{17}H_{16}FNNaO_3S$ $[M+Na]^+$ = 356.0727, found = 356.0721; IR spectrum (neat) (cm^{-1}) = 2884, 1356, 1167, 1092, 961, 883, 814, 710, 669, 598.

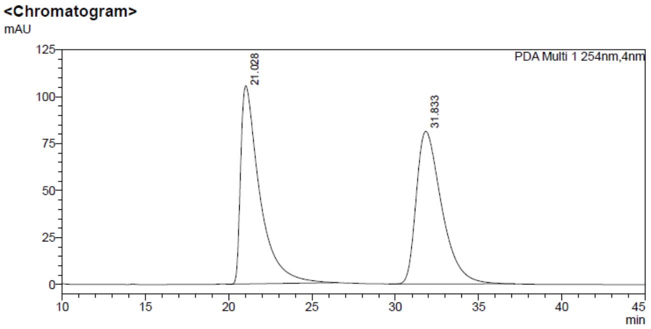

<Peak Table>
PDA Ch1 254nm

Peak#	Ret. Time	Height	Height%	Area	Area%
1	21.028	105513	56.491	8407652	49.286
2	31.833	81267	43.509	8651244	50.714
Total		186781	100.000	17058896	100.000

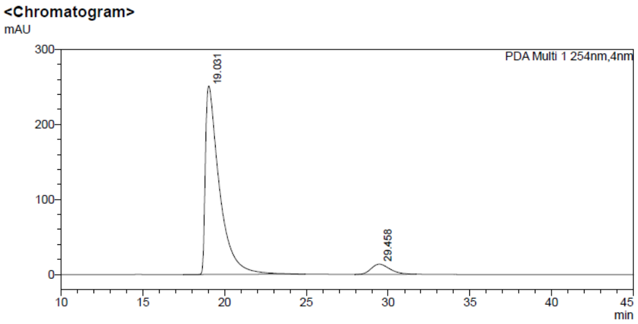

<Peak Table>
PDA Ch1 254nm

Peak#	Ret. Time	Height	Height%	Area	Area%
1	19.031	251464	94.870	15079259	93.303
2	29.458	13599	5.130	1082333	6.697
Total		265062	100.000	16161592	100.000

(3a*R*,8a*R*)-5-chloro-8-tosyl-3,3a,8,8a-tetrahydro-2H-furo[2,3-*b*]indole

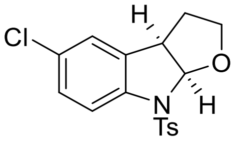

3ca; colorless solid (hexane/EtOAc = 8:1, 94% isolated yield); m.p. = 90-91 ^{o}C; $[\alpha]_D^{20}$ = 35.818 (*c* = 0.55, $CHCl_3$); ^{1}H NMR (500 MHz, $CDCl_3$) δ 7.83 (d, *J* = 8.3 Hz, 2H), 7.31 (d, *J* = 8.6 Hz, 1H), 7.27 – 7.25 (m, 2H), 7.13 (dd, *J* = 8.6, 1.9 Hz, 1H), 7.10 (s, 1H), 6.25 (d, *J* = 6.6 Hz, 1H), 3.98 (t, *J* = 8.0 Hz, 1H), 3.89 – 3.86 (m, 1H), 3.33 – 3.28 (m, 1H), 2.38 (s, 3H), 2.33 – 2.25 (m, 1H), 2.00 (dd, *J* = 12.3, 4.7 Hz, 1H). ^{13}C NMR (126 MHz, $CDCl_3$) δ 144.12, 140.17, 136.11, 133.32, 129.61, 128.32, 127.28, 125.04, 113.76, 96.05, 66.37, 45.32, 33.47, 21.47. Enantiomeric excess: 87%, determined by HPLC (Chiralpak OJ-3, hexane/*i*-PrOH = 80/20; flow rate 0.8 ml/min; 25 ℃; 254 nm), first peak: t_R = 26.5 min, second peak: t_R = 31.9 min; HRMS (ESI) m/z calcd. for $C_{17}H_{16}ClNNaO_3S$ $[M+Na]^+$ = 372.0432, found = 372.0423; IR spectrum (neat) (cm^{-1}) = 2884, 1356, 1167, 1090, 961, 930, 881, 669, 590.

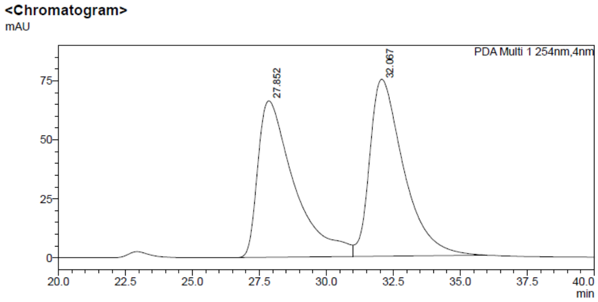

<Peak Table>
PDA Ch1 254nm

Peak#	Ret. Time	Height	Height%	Area	Area%
1	27.852	66270	46.929	6278736	48.654
2	32.067	74944	53.071	6626171	51.346
Total		141214	100.000	12904908	100.000

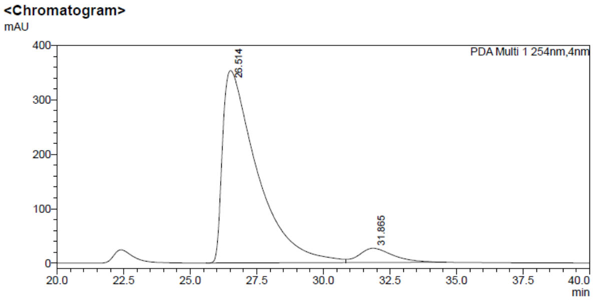

<Peak Table>
PDA Ch1 254nm

Peak#	Ret. Time	Height	Height%	Area	Area%
1	26.514	353693	93.133	32223977	93.308
2	31.865	26081	6.867	2311244	6.692
Total		379774	100.000	34535221	100.000

(3a*R*,8a*R*)-5-methyl-8-tosyl-3,3a,8,8a-tetrahydro-2H-furo[2,3-*b*]indole

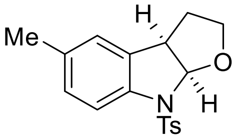

3da; amorphous colorless solid (hexane/EtOAc = 8:1, 95% isolated yield); m.p. = 53-54 ºC; $[\alpha]_D^{20}$ = 49.781 (*c* = 0.55, $CHCl_3$); ^{1}H NMR (500 MHz, $CDCl_3$) δ 7.83 (d, *J* = 8.3 Hz, 2H), 7.27 (d, *J* = 8.0 Hz, 1H), 7.23 (d, *J* = 8.1 Hz, 2H), 6.97 (d, *J* = 8.3 Hz, 1H), 6.93 (s, 1H), 6.21 (d, *J* = 6.6 Hz, 1H), 3.95 (t, *J* = 7.9 Hz, 1H), 3.84 (t, *J* = 7.5 Hz, 1H), 3.34 – 3.29 (m, 1H), 2.36 (s, 3H), 2.30 – 2.22 (m, 4H), 2.00 (dd, *J* = 12.2, 4.7 Hz, 1H). ^{13}C NMR (126 MHz, $CDCl_3$) δ 143.70, 139.14, 136.42, 133.21, 131.46, 129.47, 128.84, 127.27, 125.40, 112.70, 95.91, 66.37, 45.45, 33.57, 21.43, 20.77. Enantiomeric excess: 95%, determined by HPLC (Chiralpak OJ-3, hexane/*i*-PrOH = 70/30; flow rate 0.8 ml/min; 25 ℃; 254 nm), first peak: t_R = 15.7 min, second peak: t_R = 22.9 min; HRMS (ESI) m/z calcd. for $C_{18}H_{19}NNaO_3S$ $[M+Na]^+$ = 352.0978, found = 352.0975; IR spectrum (neat) (cm^{-1}) = 2880, 1599, 1354, 1165, 1092, 991, 880, 814, 708, 662, 578.

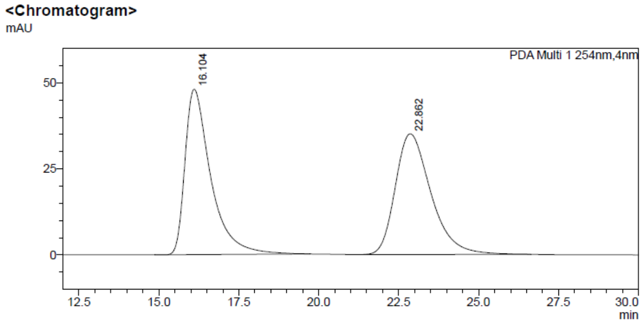

<Peak Table>
PDA Ch1 254nm

Peak#	Ret. Time	Height	Height%	Area	Area%
1	16.104	48083	57.810	2700282	49.776
2	22.862	35091	42.190	2724602	50.224
Total		83173	100.000	5424884	100.000

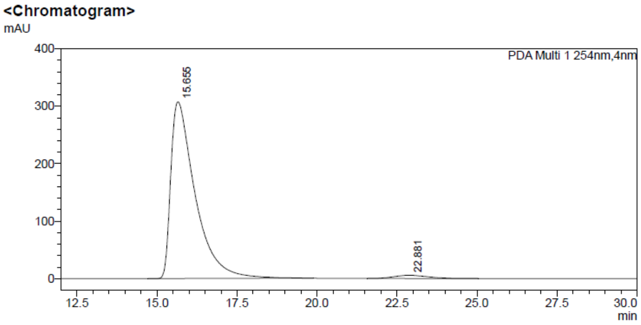

<Peak Table>
PDA Ch1 254nm

Peak#	Ret. Time	Height	Height%	Area	Area%
1	15.655	307554	98.260	16312383	97.566
2	22.881	5445	1.740	406989	2.434
Total		312999	100.000	16719372	100.000

(3a*R*,8a*R*)-5-methoxy-8-tosyl-3,3a,8,8a-tetrahydro-2H-furo[2,3-*b*]indole

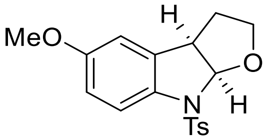

3ea; colorless solid (hexane/EtOAc = 5:1, 93% isolated yield); m.p. = 151-153 ºC; $[\alpha]_D^{20}$ = 81.647 (*c* = 0.54, $CHCl_3$); ^1H NMR (500 MHz, $CDCl_3$) δ 7.78 (d, *J* = 8.2 Hz, 2H), 7.33 (d, *J* = 8.8 Hz, 1H), 7.22 (d, *J* = 8.2 Hz, 2H), 6.72 (dd, *J* = 8.8, 2.6 Hz, 1H), 6.68 (d, *J* = 2.4 Hz, 1H), 6.17 (d, *J* = 6.5 Hz, 1H), 3.96 (t, *J* = 8.1 Hz, 1H), 3.82 – 3.79 (m, 1H), 3.74 (s, 3H), 3.37 – 3.32 (m, 1H), 2.36 (s, 3H), 2.30 – 2.22 (m, 1H), 2.00 (dd, *J* = 12.2, 4.8 Hz, 1H). ^{13}C NMR (126 MHz, $CDCl_3$) δ 156.59, 143.73, 136.15, 134.95, 133.08, 129.48, 127.19, 114.06, 113.24, 110.77, 96.15, 66.38, 55.56, 45.64, 33.46, 21.43. Enantiomeric excess: 90%, determined by HPLC (Chiralpak OJ-3, hexane/*i*-PrOH = 70/30; flow rate 0.8 ml/min; 25 ℃; 254 nm), first peak: t_R = 26.7 min, second peak: t_R = 44.4 min; HRMS (ESI) m/z calcd. for $C_{18}H_{19}NNaO_4S$ $[M+Na]^+$ = 368.0927, found = 368.0919; IR spectrum (neat) (cm^{-1}) = 2884, 1198, 1084, 961, 928, 881, 733, 669.

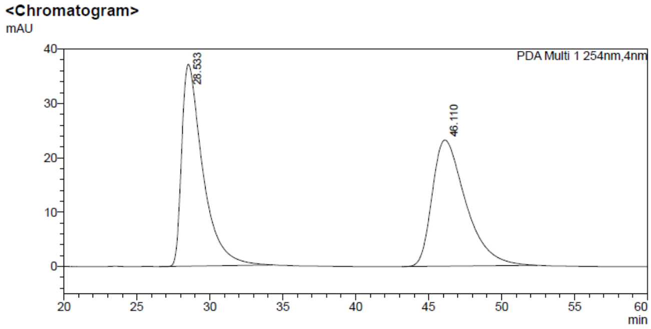

<Peak Table>
PDA Ch1 254nm

Peak#	Ret. Time	Height	Height%	Area	Area%
1	28.533	37103	61.532	3727114	50.053
2	46.110	23196	38.468	3719208	49.947
Total		60299	100.000	7446321	100.000

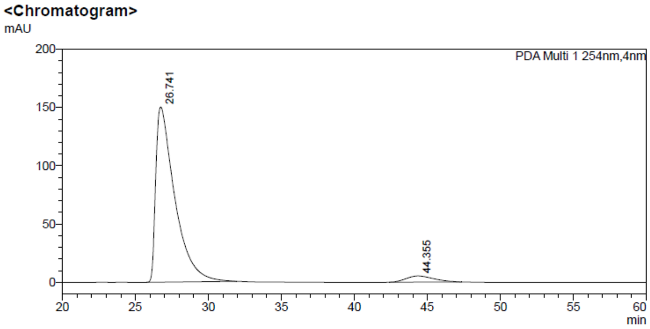

<Peak Table>
PDA Ch1 254nm

Peak#	Ret. Time	Height	Height%	Area	Area%
1	26.741	150318	96.628	13597306	95.165
2	44.355	5245	3.372	690883	4.835
Total		155564	100.000	14288189	100.000

(3a*R*,8a*R*)-8-tosyl-5-(trifluoromethyl)-3,3a,8,8a-tetrahydro-2H-furo[2,3-*b*]indole

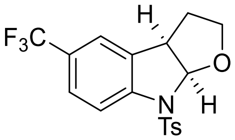

3fa; amorphous colorless solid (hexane/EtOAc = 8:1, 96% isolated yield); m.p. = 52-53 ºC; $[\alpha]_D^{20}$ = 4.896 (*c* = 0.625, $CHCl_3$); ^1H NMR (500 MHz, $CDCl_3$) δ 7.89 (d, *J* = 8.4 Hz, 2H), 7.45 – 7.41 (m, 2H), 7.38 (s, 1H), 7.28 (d, *J* = 8.1 Hz, 2H), 6.35 (d, *J* = 6.6 Hz, 1H), 4.02 – 3.96 (m, 2H), 3.31 – 3.26 (m, 1H), 2.39 (s, 3H), 2.37 – 2.230 (m, 1H), 2.05 (dd, *J* = 12.4, 4.6 Hz, 1H). ^{13}C NMR (126 MHz, $CDCl_3$) δ 144.38, 136.20, 132.02, 129.70, 127.41, 126.05 (q, *J* = 3.9 Hz), 125.50 (q, *J* = 32.6 Hz), 124.08 (q, *J* = 271.6 Hz), 122.08 (q, *J* = 3.7 Hz), 112.16, 96.13, 66.38, 45.26, 33.60, 21.50. ^{19}F NMR (376 MHz, $CDCl_3$) δ -61.64. Enantiomeric excess: 94%, determined by HPLC (Chiralpak AD-H, hexane/*i*-PrOH = 80/20; flow rate

0.8 ml/min; 25 ℃; 254 nm), first peak: t_R = 11.2 min, second peak: t_R = 14.5 min; HRMS (ESI) m/z calcd. for $C_{18}H_{16}F_3NNaO_3S$ $[M+Na]^+$ = 406.0695, found = 406.0692; IR spectrum (neat) (cm^{-1}) = 2880, 1620, 1445, 1337, 1285, 1167, 1121, 1078, 989, 961, 877, 721, 664, 596.

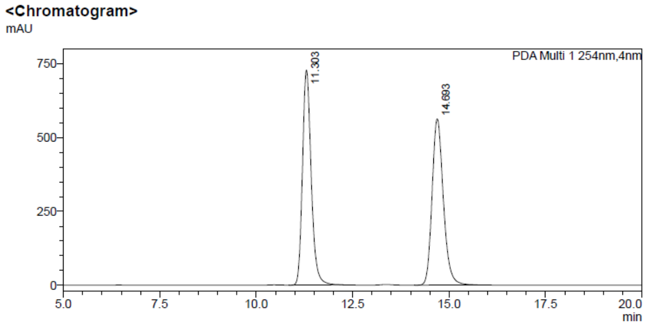

<Peak Table>
PDA Ch1 254nm

Peak#	Ret. Time	Height	Height%	Area	Area%
1	11.303	728323	56.404	11446117	49.974
2	14.693	562938	43.596	11457997	50.026
Total		1291261	100.000	22904114	100.000

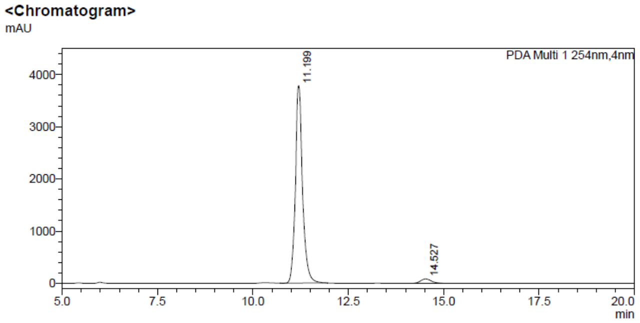

<Peak Table>
PDA Ch1 254nm

Peak#	Ret. Time	Height	Height%	Area	Area%
1	11.199	3785788	97.809	49396923	96.779
2	14.527	84789	2.191	1643813	3.221
Total		3870577	100.000	51040736	100.000

(3a*R*,8a*R*)-8-tosyl-5-(trifluoromethoxy)-3,3a,8,8a-tetrahydro-2H-furo[2,3-*b*]indole

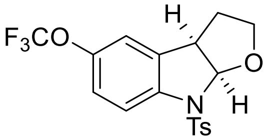

3ga; colorless solid (hexane/EtOAc = 8:1, 87% isolated yield); m.p. = 46-48 ^oC; $[\alpha]_D^{20}$ = 12.339 (*c* = 0.53, $CHCl_3$); ^1H NMR (500 MHz, $CDCl_3$) δ 7.86 (d, *J* = 8.3 Hz, 2H), 7.35 (d, *J* = 8.7 Hz, 1H), 7.28 (d, *J* = 9.1 Hz, 2H), 7.03 – 7.00 (m, 2H), 6.30 (d, *J* = 6.6 Hz, 1H), 4.00 (t, *J* = 8.0 Hz, 1H), 3.92 (t, *J* = 7.6 Hz, 1H), 3.35 – 3.30 (m, 1H), 2.39 (s, 3H), 2.36 – 2.28 (m, 1H), 2.01 (dd, *J* = 12.4, 4.7 Hz, 1H). ^{13}C NMR (126 MHz, $CDCl_3$) δ 145.11 (d, *J* = 1.8 Hz), 144.21, 140.14, 136.23, 133.14, 129.67, 127.36, 121.33, 120.38 (q, *J* = 256.8 Hz), 118.15, 113.21, 96.20, 66.37, 45.39, 33.55, 21.49. ^{19}F NMR (376 MHz, $CDCl_3$) δ -58.24. Enantiomeric excess: 87%, determined by HPLC (Chiralpak OJ-3, hexane/*i*-PrOH = 80/20; flow rate 0.8 ml/min; 25 ℃; 254 nm), first peak: t_R = 15.0 min, second peak: t_R = 19.6 min; HRMS (ESI) m/z calcd. for $C_{18}H_{16}F_3NNaO_4S$ $[M+Na]^+$ = 422.0644, found = 422.0639; IR spectrum (neat) (cm^{-1}) = 2874, 1599, 1485, 1357, 1250, 1161, 1094, 991, 872, 814, 662, 586.

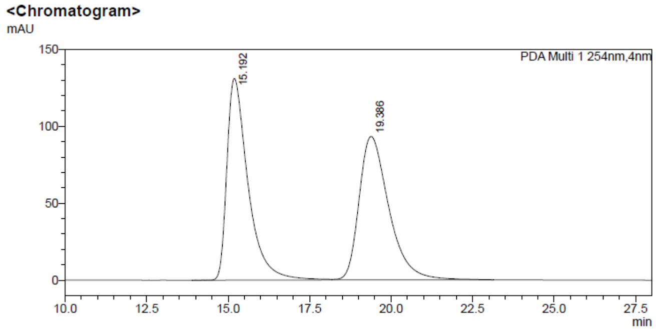

<Peak Table>
PDA Ch1 254nm

Peak#	Ret. Time	Height	Height%	Area	Area%
1	15.192	131112	58.421	5919332	49.942
2	19.386	93316	41.579	5933087	50.058
Total		224428	100.000	11852419	100.000

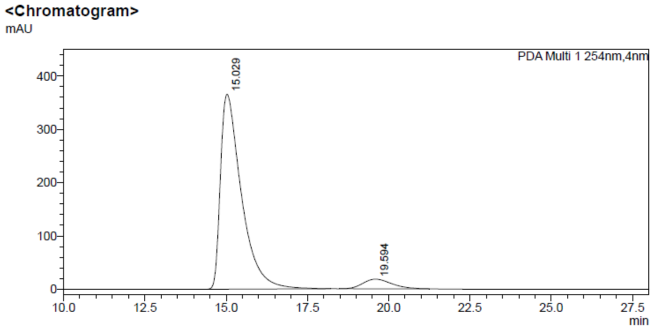

<Peak Table>
PDA Ch1 254nm

Peak#	Ret. Time	Height	Height%	Area	Area%
1	15.029	365479	95.259	16405356	93.588
2	19.594	18188	4.741	1123974	6.412
Total		383667	100.000	17529330	100.000

(3a*R*,8a*R*)-6-fluoro-8-tosyl-3,3a,8,8a-tetrahydro-2H-furo[2,3-*b*]indole

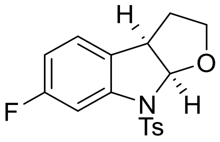

3ha; colorless solid (hexane/EtOAc = 8:1, 75% isolated yield); m.p. = 51-53 °C; $[\alpha]_D^{20}$ = 10.2 (c = 0.5, $CHCl_3$); ^{1}H NMR (500 MHz, $CDCl_3$) δ 7.87 (d, J = 8.4 Hz, 2H), 7.27 (d, J = 9.2 Hz, 2H), 7.10 (dd, J = 9.9, 2.3 Hz, 1H), 7.06 – 7.01 (m, 1H), 6.67 (td, J = 8.6, 2.3 Hz, 1H), 6.29 (d, J = 6.6 Hz, 1H), 3.97 (t, J = 8.1 Hz, 1H), 3.87 (t, J = 7.4 Hz, 1H), 3.32 – 3.27 (m, 1H), 2.39 (s, 3H), 2.31 – 2.23 (m, 1H), 1.98 (dd, J = 12.2, 4.7 Hz, 1H). ^{13}C NMR (126 MHz, $CDCl_3$) δ 163.89, 161.94, 144.18, 142.73 (d, J = 11.9 Hz), 136.26, 129.65, 127.37, 126.69 (d, J = 2.6 Hz), 125.52 (d, J = 10.0 Hz), 110.02 (d, J = 22.9 Hz), 100.99 (d, J = 28.6 Hz), 96.59, 66.40, 44.93, 33.74, 21.50. ^{19}F NMR (282 MHz, $CDCl_3$) δ -112.53. Enantiomeric excess: 95%, determined by HPLC (Chiralpak OJ-3, hexane/*i*-PrOH = 70/30; flow rate 0.8 ml/min; 25 ℃; 254 nm), first peak: t_R = 13.5 min, second peak: t_R = 16.5 min; HRMS (ESI) m/z calcd. for $C_{17}H_{16}FNNaO_3S$ $[M+Na]^+$ = 356.0727, found = 356.0719; IR spectrum (neat) (cm^{-1}) = 2874, 1603, 1437, 1350, 1161, 1143, 1099, 999, 864, 813, 706, 664, 583.

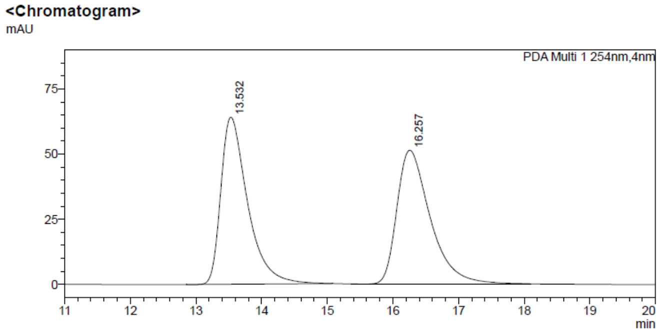

<Peak Table>

PDA Ch1 254nm

Peak#	Ret. Time	Area	Height	Conc.	Unit	Mark	Name
1	13.532	1817253	64110	0.000		M	
2	16.257	1808848	51278	0.000		M	
Total		3626100	115389				

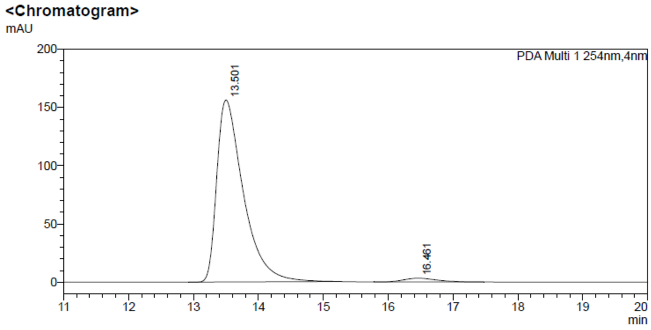

<Peak Table>

PDA Ch1 254nm

Peak#	Ret. Time	Height	Height%	Area	Area%
1	13.501	156241	98.004	4461526	97.547
2	16.461	3182	1.996	112175	2.453
Total		159423	100.000	4573701	100.000

(3a*R*,8a*R*)-6-chloro-8-tosyl-3,3a,8,8a-tetrahydro-2H-furo[2,3-*b*]indole

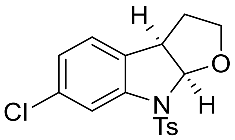

3ia; colorless solid (hexane/EtOAc = 8:1, 67% isolated yield); m.p. = 93-94 °C; $[\alpha]_D^{20}$ = 10.782 (c = 0.46, $CHCl_3$); ^{1}H NMR (500 MHz, $CDCl_3$) δ 7.87 (d, J = 8.3 Hz, 2H), 7.37 (d, J = 1.8 Hz, 1H), 7.28 (d, J = 8.1 Hz, 2H), 7.04 (d, J = 8.0 Hz, 1H), 6.95 (dd, J = 8.0, 1.8 Hz, 1H), 6.27 (d, J = 6.6 Hz, 1H), 3.97 (t, J = 8.0 Hz, 1H), 3.89 – 3.86 (m, 1H), 3.30 – 3.25 (m, 1H), 2.40 (s, 3H), 2.32 – 2.24 (m, 1H), 1.97 (dd, J = 12.3, 4.7 Hz, 1H). ^{13}C NMR (126 MHz, $CDCl_3$) δ 144.20, 142.58, 136.25, 134.10, 129.85, 129.68, 127.36,

125.60, 123.48, 113.05, 96.29, 66.37, 45.08, 33.63, 21.52. Enantiomeric excess: 92%, determined by HPLC (Chiralpak OJ-3, hexane/*i*-PrOH = 70/30; flow rate 0.8 ml/min; 25 ℃; 254 nm), first peak: t_R = 13.9 min, second peak: t_R = 18.5 min; HRMS (ESI) m/z calcd. for $C_{17}H_{16}ClNNaO_3S$ $[M+Na]^+$ = 372.0432, found = 372.0420; IR spectrum (neat) (cm^{-1}) = 2874, 1418, 1356, 1169, 1092, 1078, 993, 961, 881, 665, 583.

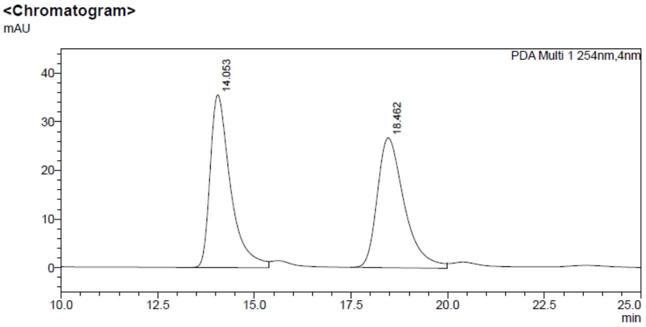

<Peak Table>
PDA Ch1 254nm

Peak#	Ret. Time	Height	Height%	Area	Area%
1	14.053	35490	57.075	1299549	50.002
2	18.462	26692	42.925	1299456	49.998
Total		62182	100.000	2599005	100.000

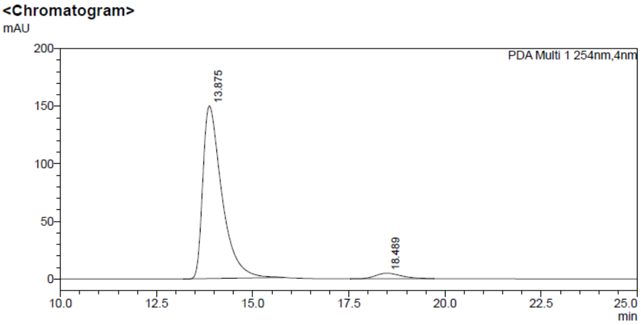

<Peak Table>
PDA Ch1 254nm

Peak#	Ret. Time	Height	Height%	Area	Area%
1	13.875	149955	96.925	5183778	95.913
2	18.489	4757	3.075	220913	4.087
Total		154712	100.000	5404691	100.000

(3a*R*,8a*R*)-6-methyl-8-tosyl-3,3a,8,8a-tetrahydro-2H-furo[2,3-*b*]indole

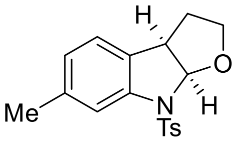

3ja; amorphous colorless solid (hexane/EtOAc = 8:1, 66% isolated yield); m.p. = 98-99 ^oC; $[\alpha]_D^{20}$ = 21.220 (*c* = 0.5, $CHCl_3$); ^1H NMR (500 MHz, $CDCl_3$) δ 7.86 (d, *J* = 8.3 Hz, 2H), 7.25 (d, J = 9.4 Hz, 2H), 7.20 (s, 1H), 7.00 (d, *J* = 7.6 Hz, 1H), 6.80 (d, *J* = 7.6 Hz, 1H), 6.24 (d, *J* = 6.6 Hz, 1H), 3.95 (t, *J* = 8.0 Hz, 1H), 3.85 (t, *J* = 7.5 Hz, 1H), 3.34 – 3.28 (m, 1H), 2.38 (s, 3H), 2.31 (s, 3H), 2.28 – 2.23 (m, 1H), 1.98 (dd, *J* = 12.2, 4.6 Hz, 1H). ^{13}C NMR (126 MHz, $CDCl_3$) δ 143.76, 141.62, 138.50, 136.67, 129.52, 128.42, 127.33, 124.44, 124.29, 113.49, 96.08, 66.38, 45.18, 33.72, 21.66, 21.49. Enantiomeric excess: 93%, determined by HPLC (Chiralpak OJ-3, hexane/*i*-PrOH = 70/30; flow rate 0.8 ml/min; 25 ℃; 254 nm), first peak: t_R = 11.6 min, second peak: t_R = 17.1 min; HRMS (ESI) m/z calcd. for $C_{18}H_{19}NNaO_3S$ $[M+Na]^+$ = 352.0978, found = 352.0975; IR spectrum (neat) (cm^{-1}) = 2886, 1612, 1493, 1350, 1165, 1094, 961, 928, 814, 733, 665, 584.

<Chromatogram>

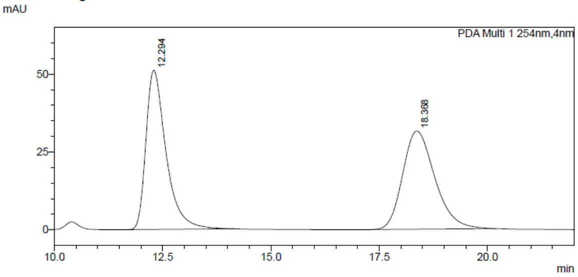

<Peak Table>

PDA Ch1 254nm

Peak#	Ret. Time	Height	Height%	Area	Area%
1	12.294	51110	61.849	1651408	50.288
2	18.368	31526	38.151	1632495	49.712
Total		82636	100.000	3283902	100.000

<Chromatogram>

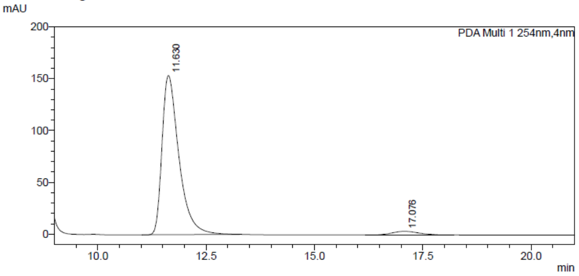

<Peak Table>

PDA Ch1 254nm

Peak#	Ret. Time	Height	Height%	Area	Area%
1	11.630	153725	97.738	4174517	96.472
2	17.076	3557	2.262	152657	3.528
Total		157282	100.000	4327173	100.000

methyl (3a*R*,8a*R*)-8-tosyl-3,3a,8,8a-tetrahydro-2H-furo[2,3-*b*]indole-6-carboxylate

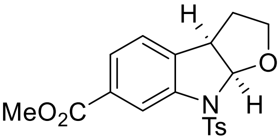

3ka; colorless solid (hexane/EtOAc = 5:1, 72% isolated yield); m.p. = 173-175 °C; $[\alpha]_D^{20}$ = 16.8 (*c* = 0.625, $CHCl_3$); ^1H NMR (500 MHz, $CDCl_3$) δ 8.00 (d, *J* = 1.1 Hz, 1H), 7.89 (d, *J* = 8.3 Hz, 2H), 7.71 (dd, *J* = 7.8, 1.3 Hz, 1H), 7.27 (d, *J* = 10 Hz, 2H), 7.20 (d, *J* = 7.8 Hz, 1H), 6.32 (d, *J* = 6.6 Hz, 1H), 4.00 – 3.93 (m, 2H), 3.91 (s, 3H), 3.30 – 3.24 (m, 1H), 2.38 (s, 3H), 2.36 – 2.29 (m, 1H), 2.03 (dd, *J* = 12.0, 4.4 Hz, 1H). ^{13}C NMR (126 MHz, $CDCl_3$) δ 166.49, 144.12, 141.82, 136.59, 136.24, 130.66, 129.63, 127.41, 125.25, 124.72, 113.33, 95.99, 66.34, 52.23, 45.50, 33.52, 21.50. Enantiomeric excess: 80%, determined by HPLC (Chiralpak OJ-3, hexane/*i*-PrOH = 60/40; flow rate 0.8 ml/min; 25 ℃; 254 nm), first peak: t_R = 21.1 min, second peak: t_R = 40.0 min; HRMS (ESI) m/z calcd. for $C_{19}H_{19}NNaO_5S$ $[M+Na]^+$ = 396.0876, found = 396.0866; IR spectrum (neat) (cm^{-1}) = 2884, 1368, 1088, 961, 928, 881, 750, 665, 586.

<Chromatogram>

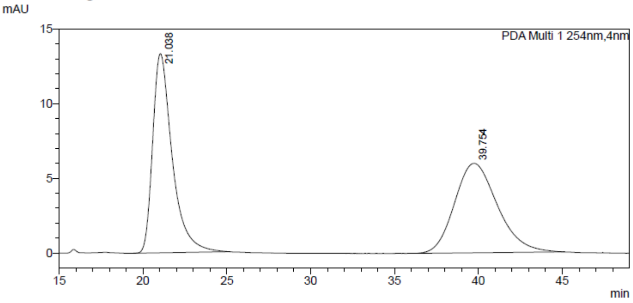

<Peak Table>

PDA Ch1 254nm

Peak#	Ret. Time	Area	Height	Conc.	Unit	Mark	Name
1	21.038	1080711	13341	0.000		M	
2	39.754	1065597	5993	0.000		M	
Total		2146308	19334				

<Chromatogram>

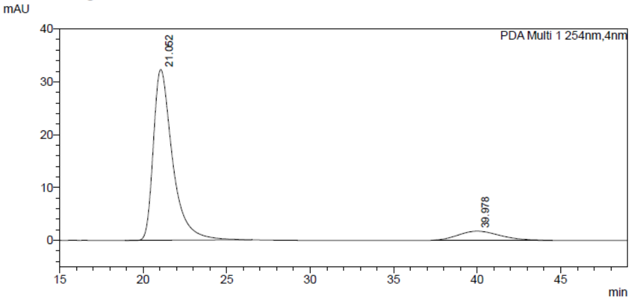

<Peak Table>

PDA Ch1 254nm

Peak#	Ret. Time	Height	Height%	Area	Area%
1	21.052	32314	94.952	2585326	89.731
2	39.978	1718	5.048	295858	10.269
Total		34032	100.000	2881184	100.000

(3a*R*,8a*R*)-8-tosyl-6-(trifluoromethyl)-3,3a,8,8a-tetrahydro-2H-furo[2,3-*b*]indole

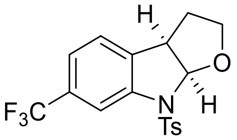

3la; colorless solid (hexane/EtOAc = 8:1, 84% isolated yield); m.p. = 124-126 °C; $[\alpha]_D^{20}$ = 2.8 (*c* = 0.5, $CHCl_3$); ^1H NMR (500 MHz, $CDCl_3$) δ 7.87 (d, *J* = 8.3 Hz, 2H), 7.61 (s, 1H), 7.29 – 7.24 (m, 4H), 6.32 (d, *J* = 6.6 Hz, 1H), 4.01 – 3.94 (m, 2H), 3.30 – 3.25 (m, 1H), 2.39 (s, 3H), 2.36 – 2.30 (m, 1H), 2.02 (dd, *J* = 12.5, 4.9 Hz, 1H). ^{13}C NMR (126 MHz, $CDCl_3$) δ 144.37, 142.02, 136.07, 135.33, 130.88 (q, *J* = 32.4 Hz), 129.71, 127.39, 125.22, 123.82 (q, *J* = 272.5 Hz), 120.46 (q, *J* = 3.9 Hz), 109.43 (q, *J* = 3.9 Hz), 96.07, 66.36, 45.40, 33.55, 21.50. ^{19}F NMR (376 MHz, $CDCl_3$) δ -62.34. Enantiomeric excess: 85%, determined by HPLC (Chiralpak OJ-3, hexane/*i*-PrOH = 70/30; flow rate 0.8 ml/min; 25 ℃; 254 nm), first peak: t_R = 10.3 min, second peak: t_R = 13.0 min; HRMS (ESI) m/z calcd. for $C_{18}H_{16}F_3NNaO_3S$ $[M+Na]^+$ = 406.0695, found = 406.0691; IR spectrum (neat) (cm^{-1}) = 2884, 1435, 1361, 1317, 1168, 1121, 1092, 1078, 961, 732, 664.

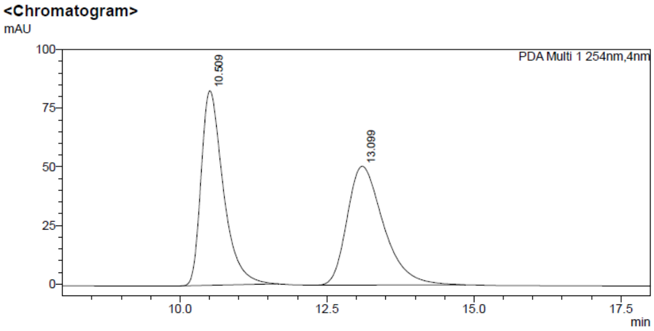

<Peak Table>
PDA Ch1 254nm

Peak#	Ret. Time	Height	Height%	Area	Area%
1	10.509	83016	62.094	2184719	50.015
2	13.099	50679	37.906	2183448	49.985
Total		133694	100.000	4368167	100.000

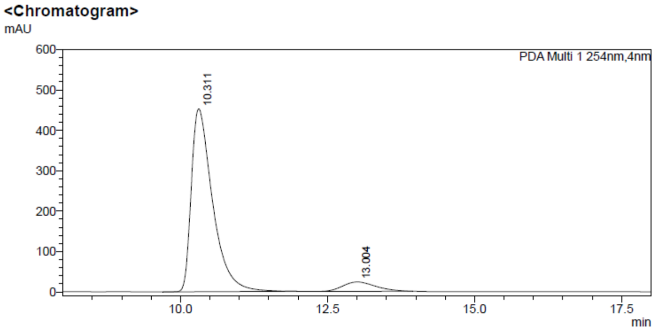

<Peak Table>
PDA Ch1 254nm

Peak#	Ret. Time	Height	Height%	Area	Area%
1	10.311	452849	95.109	11424898	92.615
2	13.004	23286	4.891	910990	7.385
Total		476135	100.000	12335888	100.000

(3a*R*,8a*R*)-4-fluoro-8-tosyl-3,3a,8,8a-tetrahydro-2H-furo[2,3-*b*]indole

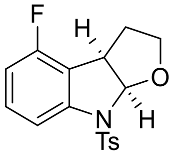

3ma; colorless solid (hexane/EtOAc = 8:1, 81% isolated yield); m.p. = 94-95 °C; $[\alpha]_D^{20}$ = 17.232 (*c* = 0.625, $CHCl_3$); ^1H NMR (500 MHz, $CDCl_3$) δ 7.86 (d, *J* = 8.4 Hz, 2H), 7.27 – 7.26 (m, 2H), 7.18 – 7.12 (m, 2H), 6.69 – 6.65 (m, 1H), 6.30 (d, *J* = 6.7 Hz, 1H), 4.02 – 3.99 (m, 2H), 3.36 – 3.31 (m, 1H), 2.38 (s, 3H), 2.28 – 2.20 (m, 1H), 2.14 (dd, *J* = 12.5, 4.9 Hz, 1H). ^{13}C NMR (126 MHz, $CDCl_3$) δ 160.00, 158.03, 144.11, 143.60 (d, *J* = 8.4 Hz), 136.21, 130.18 (d, *J* = 8.4 Hz), 129.58, 127.33, 117.42 (d, *J* = 20.6 Hz), 110.18 (d, *J* = 20.0 Hz), 108.49 (d, *J* = 3.3 Hz), 96.22, 66.47, 42.77, 31.84, 21.47. ^{19}F NMR (376 MHz, $CDCl_3$) δ -118.56. Enantiomeric excess: 93%, determined by HPLC (Chiralpak OJ-3, hexane/*i*-PrOH = 70/30; flow rate 0.8 ml/min; 25 ℃; 254 nm), first peak: t_R = 14.3 min, second peak: t_R = 18.9 min; HRMS

(ESI) m/z calcd. for $C_{17}H_{16}FNNaO_3S$ $[M+Na]^+$ = 356.0727, found = 356.0724; IR spectrum (neat) (cm^{-1}) = 2897, 1626, 1362, 1240, 1171, 1088, 961, 881, 777, 733, 664.

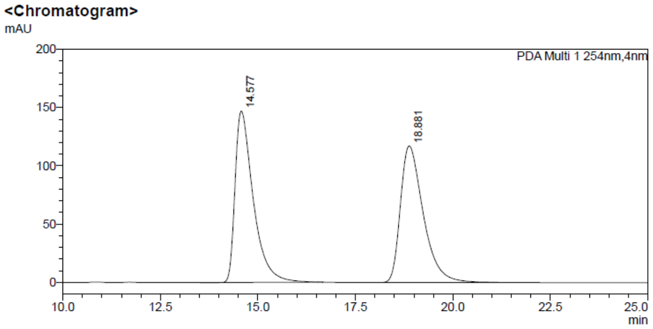

<Peak Table>
PDA Ch1 254nm

Peak#	Ret. Time	Height	Height%	Area	Area%
1	14.577	147176	55.657	4775907	49.979
2	18.881	117256	44.343	4779971	50.021
Total		264432	100.000	9555878	100.000

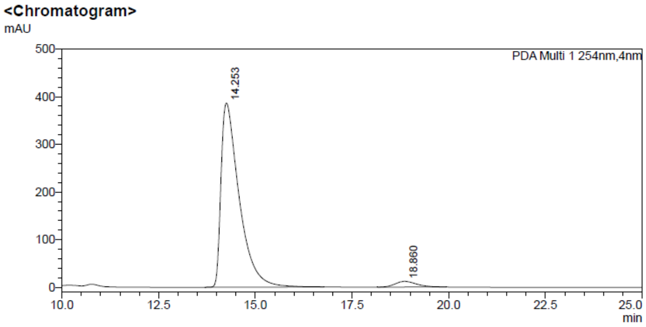

<Peak Table>
PDA Ch1 254nm

Peak#	Ret. Time	Height	Height%	Area	Area%
1	14.253	386752	97.023	12618785	96.477
2	18.860	11868	2.977	460726	3.523
Total		398620	100.000	13079511	100.000

(3a*R*,8a*R*)-4-methyl-8-tosyl-3,3a,8,8a-tetrahydro-2H-furo[2,3-*b*]indole

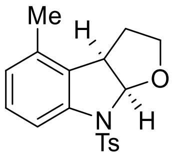

3na; amorphous colorless solid (hexane/EtOAc = 8:1, 51% isolated yield); m.p. = 57-59 °C; $[\alpha]_D^{20}$ = 7.44 (c = 0.5, $CHCl_3$); ^1H NMR (500 MHz, $CDCl_3$) δ 7.85 (d, J = 8.3 Hz, 2H), 7.24 (dd, J = 8.3, 2.7 Hz, 3H), 7.08 (t, J = 7.9 Hz, 1H), 6.79 (d, J = 7.6 Hz, 1H), 6.29 (d, J = 6.9 Hz, 1H), 4.00 – 3.96 (m, 1H), 3.89 – 3.85 (m, 1H), 3.41 – 3.36 (m, 1H), 2.37 (s, 3H), 2.31 – 2.22 (m, 4H), 1.97 (dd, J = 12.2, 5.1 Hz, 1H). ^{13}C NMR (126 MHz, $CDCl_3$) δ 143.81, 141.30, 136.47, 134.52, 129.54, 128.34, 127.38, 124.76, 110.20, 95.91, 65.93, 44.65, 32.19, 21.51, 18.50. Enantiomeric excess: 93%, determined by HPLC (Chiralpak OJ-3, hexane/*i*-PrOH = 70/30; flow rate 0.8 ml/min; 25 ℃; 254 nm), first peak: t_R = 13.9 min, second peak: t_R = 20.0 min; HRMS (ESI) m/z calcd. for $C_{18}H_{19}NNaO_3S$ $[M+Na]^+$ = 352.0978, found = 352.0972; IR spectrum (neat) (cm^{-1}) = 2886, 1458, 1356, 1250, 1167, 1084, 1051, 961, 927, 881, 775, 662, 578.

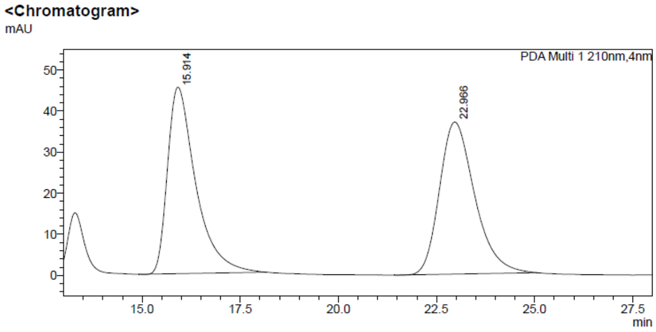

<Peak Table>
PDA Ch1 210nm

Peak#	Ret. Time	Height	Height%	Area	Area%
1	15.914	45410	55.085	2276770	49.608
2	22.966	37027	44.915	2312714	50.392
Total		82437	100.000	4589484	100.000

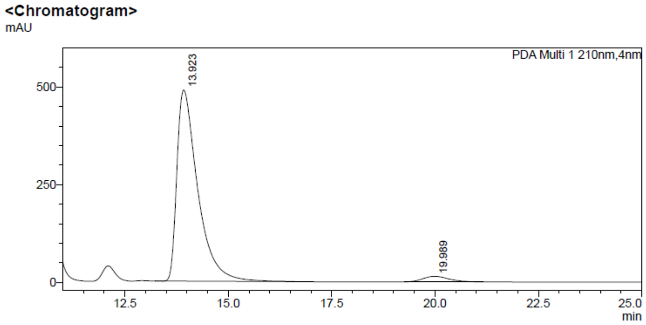

<Peak Table>
PDA Ch1 210nm

Peak#	Ret. Time	Height	Height%	Area	Area%
1	13.923	488811	97.145	17137530	96.481
2	19.989	14366	2.855	625046	3.519
Total		503177	100.000	17762576	100.000

(3a*R*,8a*R*)-7-fluoro-8-tosyl-3,3a,8,8a-tetrahydro-2H-furo[2,3-*b*]indole

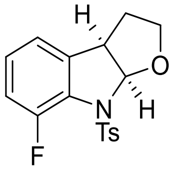

3oa; colorless solid (hexane/EtOAc = 8:1, 66% isolated yield); m.p. = 66-67 °C; $[\alpha]_D^{20}$ = 3.18 (*c* = 0.5, $CHCl_3$); ^{1}H NMR (500 MHz, $CDCl_3$) δ 7.94 (d, *J* = 7.4 Hz, 2H), 7.29 (d, *J* = 8.4 Hz, 2H), 6.96 – 6.94 (m, 2H), 6.88 – 6.84 (m, 1H), 6.60 (d, *J* = 6.4 Hz, 1H), 4.09 – 4.03 (m, 2H), 3.47 – 3.42 (m, 1H), 2.41 (s, 3H), 2.39 –2.33 (m, 1H), 2.07 (dd, *J* = 12.3, 4.8 Hz, 1H). ^{13}C NMR (126 MHz, $CDCl_3$) δ 150.22, 148.23, 143.52, 137.76 (d, *J* = 1.6 Hz), 136.14 (d, *J* = 2.8 Hz), 129.31, 128.61 (d, *J* = 10.5 Hz), 127.52 (d, *J* = 2.2 Hz), 124.92 (d, *J* = 6.6 Hz), 120.35 (d, *J* = 3.3 Hz), 116.29 (d, *J* = 20.3 Hz), 96.35, 66.43, 45.83, 33.60, 21.52. ^{19}F NMR (376 MHz, $CDCl_3$) δ -120.54. Enantiomeric excess: 87%, determined by HPLC (Chiralpak OJ-3, hexane/*i*-PrOH = 70/30; flow rate 0.8 ml/min; 25 ℃; 254 nm), first peak: t_R = 18.1 min, second peak: t_R = 38.4 min; HRMS (ESI) m/z calcd. for $C_{17}H_{16}FNNaO_3S$ $[M+Na]^+$ = 356.0727, found = 356.0718; IR spectrum (neat) (cm^{-1}) = 2876, 1597, 1348, 1258, 1165, 1094, 1074, 988, 961, 816, 779, 660, 596.

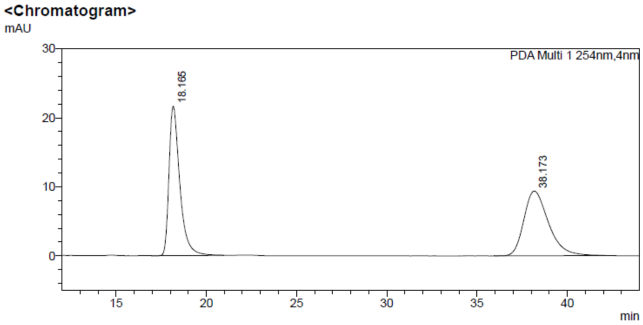

<Peak Table>
PDA Ch1 254nm

Peak#	Ret. Time	Height	Height%	Area	Area%
1	18.165	21634	69.761	898522	50.248
2	38.173	9377	30.239	889664	49.752
Total		31011	100.000	1788186	100.000

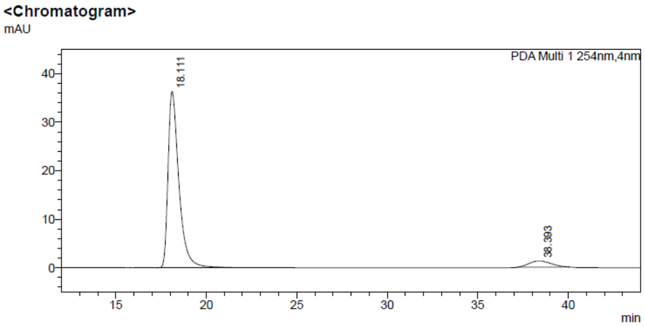

<Peak Table>
PDA Ch1 254nm

Peak#	Ret. Time	Height	Height%	Area	Area%
1	18.111	36312	96.555	1505511	93.244
2	38.393	1295	3.445	109079	6.756
Total		37608	100.000	1614590	100.000

(3a*R*,8a*R*)-5,6-difluoro-8-tosyl-3,3a,8,8a-tetrahydro-2H-furo[2,3-*b*]indole

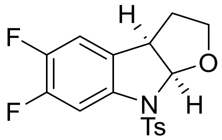

3pa; colorless solid (hexane/EtOAc = 8:1, 84% isolated yield); m.p. = 123-125 °C; $[\alpha]_D^{20}$ = 20.537 (*c* = 0.54, $CHCl_3$); ^{1}H NMR (500 MHz, $CDCl_3$) δ 7.83 (d, *J* = 8.3 Hz, 2H), 7.28 (d, *J* = 8.1 Hz, 2H), 7.25 – 7.23 (m, 1H), 6.95 – 6. 91 (m, 1H), 6.25 (d, *J* = 6.6 Hz, 1H), 3.99 (t, *J* = 8.0 Hz, 1H), 3.85 (t, *J* = 7.5 Hz, 1H), 3.33 – 3.28 (m, 1H), 2.40 (s, 3H), 2.32 – 2.24 (m, 1H), 1.96 (dd, *J* = 12.4, 4.6 Hz, 1H). ^{13}C NMR

(126 MHz, $CDCl_3$) δ 151.07 (d, *J* = 13.8 Hz), 149.10 (d, *J* = 13.8 Hz), 148.02 (d, *J* = 13.7 Hz), 146.08 (d, *J* = 13.7 Hz), 144.35, 137.51 (dd, *J* = 9.6, 2.3 Hz), 135.94, 129.73, 127.31, 126.90 (dd, *J* = 5.9, 3.4 Hz), 113.41 (d, *J* = 19.5 Hz), 102.84 (d, *J* = 23.8 Hz), 96.42, 66.39, 45.20, 33.55, 21.52. ^{19}F NMR (376 MHz, $CDCl_3$) δ -136.09 (d, *J* = 20.4 Hz), -143.62 (d, *J* = 20.3 Hz). Enantiomeric excess: 89%, determined by HPLC (Chiralpak OJ-3, hexane/*i*-PrOH = 70/30; flow rate 0.8 ml/min; 25 ℃; 254 nm), first peak: t_R = 16.1 min, second peak: t_R = 20.4 min; HRMS (ESI) m/z calcd. for $C_{17}H_{15}F_2NNaO_3S$ $[M+Na]^+$ = 374.0633, found = 374.0627; IR spectrum (neat) (cm^{-1}) = 2882, 1447, 1368, 1202, 1167, 1088, 961, 928, 881, 662, 610.

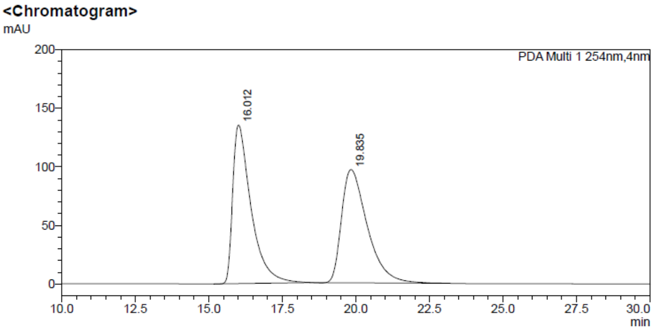

<Peak Table>
PDA Ch1 254nm

Peak#	Ret. Time	Area	Height	Conc.	Unit	Mark	Name
1	16.012	5861037	135252	0.000		M	
2	19.835	5881646	96692	0.000		M	
Total		11742684	231944				

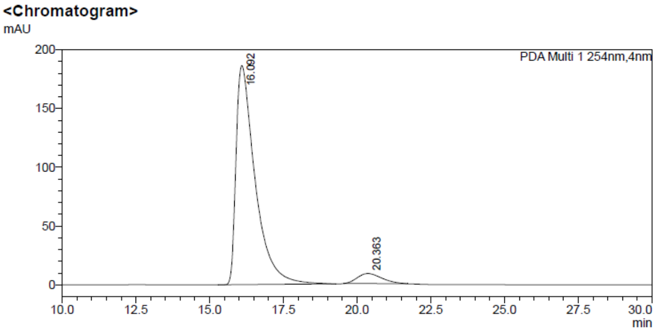

<Peak Table>
PDA Ch1 254nm

Peak#	Ret. Time	Height	Height%	Area	Area%
1	16.092	186423	95.625	8398780	94.454
2	20.363	8529	4.375	493108	5.546
Total		194953	100.000	8891888	100.000

(7a*R*,10a*R*)-7-tosyl-7a,9,10,10a-tetrahydro-7H-furo[3',2':4,5]pyrrolo[3,2-*f*]quinoxaline

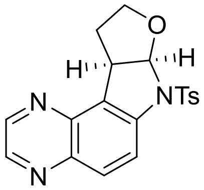

3qa; colorless solid (hexane/EtOAc = 2:1, 87% isolated yield); m.p. = 210-211 °C; $[\alpha]_D^{20}$ = 96.898 (*c* = 0.4, $CHCl_3$); ^{1}H NMR (500 MHz, $CDCl_3$) δ 8.75 (dd, *J* = 17.0, 1.8 Hz, 2H), 8.01 (q, *J* = 9.2 Hz, 2H), 7.89 (d, *J* = 8.4 Hz, 2H), 7.27 (d, *J* = 6.8 Hz, 2H), 6.47 (d, *J* = 6.8 Hz, 1H), 4.44 – 4.41 (m, 1H), 4.06 – 4.03 (m, 1H), 3.35 – 3.30 (m, 1H), 2.48 – 2.40 (m, 2H), 2.37 (s, 3H). ^{13}C NMR (126 MHz, $CDCl_3$) δ 145.04, 144.32, 143.22, 142.63, 140.62, 139.89, 136.29, 130.85, 129.78, 127.28, 125.12, 117.39, 97.04, 66.67, 44.51, 32.44, 21.51. Enantiomeric excess: 95%, determined by HPLC (Chiralpak OJ-3, hexane/*i*-PrOH = 60/40; flow rate 0.8 ml/min; 25 ℃; 254 nm), first peak: t_R = 24.2 min, second peak: t_R = 30.3 min; HRMS (ESI) m/z calcd. for $C_{19}H_{17}N_3NaO_3S$ $[M+Na]^+$ = 390.0883, found = 390.0881; IR spectrum (neat) (cm^{-1}) = 2884, 1362, 1348, 1258, 1161, 1080, 961, 947, 928, 881, 619, 588.

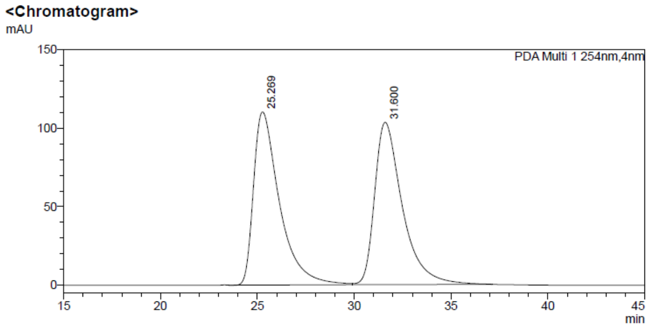

<Peak Table>
PDA Ch1 254nm

Peak#	Ret. Time	Height	Height%	Area	Area%
1	25.269	110288	51.608	10273054	49.891
2	31.600	103416	48.392	10317749	50.109
Total		213704	100.000	20590803	100.000

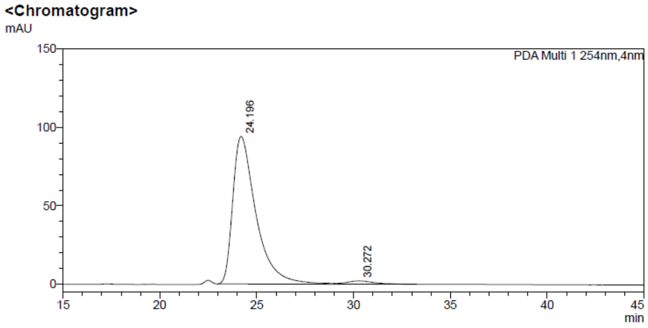

<Peak Table>
PDA Ch1 254nm

Peak#	Ret. Time	Height	Height%	Area	Area%
1	24.196	94077	97.830	7851282	97.414
2	30.272	2086	2.170	208385	2.586
Total		96164	100.000	8059667	100.000

(3a*R*,8a*R*)-8-(phenylsulfonyl)-3,3a,8,8a-tetrahydro-2H-furo[2,3-*b*]indole

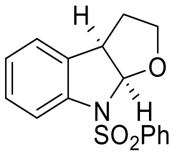

3ra; amorphous colorless solid (hexane/EtOAc = 8:1, 92% isolated yield); m.p. = 49-51 °C; $[\alpha]_D^{20}$ = 12.061 (c = 0.65, $CHCl_3$); ^1H NMR (500 MHz, $CDCl_3$) δ 7.99 (dd, J = 8.3, 1.0 Hz, 2H), 7.55 – 7.52 (m, 1H), 7.45 (dd, J = 10.6, 4.8 Hz, 2H), 7.38 (d, J = 8.1 Hz, 1H), 7.19 – 7.13 (m, 2H), 6.99 (td, J = 7.5, 0.7 Hz, 1H), 6.28 (d, J = 6.6 Hz, 1H), 3.96 (t, J = 8.0 Hz, 1H), 3.91 (t, J = 7.5 Hz, 1H), 3.32 – 3.26 (m, 1H), 2.33 – 2.25 (m, 1H), 2.02 (dd, J = 12.2, 4.7 Hz, 1H). ^{13}C NMR (126 MHz, $CDCl_3$) δ 141.36, 139.50, 132.96, 131.30, 128.89, 128.36, 127.27, 124.89, 123.58, 112.71, 95.74, 66.38, 45.49, 33.63. Enantiomeric excess: 95%, determined by HPLC (Chiralpak OJ-3, hexane/*i*-PrOH = 80/20; flow rate 0.8 ml/min; 25 °C; 254 nm), first peak: t_R = 18.8 min, second peak: t_R = 24.8 min; HRMS (ESI) m/z calcd. for $C_{16}H_{15}NNaO_3S$ $[M+Na]^+$ = 324.0665, found = 324.0661; IR spectrum (neat) (cm^{-1}) = 2884, 1362, 1169, 1080, 961, 881, 752, 592.

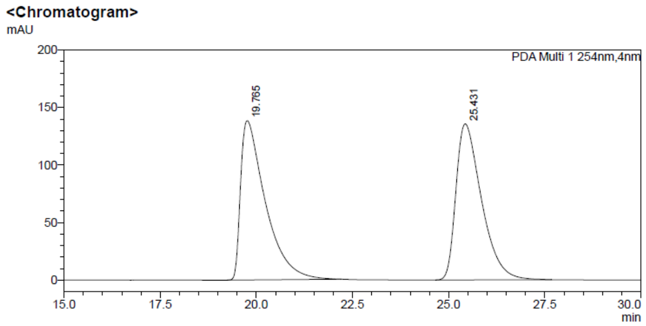

<Peak Table>
PDA Ch1 254nm

Peak#	Ret. Time	Height	Height%	Area	Area%
1	19.765	138514	50.510	6210755	49.757
2	25.431	135716	49.490	6271410	50.243
Total		274231	100.000	12482165	100.000

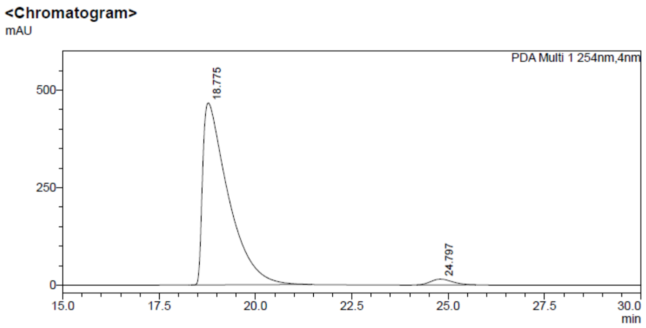

<Peak Table>
PDA Ch1 254nm

Peak#	Ret. Time	Height	Height%	Area	Area%
1	18.775	466490	96.937	21535263	97.416
2	24.797	14742	3.063	571214	2.584
Total		481232	100.000	22106477	100.000

(3a*S*,8a*R*)-2,3,3a,8a-tetrahydrofuro[2,3-*b*]benzofuran

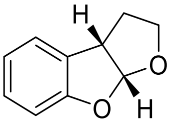

6aa; pale yellow oil (hexane/Et_2O = 20:1, 60% isolated yield); $[\alpha]_D^{20}$ = - 94.038 (*c* = 0.5, CH_2Cl_2); ^1H NMR (500 MHz, $CDCl_3$) δ 7.18 (d, *J* = 7.4 Hz, 1H), 7.14 (t, *J* = 7.7 Hz, 1H), 6.90 (td, *J* = 7.4, 0.7 Hz, 1H), 6.81 (d, *J* = 8.0 Hz, 1H), 6.31 (d, *J* = 5.7 Hz, 1H), 4.06 (t, *J* = 8.2 Hz, 1H), 4.00 (dd, *J* = 8.3, 5.9 Hz, 1H), 3.64 – 3.59 (m, 1H), 2.34 – 2.26 (m, 1H), 2.07 (dd, *J* = 12.2, 4.9 Hz, 1H). ^{13}C NMR (126 MHz, $CDCl_3$) δ 159.41, 128.66, 127.61, 124.67, 121.11, 110.85, 109.17, 67.18, 46.50, 33.54. Enantiomeric excess: 96%, determined by HPLC (Chiralpak IC, hexane/*i*-PrOH = 98/2; flow rate 1.0 ml/min; 25 ℃; 210 nm), first peak: t_R = 10.3 min, second peak: t_R = 13.0 min; HRMS (ESI) m/z calcd. for $C_{10}H_{10}NaO_2$ $[M+Na]^+$ = 185.0573, found = 185.0589; IR spectrum (neat) (cm^{-1}) = 2974, 1198, 1166, 1083, 961, 928, 882, 779, 733, 669.

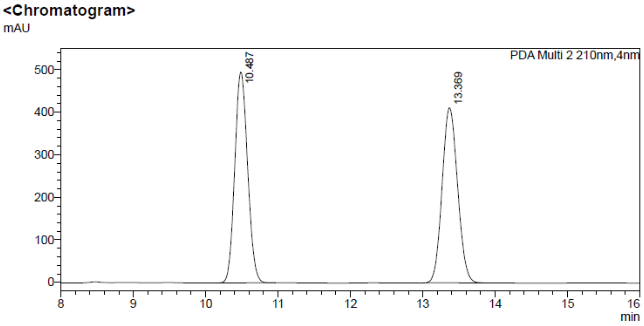

<Peak Table>
PDA Ch2 210nm

Peak#	Ret. Time	Height	Height%	Area	Area%
1	10.487	496138	54.646	6114368	49.980
2	13.369	411779	45.354	6119199	50.020
Total		907917	100.000	12233567	100.000

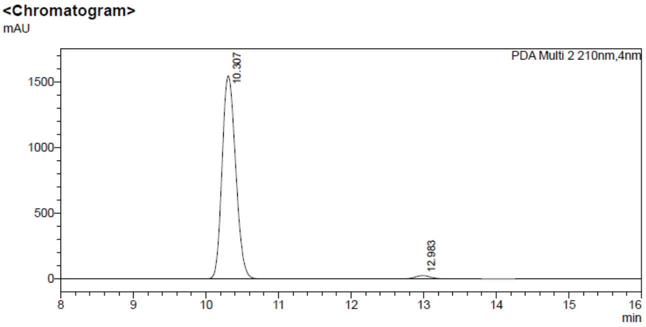

<Peak Table>
PDA Ch2 210nm

Peak#	Ret. Time	Height	Height%	Area	Area%
1	10.307	1546570	98.380	20040363	98.157
2	12.983	25463	1.620	376269	1.843
Total		1572032	100.000	20416631	100.000

(3a*S*,8a*R*)-5-fluoro-2,3,3a,8a-tetrahydrofuro[2,3-*b*]benzofuran

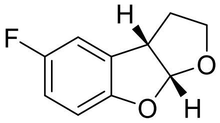

6ba; pale yellow oil (hexane/Et_2O = 20:1, 77% isolated yield); $[\alpha]_D^{20}$ = - 149.872 (*c* = 0.4, CH_2Cl_2); ^1H NMR (500 MHz, $CDCl_3$) δ 6.90 – 6.88 (m, 1H), 6.83 (td, *J* = 8.9, 2.7 Hz, 1H), 6.71 (dd, *J* = 8.7, 4.2 Hz, 1H), 6.32 (d, *J* = 5.7 Hz, 1H), 4.08 (t, *J* = 8.2 Hz, 1H), 4.00 (dd, *J* = 8.2, 6.0 Hz, 1H), 3.65 – 6.60 (m, 1H), 2.34 – 2.26 (m, 1H), 2.05 (dd, *J* = 12.3, 4.9 Hz, 1H). ^{13}C NMR (126 MHz, $CDCl_3$) δ 157.81 (d, J = 237.6 Hz), 155.34 (d, *J* = 1.4 Hz), 128.89 (d, *J* = 8.5 Hz), 114.94 (d, *J* = 24.1 Hz), 111.63 (d, *J* = 24.7 Hz), 111.47, 109.41 (d, *J* = 8.5 Hz), 67.21, 46.86 (d, *J* = 1.7 Hz), 33.40. ^{19}F NMR (376 MHz, $CDCl_3$) δ -123.52. Enantiomeric excess: 98%, determined by HPLC (Chiralpak OD-H, hexane/*i*-PrOH = 98/2; flow rate 1.0 ml/min; 25 ℃; 210 nm), first peak: t_R = 8.5 min, second peak: t_R = 9.6 min; HRMS (ESI) m/z calcd. for

$C_{10}H_9FNaO_2$ $[M+Na]^+$ = 203.0479, found = 203.0493; IR spectrum (neat) (cm^{-1}) = 2986, 1447, 1234, 1190, 1165, 1126, 1097, 1072, 960, 926, 856, 799, 740, 715, 573.

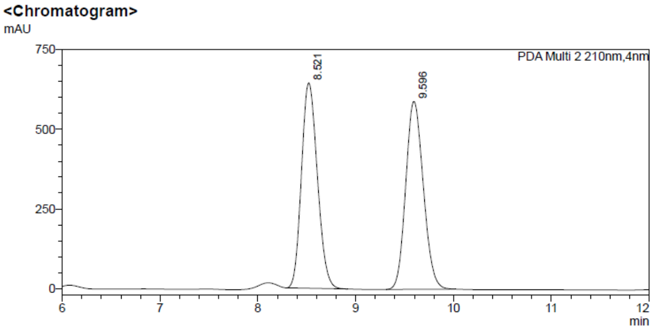

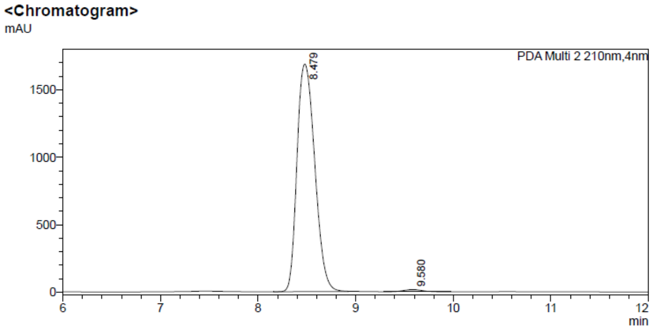

<Peak Table>
PDA Ch2 210nm

Peak#	Ret. Time	Height	Height%	Area	Area%
1	8.521	645091	52.258	7295914	49.702
2	9.596	589354	47.742	7383476	50.298
Total		1234446	100.000	14679390	100.000

<Peak Table>
PDA Ch2 210nm

Peak#	Ret. Time	Height	Height%	Area	Area%
1	8.479	1687466	98.992	21383499	99.016
2	9.580	17181	1.008	212560	0.984
Total		1704647	100.000	21596059	100.000

(3a*S*,8a*R*)-5-methyl-2,3,3a,8a-tetrahydrofuro[2,3-*b*]benzofuran

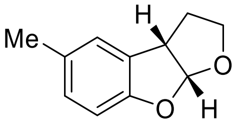

6ca; pale yellow oil (hexane/Et_2O = 20:1, 53% isolated yield); $[\alpha]_D^{20}$ = - 168.117 (c = 0.5, CH_2Cl_2); ^1H NMR (500 MHz, $CDCl_3$) δ 6.98 (s, 1H), 6.93 (dd, J = 8.1, 0.6 Hz, 1H), 6.69 (d, J = 8.1 Hz, 1H), 6.27 (d, J = 5.7 Hz, 1H), 4.05 (t, J = 8.1 Hz, 1H), 3.95 (dd, J = 8.3, 5.9 Hz, 1H), 3.63 – 3.58 (m, 1H), 2.31 – 2.23 (m, 1H), 2.28 (s, 3H), 2.06 – 2.03 (m, 1H). ^{13}C NMR (126 MHz, $CDCl_3$) δ 157.31, 130.37, 129.01, 127.51, 125.14, 110.91, 108.65, 67.13, 46.54, 33.48, 20.73. Enantiomeric excess: 95%, determined by HPLC (Chiralpak OD-H, hexane/*i*-PrOH = 98/2; flow rate 1.0 ml/min; 25 ℃; 210 nm), first peak: t_R = 8.0 min, second peak: t_R = 8.4 min; HRMS (ESI) m/z calcd. for $C_{11}H_{12}NaO_2$ $[M+Na]^+$ = 199.0730, found = 199.0732; IR spectrum (neat) (cm^{-1}) = 2976, 1458, 1448, 1307, 1246, 1202, 1072, 1022, 957, 831, 808, 745, 654.

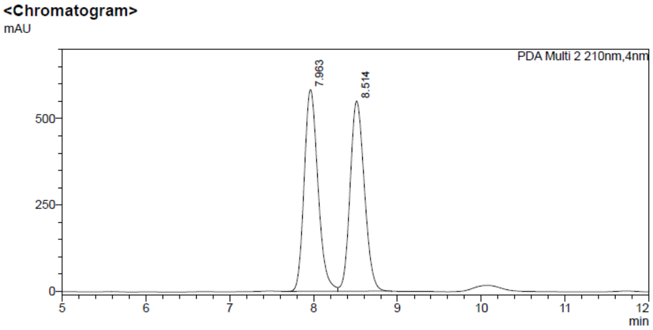

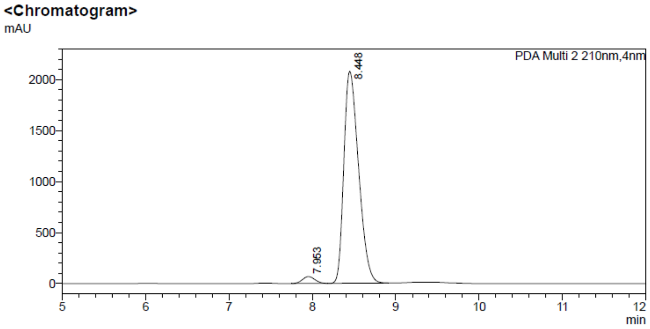

<Peak Table>
PDA Ch2 210nm

Peak#	Ret. Time	Height	Height%	Area	Area%
1	7.963	582522	51.435	6681623	50.642
2	8.514	550017	48.565	6512179	49.358
Total		1132538	100.000	13193802	100.000

<Peak Table>
PDA Ch2 210nm

Peak#	Ret. Time	Height	Height%	Area	Area%
1	7.953	67264	3.131	695921	2.657
2	8.448	2080703	96.869	25494759	97.343
Total		2147966	100.000	26190679	100.000

(3a*S*,8a*R*)-5-methyl-2,3,3a,8a-tetrahydrofuro[2,3-*b*]benzofuran

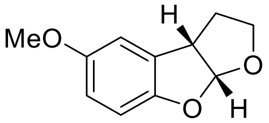

6da; pale yellow oil (hexane/Et_2O = 10:1, 72% isolated yield); $[\alpha]_D^{20}$ = - 182.367 (*c* = 0.54, CH_2Cl_2); ^1H NMR (500 MHz, $CDCl_3$) δ 6.78 (d, *J* = 2.4 Hz, 1H), 6.73 – 6.69 (m, 2H), 6.29 (d, *J* = 5.7 Hz, 1H), 4.07 (t, *J* = 8.1 Hz, 1H), 3.99 (dd, *J* = 8.4, 5.8 Hz, 1H), 3.77 (s, 3H), 3.66 – 3.61 (m, 1H), 2.34 – 2.25 (m, 1H), 2.10 – 2.06 (m, 1H). ^{13}C NMR (126 MHz, $CDCl_3$) δ 154.52, 153.44, 128.44, 113.53, 111.07, 110.70, 109.08, 67.12, 55.89, 46.96, 33.39. Enantiomeric excess: 98%, determined by HPLC (Chiralpak IC, hexane/*i*-PrOH = 98/2; flow rate 1.0 ml/min; 25 ℃; 210 nm), first peak: t_R = 20.3 min, second peak: t_R = 24.8 min; HRMS (ESI) m/z calcd. for $C_{11}H_{12}NaO_3$ [M+Na]$^+$ = 215.0679, found = 215.0676; IR spectrum (neat) (cm^{-1}) = 2980, 1240, 1198, 1076, 1068, 959, 928, 810, 739, 656.

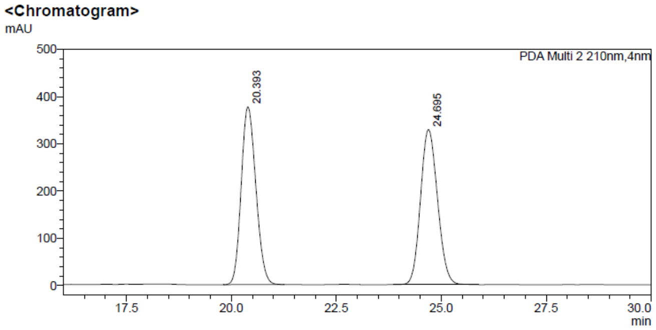

<Peak Table>

PDA Ch2 210nm

Peak#	Ret. Time	Height	Height%	Area	Area%
1	20.393	376296	53.411	9163463	49.760
2	24.695	328227	46.589	9251996	50.240
Total		704523	100.000	18415460	100.000

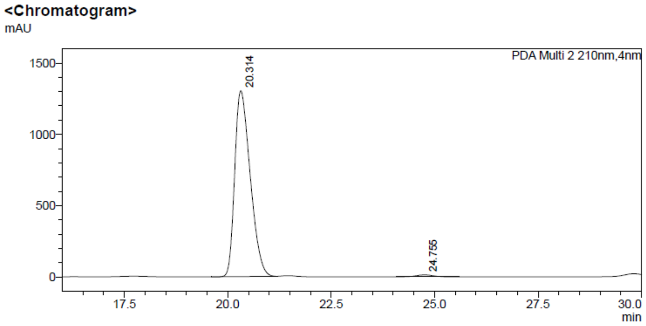

<Peak Table>

PDA Ch2 210nm

Peak#	Ret. Time	Height	Height%	Area	Area%
1	20.314	1302541	98.976	33926047	98.934
2	24.755	13481	1.024	365668	1.066
Total		1316023	100.000	34291715	100.000

(3a*S*,8a*R*)-5-methyl-2,3,3a,8a-tetrahydrofuro[2,3-*b*]benzofuran

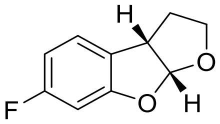

6ea; pale yellow oil (hexane/Et_2O = 20:1, 51% isolated yield); $[\alpha]_D^{20}$ = - 138.84 (*c* = 0.25, CH_2Cl_2); ^1H NMR (500 MHz, $CDCl_3$) δ 7.09 (dd, *J* = 7.8, 6.1 Hz, 1H), 6.62 – 6.58 (m, 1H), 6.52 (dd, *J* = 9.4, 2.3 Hz, 1H), 6.34 (d, *J* = 5.7 Hz, 1H), 4.08 (t, *J* = 8.2 Hz, 1H), 3.97 – 3.94 (m, 1H), 3.65 – 3.60 (m, 1H), 2.32 – 2.24 (m, 1H), 2.03 (dd, *J* = 12.2, 4.8 Hz, 1H). ^{13}C NMR (126 MHz, $CDCl_3$) δ 163.33 (d, J = 244.2 Hz), 160.45 (d, *J* = 13.1 Hz), 124.94 (d, *J* = 10.5 Hz), 123.28 (d, *J* = 2.6 Hz), 112.17, 107.76 (d, *J* = 22.8 Hz), 97.61 (d, *J* = 26.5 Hz), 67.28, 45.88, 33.59. ^{19}F NMR (376 MHz, $CDCl_3$) δ -113.10. Enantiomeric excess: 90%, determined by HPLC (Chiralpak OD-H, hexane/*i*-PrOH = 98/2; flow rate 1.0 ml/min; 25 ℃; 210 nm), first peak: t_R = 6.6 min, second peak: t_R = 7.7 min; HRMS (ESI) m/z calcd. for $C_{10}H_9FNaO_2$ [M+Na]$^+$

= 203.0479, found = 203.0488; IR spectrum (neat) (cm^{-1}) = 2984, 1610, 1439, 1325, 1256, 1132, 1074, 957, 918, 837, 800, 752, 610.

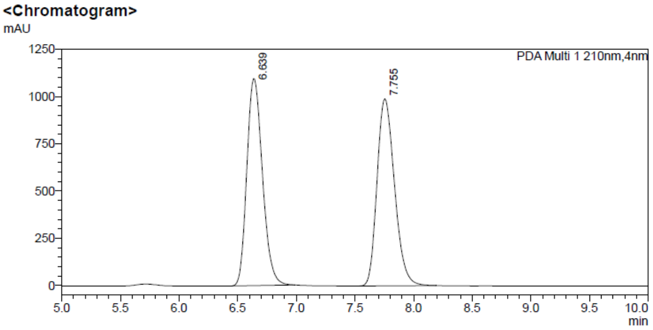

<Peak Table>
PDA Ch1 210nm

Peak#	Ret. Time	Height	Height%	Area	Area%
1	6.639	1096092	52.534	10150554	49.725
2	7.755	990355	47.466	10262639	50.275
Total		2086447	100.000	20413193	100.000

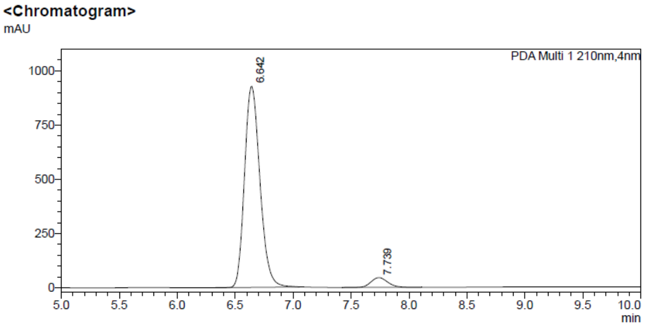

<Peak Table>
PDA Ch1 210nm

Peak#	Ret. Time	Height	Height%	Area	Area%
1	6.642	927751	95.389	8590942	95.088
2	7.739	44849	4.611	443829	4.912
Total		972600	100.000	9034771	100.000

(3a*S*,8a*R*)-6-methyl-2,3,3a,8a-tetrahydrofuro[2,3-*b*]benzofuran

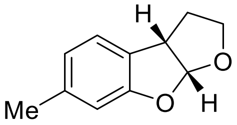

6fa; pale yellow oil (hexane/Et_2O = 20:1, 58% isolated yield); $[\alpha]_D^{20}$ = - 125.319 (*c* = 0.25, CH_2Cl_2); ^1H NMR (500 MHz, $CDCl_3$) δ 7.05 (d, *J* = 7.5 Hz, 1H), 6.72 (dd, *J* = 7.5, 0.5 Hz, 1H), 6.63 (s, 1H), 6.29 (d, *J* = 5.7 Hz, 1H), 4.05 (t, *J* = 8.1 Hz, 1H), 3.95 (dd, *J* = 7.8, 6.2 Hz, 1H), 3.63 – 3.58 (m, 1H), 2.30 (s, 3H), 2.29 – 2.22 (m, 1H), 2.03 (dd, *J* = 12.1, 4.8 Hz, 1H). ^{13}C NMR (126 MHz, $CDCl_3$) δ 159.63, 138.91, 124.61, 124.20, 121.83, 111.13, 109.82, 67.15, 46.24, 33.58, 21.47. Enantiomeric excess: 92%, determined by HPLC (Chiralpak IC, hexane/*i*-PrOH = 98/2; flow rate 1.0 ml/min; 25 ℃; 210 nm), first peak: t_R = 10.2 min, second peak: t_R = 13.8 min; HRMS (ESI) m/z calcd. for $C_{11}H_{12}NaO_2$ $[M+Na]^+$ = 199.0730, found = 199.0725; IR spectrum (neat) (cm^{-1}) = 2978, 1591, 1445, 1321, 1252, 1072, 943, 922, 800, 750, 627, 590.

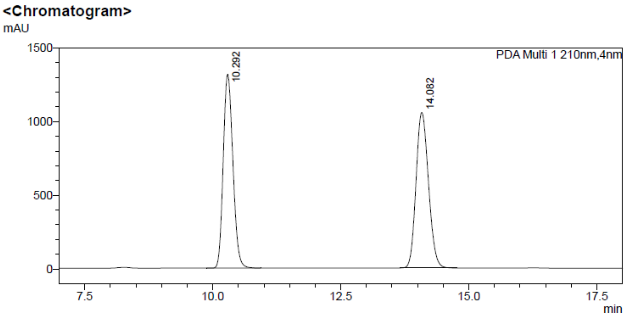

<Peak Table>
PDA Ch1 210nm

Peak#	Ret. Time	Height	Height%	Area	Area%
1	10.292	1316326	55.482	17256308	49.090
2	14.082	1056200	44.518	17895920	50.910
Total		2372526	100.000	35152229	100.000

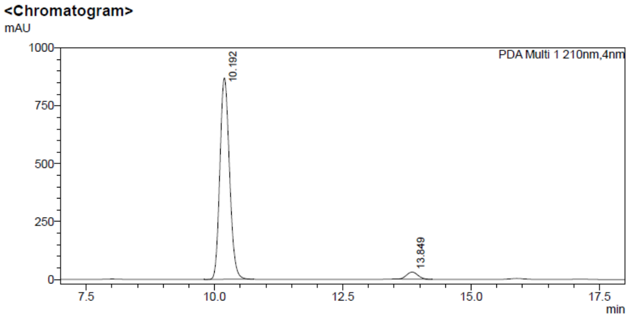

<Peak Table>
PDA Ch1 210nm

Peak#	Ret. Time	Height	Height%	Area	Area%
1	10.192	870227	96.607	11367356	96.037
2	13.849	30567	3.393	469106	3.963
Total		900793	100.000	11836462	100.000

(3a*S*,8a*R*)-7-fluoro-2,3,3a,8a-tetrahydrofuro[2,3-*b*]benzofuran

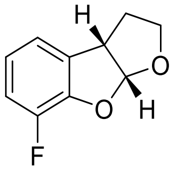

6ga; pale yellow oil (hexane/Et_2O = 20:1, 64% isolated yield); $[\alpha]_D^{20}$ = - 91.870 (*c* = 0.4, CH_2Cl_2); ^{1}H NMR (500 MHz, $CDCl_3$) δ 6.97 – 6.91 (m, 2H), 6.85 – 6.81 (m, 1H), 6.39 (d, *J* = 5.6 Hz, 1H), 4.10 (t, *J* = 8.2 Hz, 1H), 4.05 (dd, *J* = 8.5, 5.7 Hz, 1H), 3.68 – 3.63 (m, 1H), 2.35 – 2.27 (m, 1H), 2.08 (dd, *J* = 12.3, 4.9 Hz, 1H). ^{13}C NMR (126 MHz, $CDCl_3$) δ 146.51 (d, J = 246.4 Hz), 146.04 (d, *J* = 10.5 Hz), 131.23 (d, *J* = 3.0 Hz), 121.61 (d, *J* = 5.6 Hz), 119.95 (d, *J* = 3.5 Hz), 115.75 (d, *J* = 16.9 Hz), 112.19, 67.41, 46.95 (d, *J* = 2.0 Hz), 33.39. ^{19}F NMR (376 MHz, $CDCl_3$) δ -137.96. Enantiomeric excess: 98%, determined by HPLC (Chiralpak OD-H, hexane/*i*-PrOH = 98/2; flow rate 1.0 ml/min; 25 °C; 210 nm), first peak: t_R = 7.7 min, second peak: t_R = 9.3 min; HRMS (ESI) m/z calcd. for $C_{10}H_9FNaO_2$ $[M+Na]^+$ = 203.0479, found = 203.0482; IR spectrum (neat) (cm^{-1}) = 2989, 1599, 1470, 1323, 1260, 1176, 1074, 943, 924, 814, 773, 731, 696, 642.

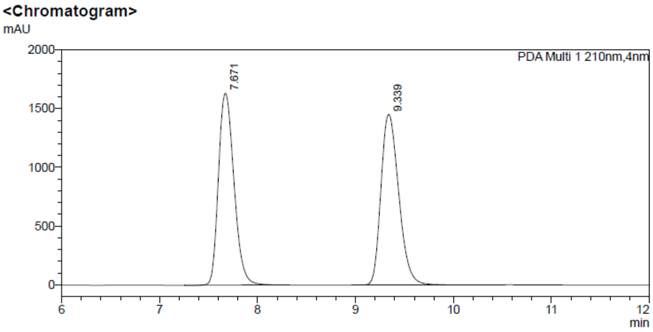

<Peak Table>
PDA Ch1 210nm

Peak#	Ret. Time	Height	Height%	Area	Area%
1	7.671	1634252	52.975	17970548	49.566
2	9.339	1450724	47.025	18285398	50.434
Total		3084976	100.000	36255946	100.000

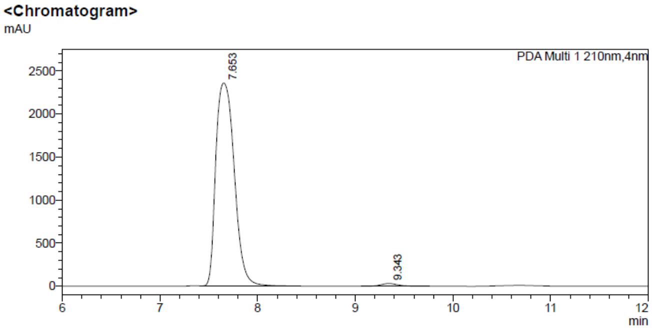

<Peak Table>
PDA Ch1 210nm

Peak#	Ret. Time	Height	Height%	Area	Area%
1	7.653	2356338	98.678	31036614	98.809
2	9.343	31563	1.322	374187	1.191
Total		2387901	100.000	31410801	100.000

(3a*S*,8a*R*)-7-methoxy-2,3,3a,8a-tetrahydrofuro[2,3-*b*]benzofuran

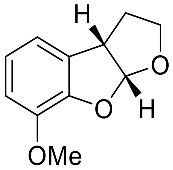

6ha; pale yellow oil (hexane/Et_2O = 10:1, 61% isolated yield); $[\alpha]_D^{20}$ = - 113.542 (*c* = 0.625, CH_2Cl_2); ^{1}H NMR (500 MHz, $CDCl_3$) δ 6.88 – 6.85 (m, 1H), 6.81 – 6.80 (m, 1H), 6.76 (d, *J* = 8.0 Hz, 1H), 6.35 (d, *J* = 5.7 Hz, 1H), 4.08 – 4.00 (m, 2H), 3.87 (s, 3H), 3.66 – 3.61 (m, 1H), 2.32 – 2.26 (m, 1H), 2.06 (dd, *J* = 12.2, 4.9 Hz, 1H). ^{13}C NMR (126 MHz, $CDCl_3$) δ 147.71, 143.64, 128.63, 121.66, 116.61, 111.63, 111.35, 67.19, 55.88, 47.01, 33.28. Enantiomeric excess: 99%, determined by HPLC (Chiralpak OD-H,

hexane/*i*-PrOH = 98/2; flow rate 1.0 ml/min; 25 ℃; 220 nm), first peak: t_R = 20.8 min, second peak: t_R = 30.0 min; HRMS (ESI) m/z calcd. for $C_{11}H_{12}NaO_3$ $[M+Na]^+$ = 215.0679, found = 215.0680; IR spectrum (neat) (cm^{-1}) = 2982, 1618, 1593, 1460, 1302, 1271, 1198, 1060, 939, 771, 731, 648.

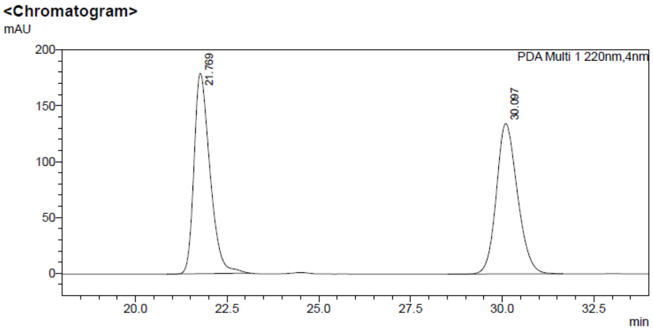

<Peak Table>
PDA Ch1 220nm

Peak#	Ret. Time	Height	Height%	Area	Area%
1	21.769	179503	57.141	5428123	50.194
2	30.097	134638	42.859	5386096	49.806
Total		314141	100.000	10814219	100.000

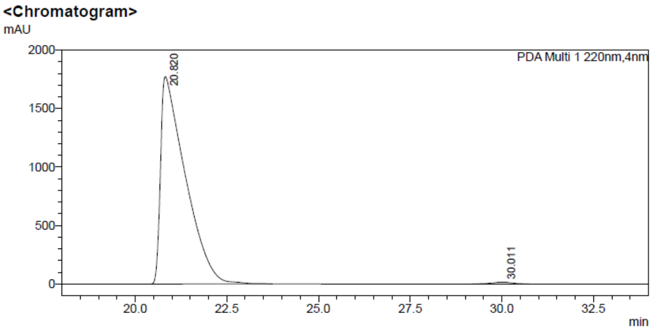

<Peak Table>
PDA Ch1 220nm

Peak#	Ret. Time	Height	Height%	Area	Area%
1	20.820	1774718	99.153	83111894	99.259
2	30.011	15157	0.847	620850	0.741
Total		1789875	100.000	83732744	100.000

(2*R*,3a*R*,8a*R*)-2-(p-tolyl)-8-tosyl-3,3a,8,8a-tetrahydro-2H-furo[2,3-b]indole

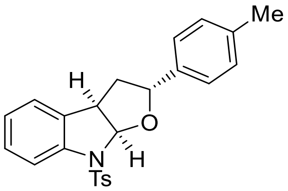

3ab; colorless solid (hexane/EtOAc = 7:1, 52% isolated yield); m.p. = 161-163 °C; $[\alpha]_D^{20}$ = 13.927 (*c* = 0.55, CH_2Cl_2); ^1H NMR (500 MHz, $CDCl_3$) δ 7.89 (d, *J* = 8.3 Hz, 2H), 7.40 (d, *J* = 8.1 Hz, 1H), 7.21 – 7.15 (m, 4H), 7.12 (s, 4H), 7.01 (td, *J* = 7.5, 0.7 Hz, 1H), 6.49 (d, *J* = 6.6 Hz, 1H), 4.42 (dd, *J* = 11.2, 4.4 Hz, 1H), 4.04 (t, *J* = 7.4 Hz, 1H), 2.33 (s, 3H), 2.33 (s, 3H), 2.30 (d, *J* = 4.5 Hz, 1H), 2.24 – 2.18 (m, 1H). ^{13}C NMR (126 MHz, $CDCl_3$) δ 143.73, 141.62, 137.59, 136.65, 136.17, 131.44, 129.43, 128.93, 128.41, 127.52, 126.11, 124.85, 123.42, 112.64, 95.46, 79.18, 46.24, 42.13, 21.42, 21.09. Enantiomeric excess: 85%, determined by HPLC (Chiralpak AD-H, hexane/*i*-PrOH = 70/30; flow rate 0.8 ml/min; 25 ℃; 254 nm), first peak: t_R = 11.4 min, second peak: t_R = 14.7 min; HRMS (ESI) m/z calcd. for $C_{24}H_{23}NNaO_3S$ $[M+Na]^+$ = 428.1291, found = 428.1302; IR spectrum (neat) (cm^{-1}) = 2884, 1614, 1447, 1354, 1252, 1167, 1074, 961, 928, 814, 768, 733, 664.

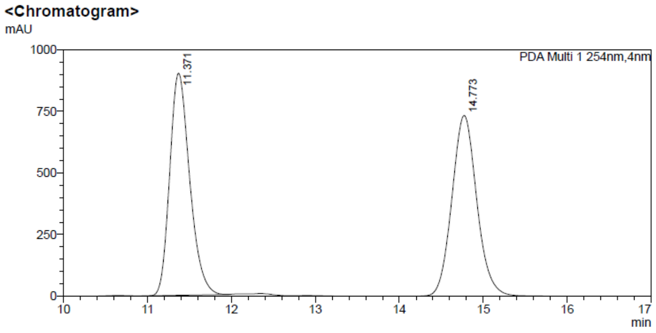

<Peak Table>

PDA Ch1 254nm

Peak#	Ret. Time	Height	Height%	Area	Area%
1	11.371	902520	55.168	14910639	49.724
2	14.773	733425	44.832	15076105	50.276
Total		1635946	100.000	29986744	100.000

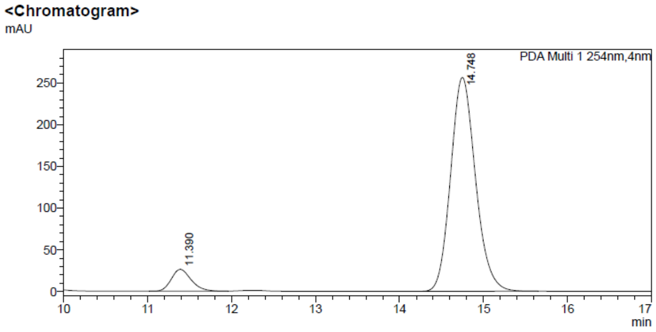

<Peak Table>

PDA Ch1 254nm

Peak#	Ret. Time	Height	Height%	Area	Area%
1	11.390	26228	9.284	431140	7.545
2	14.748	256296	90.716	5282751	92.455
Total		282524	100.000	5713892	100.000

methyl 4-((2*R*,3a*R*,8a*R*)-8-tosyl-3,3a,8,8a-tetrahydro-2H-furo[2,3-b]indol-2-yl)benzoate

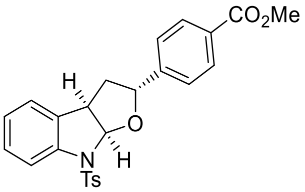

3ac; colorless solid (hexane/EtOAc = 4:1, 48% isolated yield); m.p. = 166-168 °C; $[\alpha]_D^{20}$ = 5.673 (*c* = 0.55, CH_2Cl_2); ^1H NMR (500 MHz, $CDCl_3$) δ 7.99 (d, *J* = 8.3 Hz, 2H), 7.87 (d, *J* = 8.3 Hz, 2H), 7.43 (d, *J* = 8.1 Hz, 1H), 7.33 (d, *J* = 8.2 Hz, 2H), 7.22 (t, *J* = 7.8 Hz, 1H), 7.17 (t, *J* = 6.8 Hz, 3H), 7.03 (dd, *J* = 7.5, 7.0 Hz, 1H), 6.51 (d, *J* = 6.6 Hz, 1H), 4.50 (dd, *J* = 11.3, 4.4 Hz, 1H), 4.07 (t, *J* = 7.4 Hz, 1H), 3.91 (s, 3H), 2.40 (dd, *J* = 12.3, 4.5 Hz, 1H), 2.32 (s, 3H), 2.21 – 2.15 (m, 1H). ^{13}C NMR (126 MHz, $CDCl_3$) δ 166.74, 144.63, 143.89, 141.56, 136.49, 131.08, 129.58, 129.45, 128.59, 127.41, 125.81, 124.86, 123.61, 112.83, 95.57, 78.69, 52.03, 46.25, 42.22, 21.40. Enantiomeric excess: 90%, determined by HPLC (Chiralpak OJ-H, hexane/*i*-PrOH = 70/30; flow rate 0.8 ml/min; 25 ℃; 254 nm), first peak: t_R = 38.0 min, second peak: t_R = 52.3 min; HRMS (ESI) m/z calcd. for $C_{25}H_{23}NNaO_5S$ $[M+Na]^+$ = 472.1189, found = 472.1199; IR spectrum (neat) (cm^{-1}) = 2884, 1612, 1277, 1250, 1198, 1082, 1067, 959, 930, 815, 733, 665.

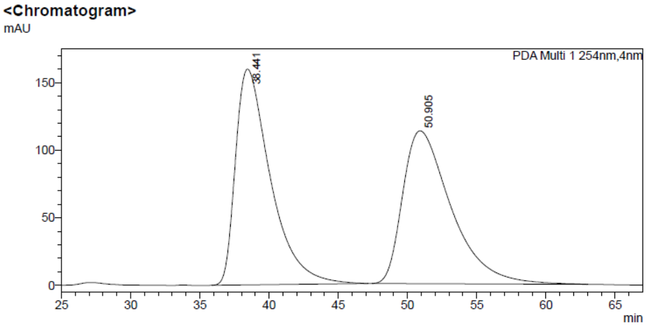

<Peak Table>
PDA Ch1 254nm

Peak#	Ret. Time	Height	Height%	Area	Area%
1	38.441	159652	58.528	28106010	50.432
2	50.905	113125	41.472	27624527	49.568
Total		272777	100.000	55730537	100.000

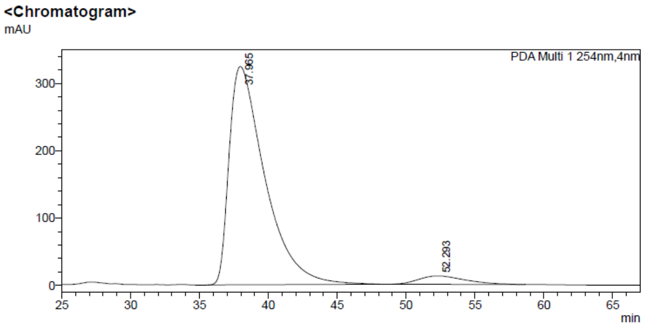

<Peak Table>
PDA Ch1 254nm

Peak#	Ret. Time	Height	Height%	Area	Area%
1	37.965	324275	96.307	58554747	95.248
2	52.293	12434	3.693	2921649	4.752
Total		336709	100.000	61476396	100.000

(2*R*,3a*R*,8a*R*)-2-(benzofuran-5-yl)-8-tosyl-3,3a,8,8a-tetrahydro-2H-furo[2,3-b]indole

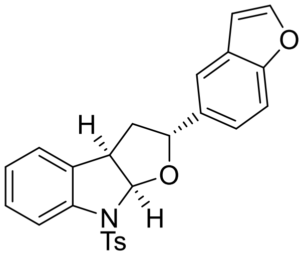

3ad; pale yellow solid (hexane/EtOAc = 7:1, 87% isolated yield); m.p. = 59-60 °C; $[\alpha]_D^{20}$ = 12.613 (*c* = 0.463, CH_2Cl_2); ^{1}H NMR (500 MHz, $CDCl_3$) δ 7.89 (d, *J* = 8.3 Hz, 2H), 7.60 (d, *J* = 2.2 Hz, 1H), 7.49 (d, *J* = 1.5 Hz, 1H), 7.43 – 7.41 (m, 2H), 7.21 – 7.13 (m, 5H), 7.06 – 7.01 (m, 1H), 6.72 (dd, *J* = 2.1, 0.8 Hz, 1H), 6.53 (d, *J* = 6.6 Hz, 1H), 4.55 (dd, *J* = 11.2, 4.4 Hz, 1H), 4.07 (t, *J* = 7.4 Hz, 1H), 2.37 (dd, *J* = 12.4, 4.5 Hz, 1H), 2.32 (s, 3H), 2.29 – 2.22 (m, 1H). ^{13}C NMR (126 MHz, $CDCl_3$) δ 154.57, 145.41, 143.76, 141.64, 136.63, 133.77, 131.44, 129.44, 128.45, 127.49, 124.88, 123.48, 122.58, 121.52, 118.88, 112.70, 111.07, 106.51, 95.47, 79.48, 46.29, 42.61, 21.40. Enantiomeric excess: 86%, determined by HPLC (Chiralpak OJ-H, hexane/*i*-PrOH = 70/30; flow rate 0.8 ml/min; 25 ℃; 254 nm), first peak: t_R = 16.5 min, second peak: t_R = 23.9 min; HRMS (ESI) m/z calcd. for $C_{25}H_{21}NNaO_4S$ $[M+Na]^+$ = 454.1083, found = 454.1087; IR spectrum (neat) (cm^{-1}) = 2884, 1481, 1352, 1167, 1092, 1074, 1005, 961, 949, 814, 743, 662.

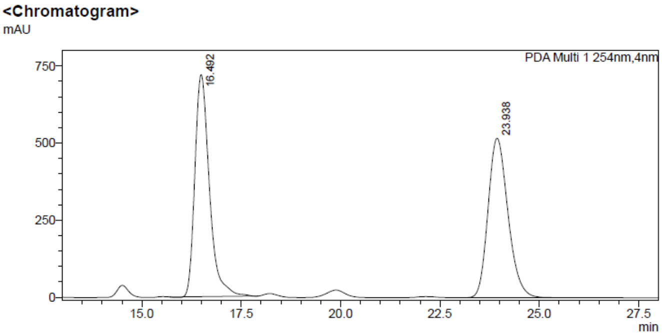

<Peak Table>
PDA Ch1 254nm

Peak#	Ret. Time	Height	Height%	Area	Area%
1	16.492	719981	58.210	18302204	50.378
2	23.938	516892	41.790	18027435	49.622
Total		1236874	100.000	36329639	100.000

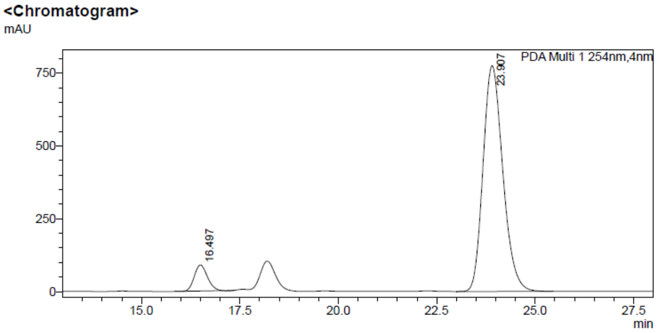

<Peak Table>
PDA Ch1 254nm

Peak#	Ret. Time	Height	Height%	Area	Area%
1	16.497	89702	10.371	2079435	7.120
2	23.907	775251	89.629	27126303	92.880
Total		864953	100.000	29205738	100.000

(2*R*,3a*S*,8a*R*)-2-(*p*-tolyl)-2,3,3a,8a-tetrahydrofuro[2,3-*b*]benzofuran

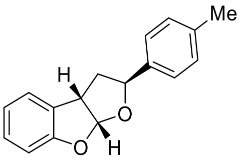

6ab; pale yellow oil (hexane/Et_2O = 20:1, 78% isolated yield); $[\alpha]_D^{20}$ = - 54.179 (*c* = 0.5, CH_2Cl_2); ^1H NMR (500 MHz, $CDCl_3$) δ 7.23 – 7.20 (m, 3H), 7.18 (d, *J* = 7.7 Hz, 1H), 7.13 (d, *J* = 8.0 Hz, 2H), 6.94 (td, *J* = 7.4, 0.7 Hz, 1H), 6.86 (d, *J* = 8.0 Hz, 1H), 6.47 (d, *J* = 5.8 Hz, 1H), 4.86 (dd, *J* = 11.3, 4.6 Hz, 1H), 4.14 (dd, *J* = 8.0, 6.1 Hz, 1H), 2.40 (dd, *J* = 12.4, 4.6 Hz, 1H), 2.33 (s, 3H), 2.25 – 2.19 (m, 1H). ^{13}C NMR (126 MHz, $CDCl_3$) δ 159.56, 137.60, 136.50, 129.05, 128.79, 127.82, 126.00, 124.70, 121.19, 110.51, 109.35, 80.04, 47.36, 42.09, 21.11. Enantiomeric excess: 95%, determined by HPLC (Chiralpak IF, hexane/*i*-PrOH = 95/5; flow rate 0.8 ml/min; 25 ℃; 220 nm), first peak: t_R = 10.2 min, second peak: t_R = 11.0 min; HRMS (ESI) m/z calcd. for $C_{17}H_{16}NaO_2$ $[M+Na]^+$ = 275.1043, found = 275.1050; IR spectrum (neat) (cm^{-1}) = 2982, 1597, 1477, 1460, 1323, 1246, 1223, 1180, 1098, 1072, 995, 981, 912, 889, 812, 748, 588.

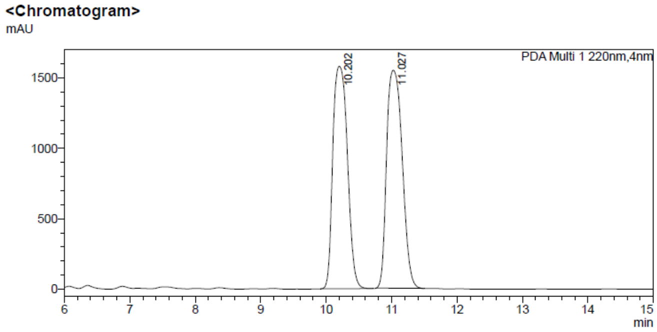

<Peak Table>
PDA Ch1 220nm

Peak#	Ret. Time	Height	Height%	Area	Area%
1	10.202	1577943	50.496	24497949	48.805
2	11.027	1546914	49.504	25697177	51.195
Total		3124857	100.000	50195126	100.000

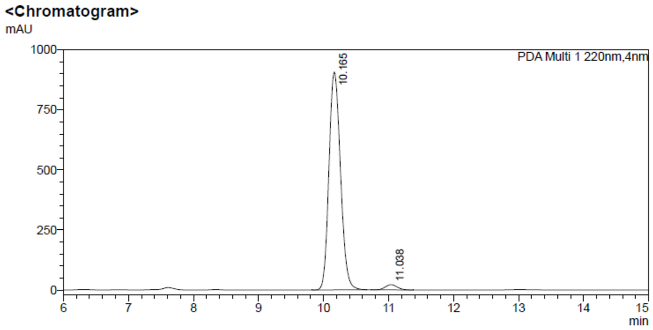

<Peak Table>
PDA Ch1 220nm

Peak#	Ret. Time	Height	Height%	Area	Area%
1	10.165	907498	97.655	11181586	97.584
2	11.038	21789	2.345	276862	2.416
Total		929287	100.000	11458449	100.000

methyl 4-((2*R*,3a*S*,8a*R*)-2,3,3a,8a-tetrahydrofuro[2,3-*b*]benzofuran-2-yl)benzoate

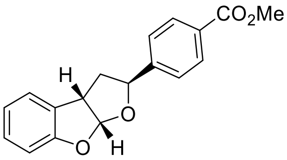

6ac; colorless solid (hexane/Et_2O = 10:1, 45% isolated yield); m.p. = 131-132 °C; $[\alpha]_D^{20}$ = - 15.2 (*c* = 0.35, CH_2Cl_2); ^1H NMR (500 MHz, $CDCl_3$) δ 8.00 (d, *J* = 8.4 Hz, 2H), 7.40 (d, *J* = 8.2 Hz, 2H), 7.26 – 7.19 (m, 2H), 6.96 (td, *J* = 7.5, 0.8 Hz, 1H), 6.87 (d, *J* = 8.0 Hz, 1H), 6.50 (d, *J* = 5.7 Hz, 1H), 4.93 (dd, *J* = 11.3, 4.6 Hz, 1H), 4.18 (dd, *J* = 7.9, 6.1 Hz, 1H), 3.90 (s, 3H), 2.48 (dd, *J* = 12.4, 4.7 Hz, 1H), 2.22 – 2.16 (m, 1H). ^{13}C NMR (126 MHz, $CDCl_3$) δ 166.84, 159.47, 144.97, 129.72, 129.57, 128.97, 127.41, 125.72, 124.71, 121.40, 120.52, 115.26, 110.51, 109.46, 79.56, 52.09, 47.38, 42.22. Enantiomeric excess:

81%, determined by HPLC (Chiralpak AD-H, hexane/*i*-PrOH = 95/5; flow rate 0.8 ml/min; 25 ℃; 220 nm), first peak: t_R = 21.6 min, second peak: t_R = 23.5 min; HRMS (ESI) m/z calcd. for $C_{18}H_{16}NaO_4$ $[M+Na]^+$ = 319.0941, found = 319.0940; IR spectrum (neat) (cm^{-1}) = 2974, 2884, 1381, 1275, 1198, 1086, 947, 880, 733, 623.

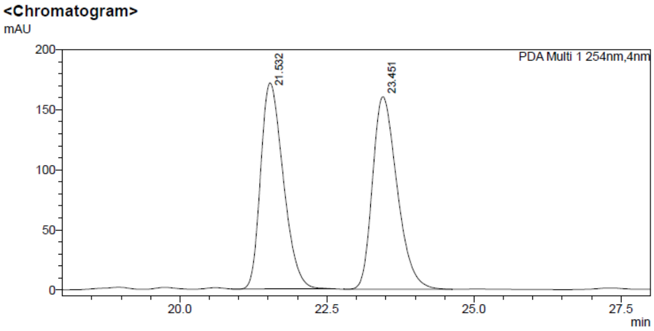

<Peak Table>
PDA Ch1 254nm

Peak#	Ret. Time	Height	Height%	Area	Area%
1	21.532	171555	51.691	4588711	49.514
2	23.451	160333	48.309	4678727	50.486
Total		331888	100.000	9267438	100.000

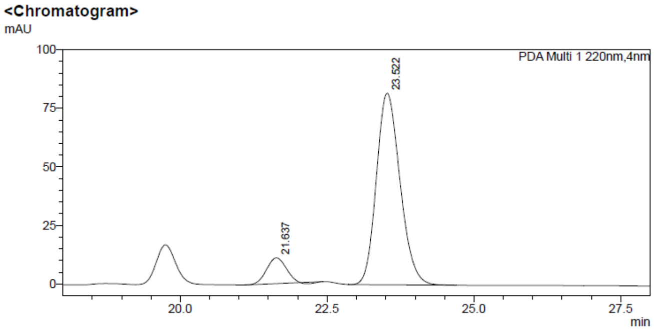

<Peak Table>
PDA Ch1 220nm

Peak#	Ret. Time	Height	Height%	Area	Area%
1	21.637	10994	11.852	247571	9.734
2	23.522	81764	88.148	2295797	90.266
Total		92757	100.000	2543368	100.000

(2*R*,3a*S*,8a*R*)-2-(benzofuran-5-yl)-2,3,3a,8a-tetrahydrofuro[2,3-*b*]benzofuran

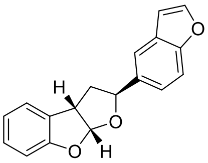

6ad; pale yellow oil (hexane/Et_2O = 20:1, 68% isolated yield); $[\alpha]_D^{20}$ = - 48.694 (*c* = 0.475, CH_2Cl_2); ^1H NMR (500 MHz, $CDCl_3$) δ 7.59 (d, *J* = 2.2 Hz, 1H), 7.56 (d, *J* = 1.4 Hz, 1H), 7.45 (d, *J* = 8.5 Hz, 1H), 7.26 – 7.22 (m, 2H), 7.21 – 7.18 (m, 1H), 6.95 (dd, *J* = 10.8, 4.0 Hz, 1H), 6.88 (d, *J* = 8.0 Hz, 1H), 6.72 – 6.71 (m, 1H), 6.50 (d, *J* = 5.8 Hz, 1H), 4.98 (dd, *J* = 11.3, 4.6 Hz, 1H), 4.16 (dd, *J* = 7.9, 6.2 Hz, 1H), 2.45 (dd, *J* = 12.4, 4.6 Hz, 1H), 2.30 – 2.24 (m, 1H). ^{13}C NMR (126 MHz, $CDCl_3$) δ 159.57, 154.59, 145.42, 134.09, 128.81, 127.80, 127.40, 124.71, 122.46, 121.22, 118.80, 111.23, 110.49, 109.36, 106.55, 80.35, 47.40, 42.53. Enantiomeric excess: 94%, determined by HPLC (Chiralpak AD-H, hexane/*i*-PrOH = 95/5; flow rate 0.8 ml/min; 25 ℃; 254 nm), first peak: t_R = 16.9 min, second peak: t_R = 18.9 min; HRMS (ESI) m/z calcd. for $C_{18}H_{14}NaO_3$ $[M+Na]^+$ = 301.0835, found = 301.0838; IR spectrum (neat) (cm^{-1}) = 2980, 2879, 1597, 1460, 1323, 1248, 1180, 1126, 1070, 993, 889, 814, 736.

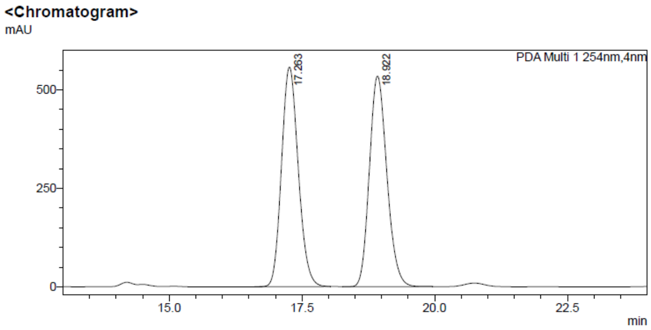

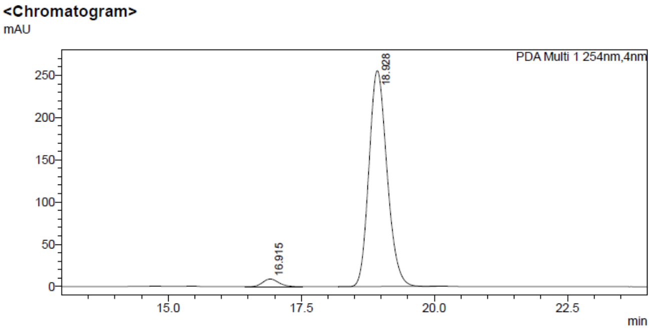

<Peak Table>
PDA Ch1 254nm

Peak#	Ret. Time	Height	Height%	Area	Area%
1	17.263	556615	51.030	12239371	49.976
2	18.922	534141	48.970	12251189	50.024
Total		1090756	100.000	24490560	100.000

<Peak Table>
PDA Ch1 254nm

Peak#	Ret. Time	Height	Height%	Area	Area%
1	16.915	9014	3.408	185627	2.976
2	18.928	255490	96.592	6051369	97.024
Total		264505	100.000	6236996	100.000

2-((2*R*,3a*S*,8aR)-2,3,3a,8a-tetrahydrofuro[2,3-*b*]benzofuran-2-yl)quinoline

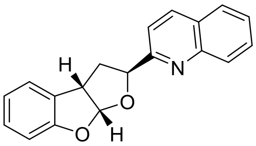

6ae; yellow solid (hexane/Et_2O = 8:1, 53% isolated yield); m.p. = 126-128 ^oC; $[\alpha]_D^{20}$ = - 9.2 (*c* = 0.4, CH_2Cl_2); ^1H NMR (500 MHz, $CDCl_3$) δ 8.18 (d, *J* = 8.5 Hz, 1H), 8.02 (d, *J* = 8.5 Hz, 1H), 7.81 (d, *J* = 8.1 Hz, 1H), 7.71 – 7.67 (m, 1H), 7.63 (d, *J* = 8.5 Hz, 1H), 7.52 (t, *J* = 7.5 Hz, 1H), 7.24 (d, *J* = 7.4 Hz, 1H), 7.19 (td, *J* = 7.4, 0.6 Hz, 1H), 6.95 (t, *J* = 7. 4 Hz, 1H), 6.88 (d, *J* = 8.1 Hz, 1H), 6.56 (d, *J* = 5.6 Hz, 1H), 5.16 (dd, *J* = 11.3, 4.8 Hz, 1H), 4.20 (dd, *J* = 7.7, 6.2 Hz, 1H), 2.69 (dd, *J* = 12.3, 4.7 Hz, 1H), 2.44 – 2.37 (m, 1H). ^{13}C NMR (126 MHz, $CDCl_3$) δ 159.82, 159.38, 147.33, 137.07, 129.71, 129.54, 128.89, 127.62, 127.49 (d, J = 41.1 Hz), 126.44, 124.92, 121.42, 118.20, 115.43, 110.80, 109.41, 81.43, 47.32, 40.84. Enantiomeric excess: 89%, determined by HPLC (Chiralpak IF, hexane/*i*-PrOH = 95/5; flow rate 0.8 ml/min; 25 ℃; 254 nm), first peak: t_R = 19.4 min, second peak: t_R = 25.2 min; HRMS (ESI) m/z calcd. for $C_{19}H_{16}NO_2$ $[M+H]^+$ = 290.1176, found = 290.1185; IR spectrum (neat) (cm^{-1}) = 2976, 2878, 1381, 1321, 1198, 1086, 947, 880, 752, 631.

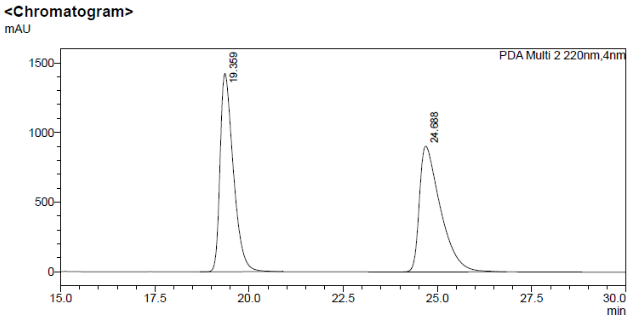

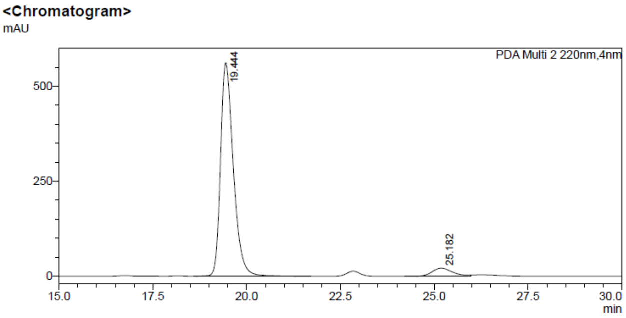

<Peak Table>
PDA Ch2 220nm

Peak#	Ret. Time	Height	Height%	Area	Area%
1	19.359	1423601	61.218	34747305	49.809
2	24.688	901855	38.782	35013559	50.191
Total		2325456	100.000	69760864	100.000

<Peak Table>
PDA Ch2 220nm

Peak#	Ret. Time	Height	Height%	Area	Area%
1	19.444	561822	96.361	13316060	94.259
2	25.182	21215	3.639	811086	5.741
Total		583038	100.000	14127145	100.000

(3aS,8aR)-8a-methyl-2,3,3a,8a-tetrahydrofuro[2,3-b]benzofuran

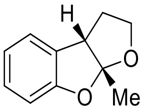

6af; pale yellow oil (hexane/Et_2O = 20:1, 47% isolated yield); $[\alpha]_D^{20}$ = -70.12 (*c* = 0.33, CH_2Cl_2); Enantiomeric excess: 83%, determined by HPLC (Chiralpak OJ-H, hexane/*i*-PrOH = 98/2; flow rate 0.5 ml/min; 25 ℃; 205 nm), first peak: t_R = 14.4 min, second peak: t_R = 17.3 min. (Please refer to Mazet's work for $^1H/^{13}C$ NMR and IR)

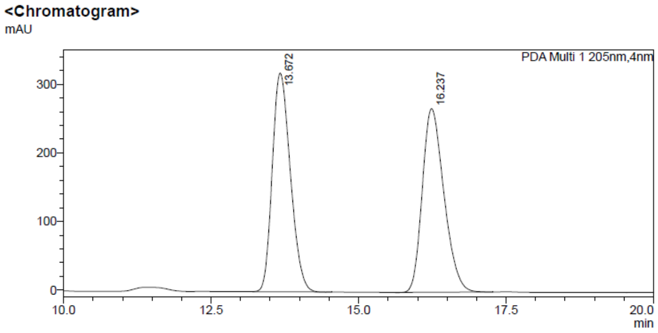

<Peak Table>
PDA Ch1 205nm

Peak#	Ret. Time	Height	Height%	Area	Area%
1	13.672	318971	54.366	6804339	49.869
2	16.237	267739	45.634	6840208	50.131
Total		586710	100.000	13644547	100.000

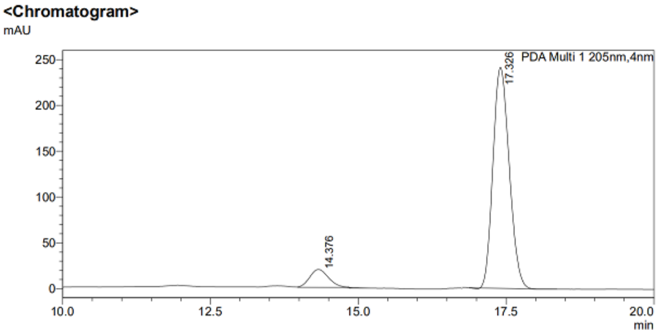

<Peak Table>
PDA Ch1 205nm

Peak#	Ret. Time	Height	Height%	Area	Area%
1	14.376	19538	7.490	432982	8.560
2	17.326	241312	92.510	4625210	91.440
Total		260849	100.000	5058192	100.000

5. Absolute Configuration of 3 and 6

X-ray structure of **3aa** and **3ac**:

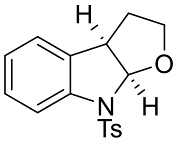

3aa

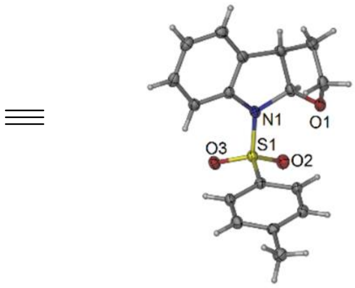

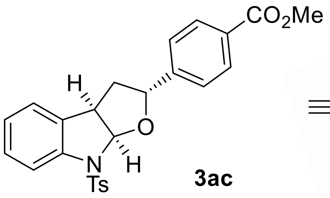

3ac

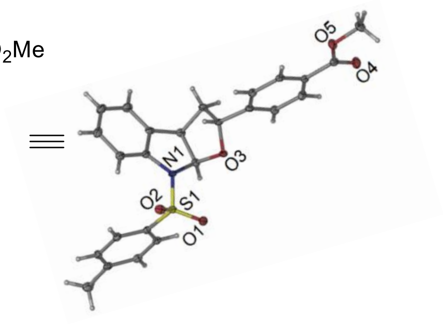

The configuration of **6aa-6ha** was determined by comparing the optical rotation with the reported ones in Mazet's work (see ref. 1).

For instance:

	Our work	Mazet's work
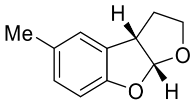 **6ca**	$[\alpha]_D^{20}$ = -168.114 (c = 0.5, CH_2Cl_2)	$[\alpha]_D^{23}$ = -172.0 (c = 0.85, CH_2Cl_2)
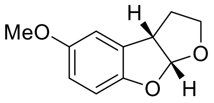 **6da**	$[\alpha]_D^{20}$ = -182.367 (c = 0.54, CH_2Cl_2)	$[\alpha]_D^{23}$ = -166.8 (c = 0.54, CH_2Cl_2)
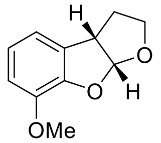 **6ha**	$[\alpha]_D^{20}$ = -113.542 (c = 0.625, CH_2Cl_2)	$[\alpha]_D^{23}$ = -108 (c = 0.81, CH_2Cl_2)

The configuration of **6ab-6ae** was determined by comparing the optical rotation and ^{1}H-^{1}H-NOSEY-NMR spectrum with the reported one in Mazet's work (see ref. 1).

For instance:

Our work		Mazet's work	
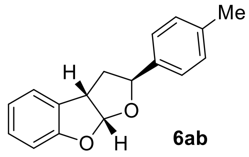 **6ab**	$[\alpha]_D^{20}$ = -54.179 (c = 0.5, CH_2Cl_2)	$[\alpha]_D^{23}$ = -136.7 (c = 0.49, CH_2Cl_2)	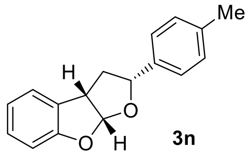 **3n**

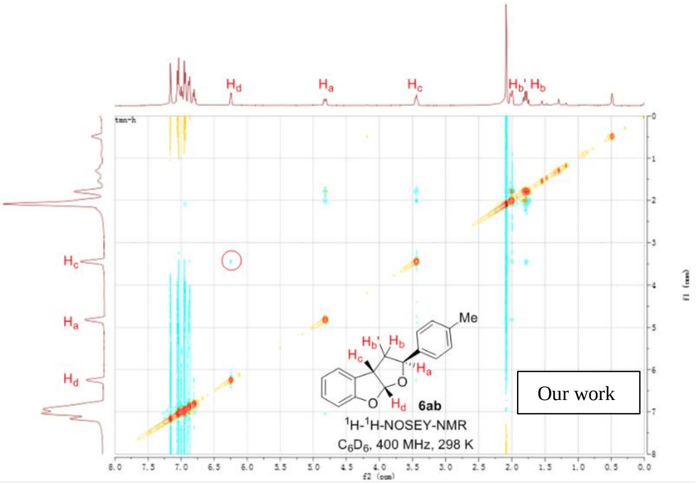

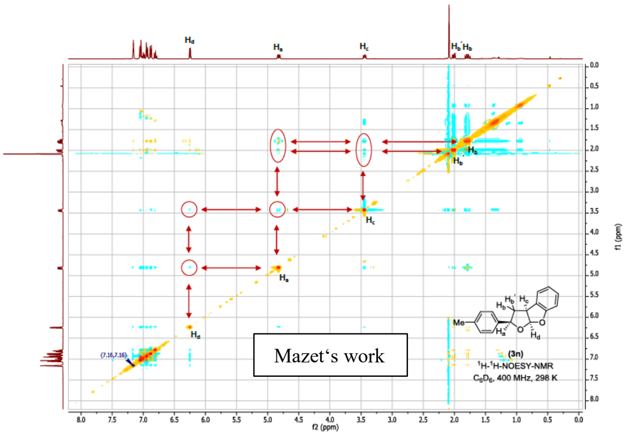

The configuration of new modified ***N*-Me-Xiang-Phos** was determined according to the reported ***N*-Me-Xu-Phos** in our previous work, due to the same one-pot synthesis approach (see ref. 3).

6. References

1 G. M. Borrajo-Calleja, V. Bizet, C. Mazet, J. *Am. Chem. Soc.* **2016**, *138*, 4014–4017.

2 Y.-Z. Chen, M.-L. Peng, D. Zhang, L.-P. Zhang, L.-Z. Wu, C.-H. Tung, *Tetrahedron*, **2006**, *62*, 10688-10693.

3 Z.-M. Zhang, B. Xu, Y. Qian, L. Wu, Y. Wu, L. Zhou, Y. Liu, J. Zhang, *Angew. Chem.* **2018**, *130*, 10530-10534; *Angew. Chem. Int. Ed.* **2018**, *57*, 10373-10377.

7. ^{1}H, ^{13}C, ^{19}F, ^{31}P Spectra for (*S*,*R*$_S$)-*N*-Me-X4/X5, 3 and 6

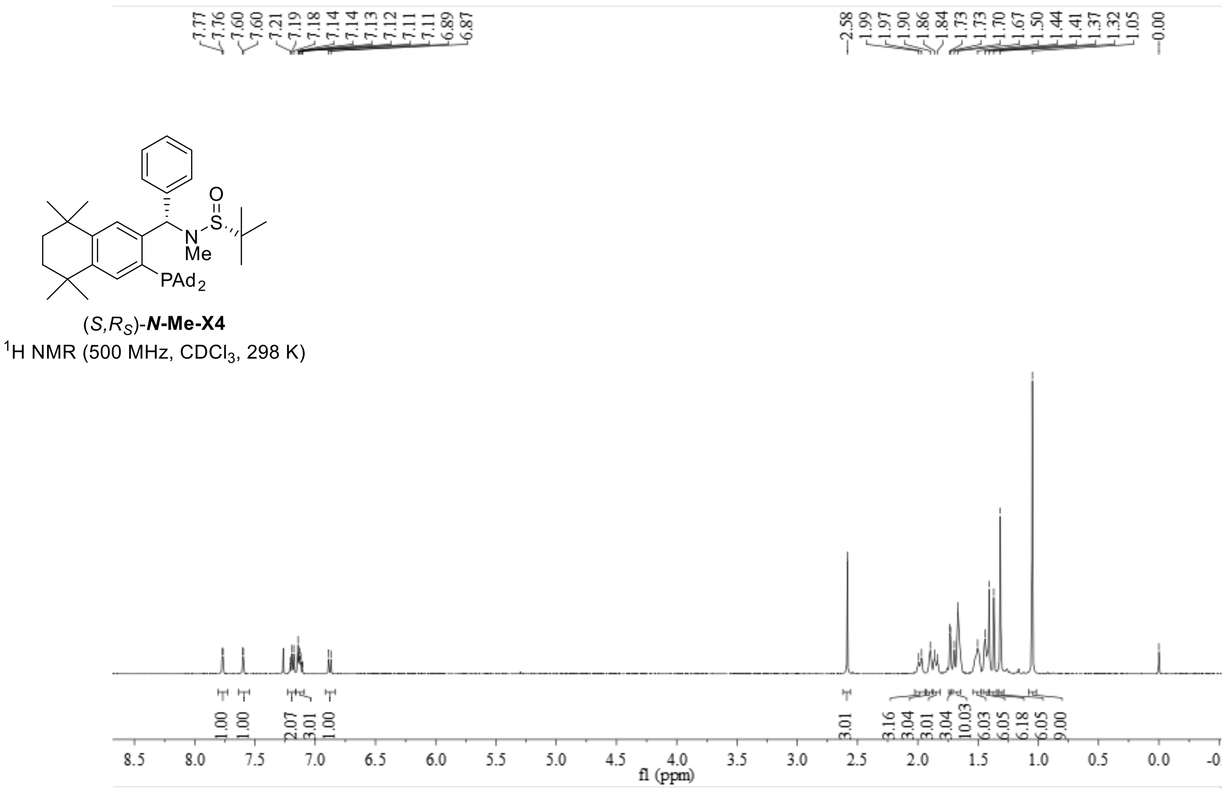

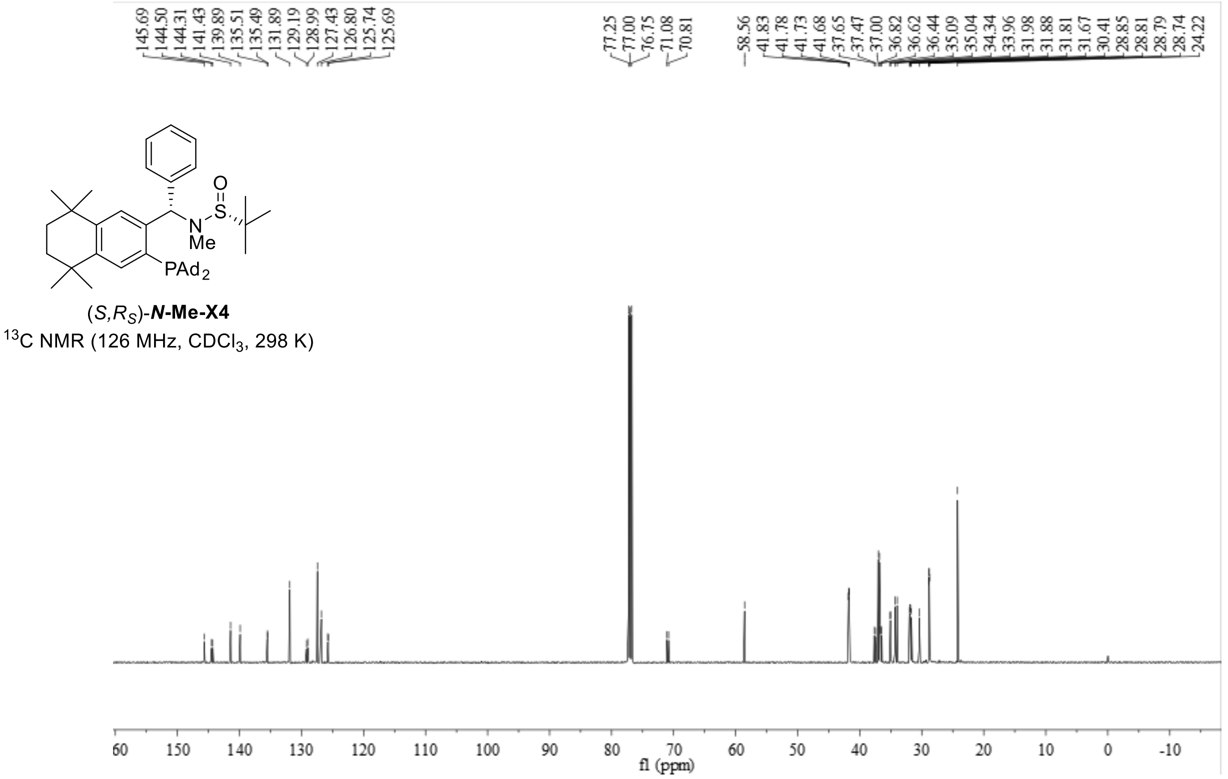

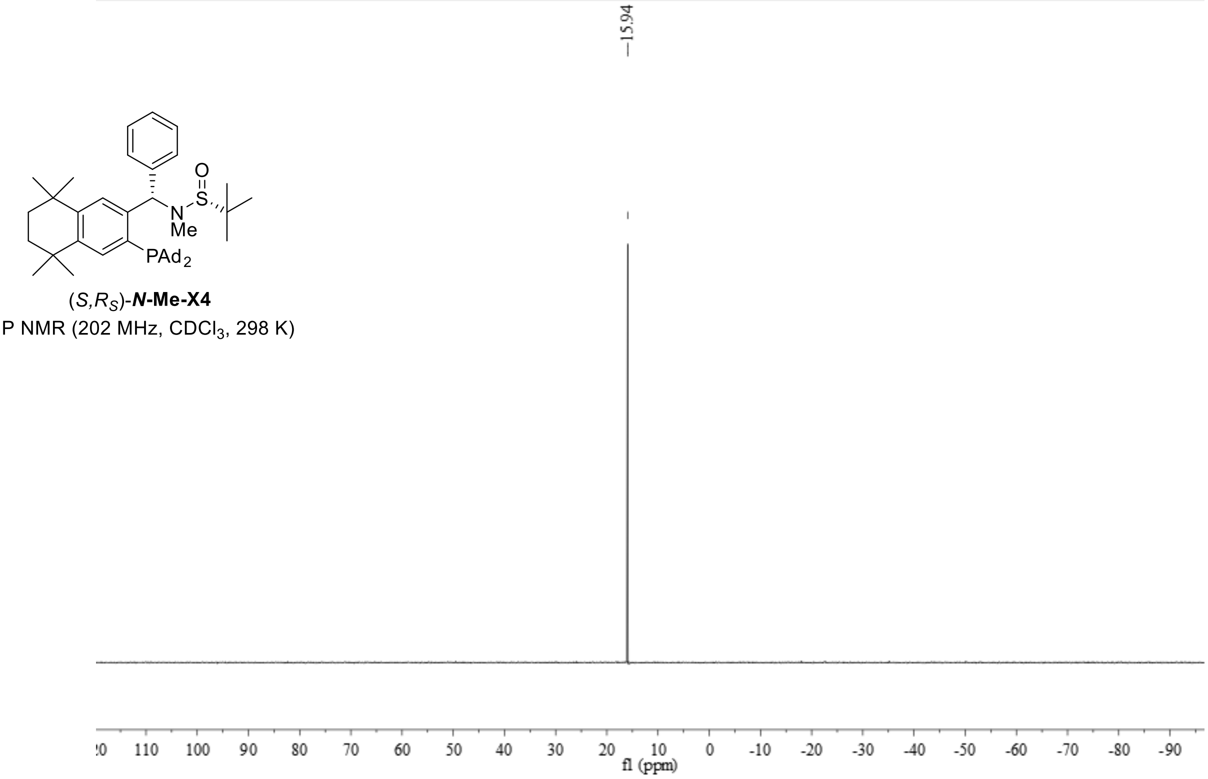

(S,R_S)-***N*-Me-X4**

^{31}P NMR (202 MHz, $CDCl_3$, 298 K)

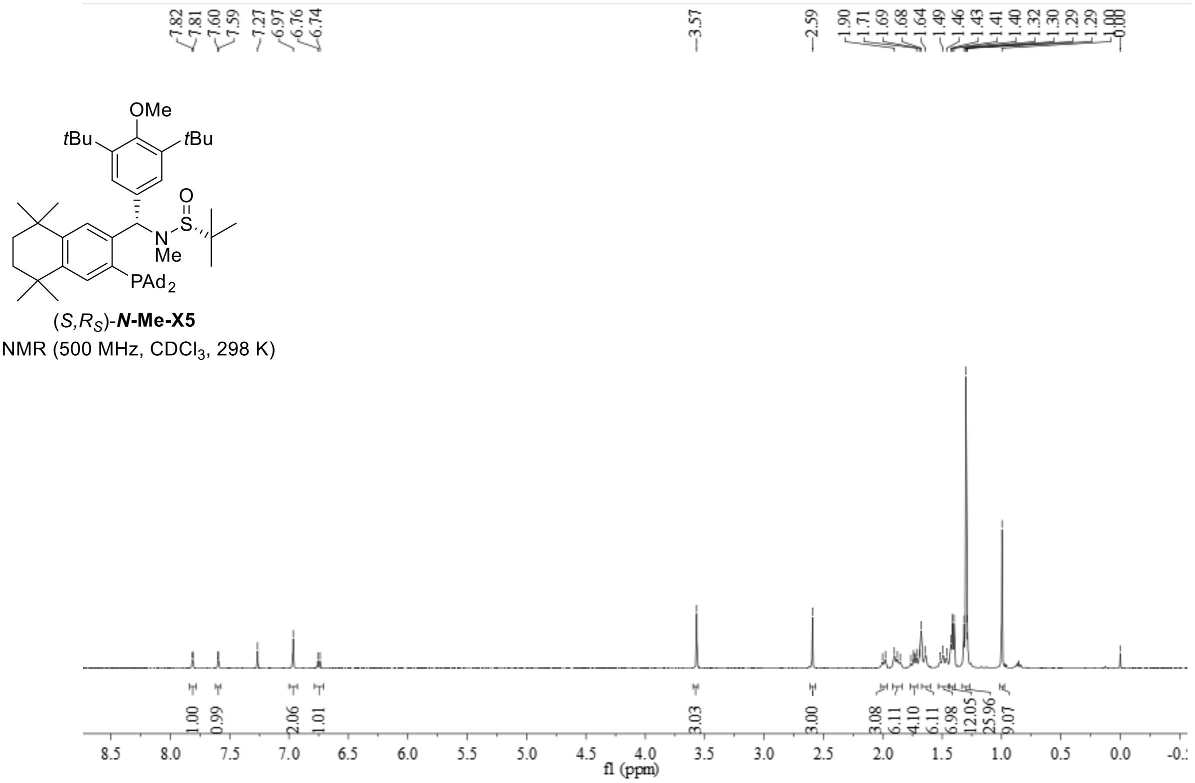

(S,R_S)-***N*-Me-X5**

^{1}H NMR (500 MHz, $CDCl_3$, 298 K)

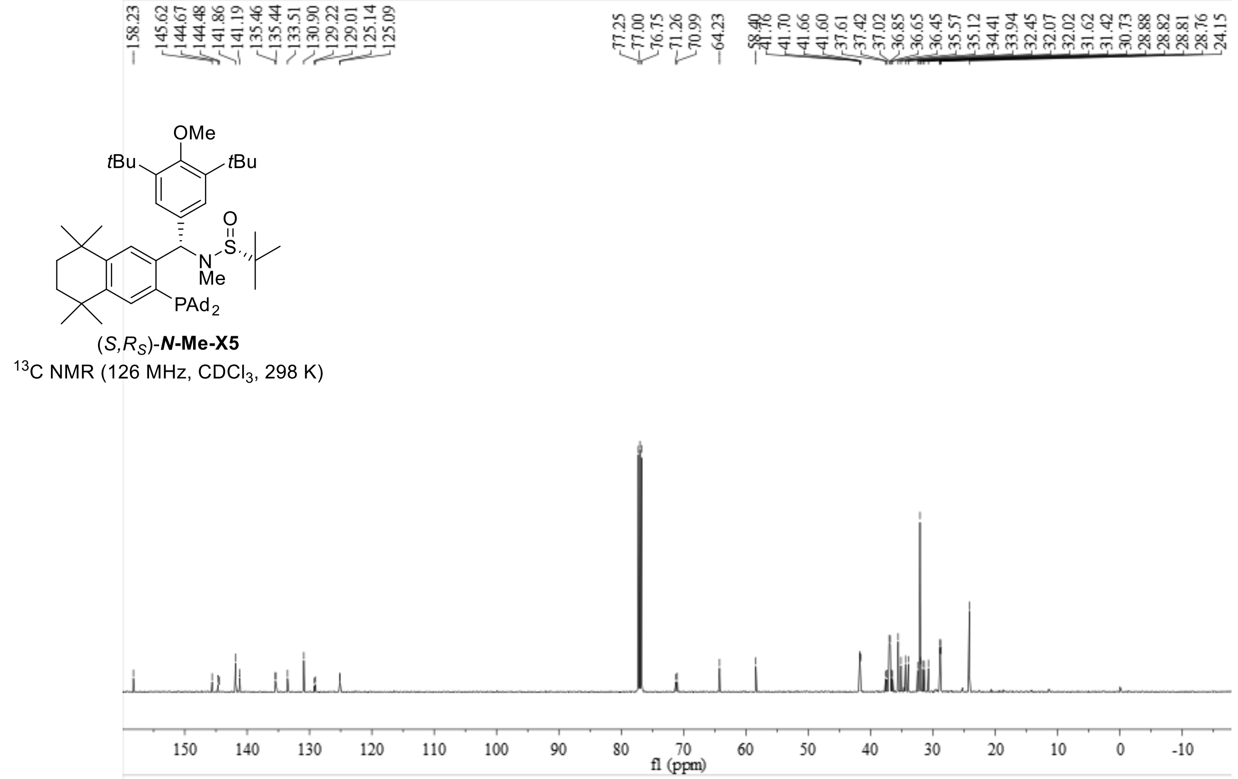

(S,R_S)-***N*-Me-X5**

^{13}C NMR (126 MHz, $CDCl_3$, 298 K)

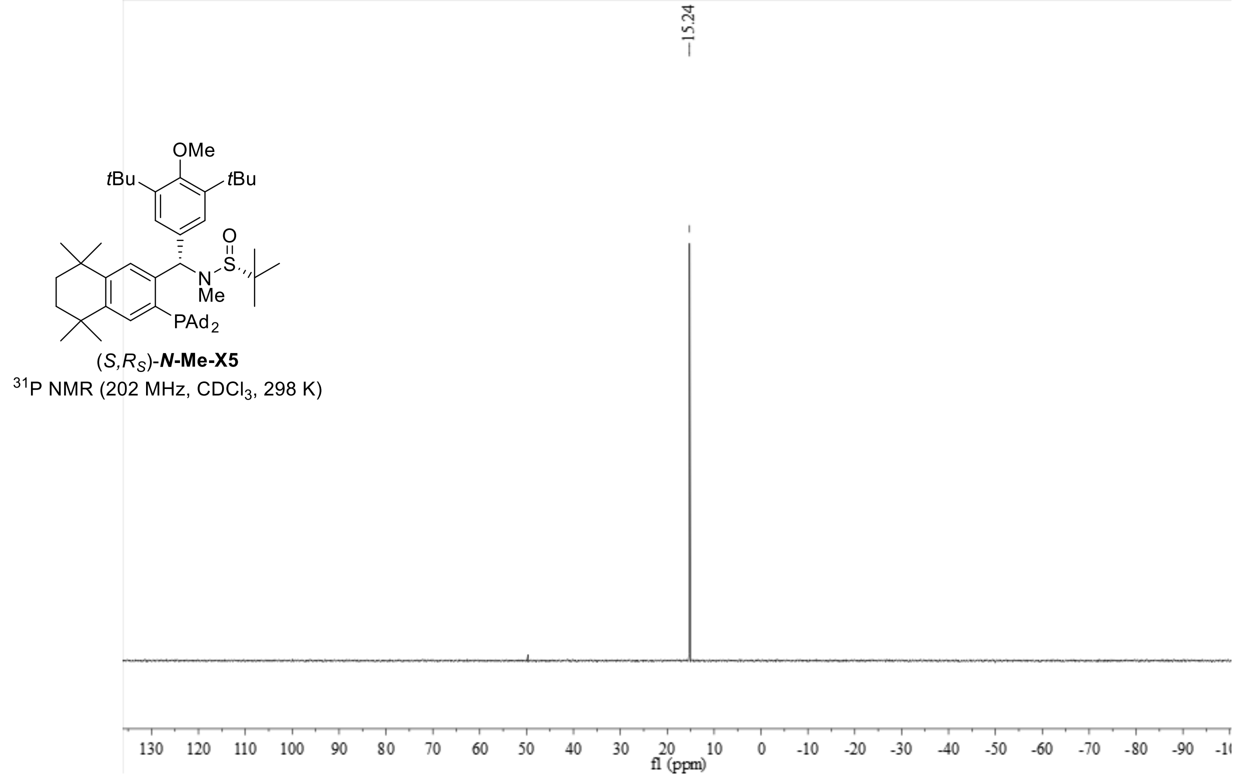

(S,R_S)-***N*-Me-X5**

^{31}P NMR (202 MHz, $CDCl_3$, 298 K)

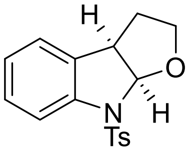

3aa

^{1}H NMR (500 MHz, $CDCl_3$, 298 K)

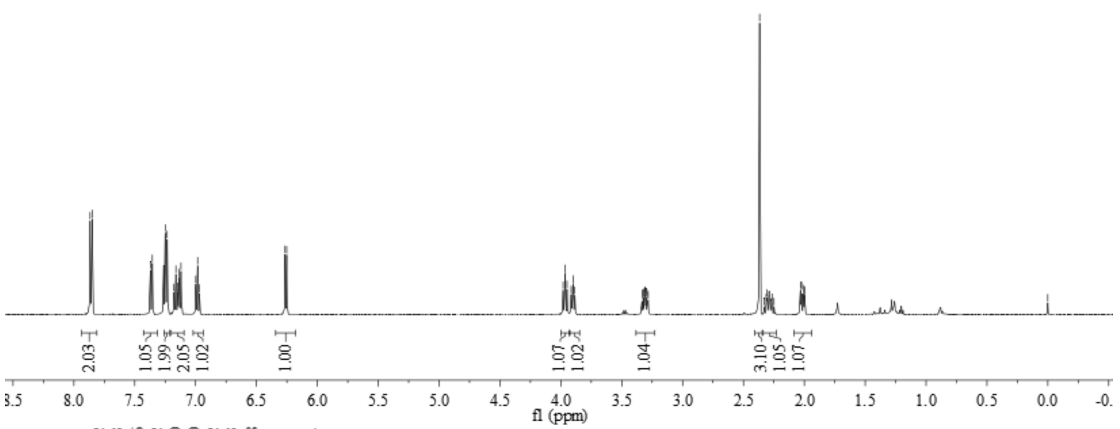

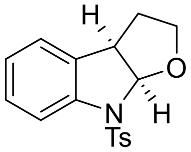

3aa

^{13}C NMR (126 MHz, $CDCl_3$, 298 K)

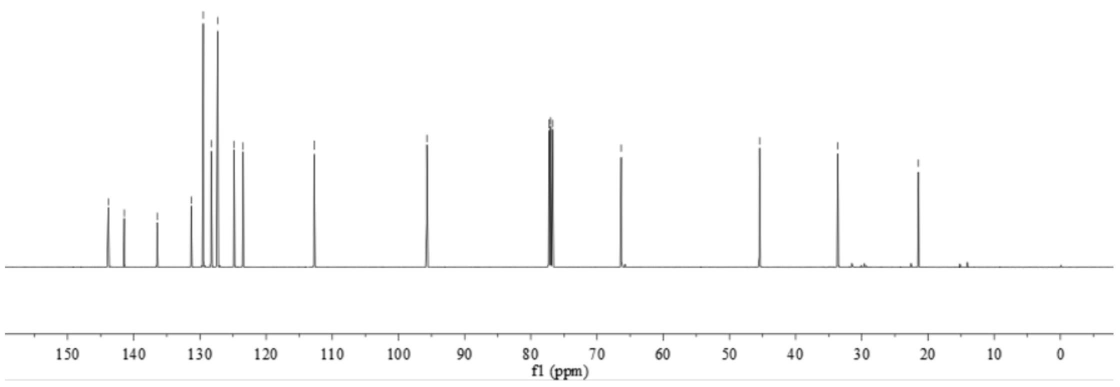

3ba

^{1}H NMR (500 MHz, $CDCl_3$, 298 K)

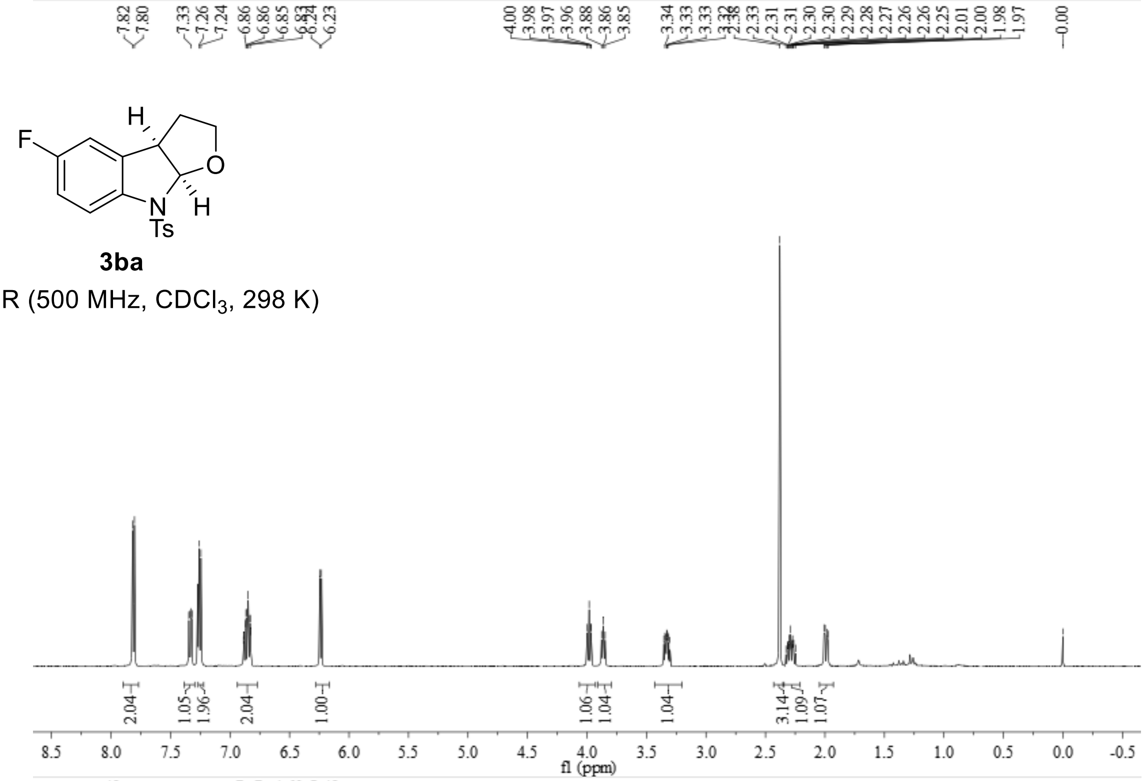

3ba

^{13}C NMR (126 MHz, $CDCl_3$, 298 K)

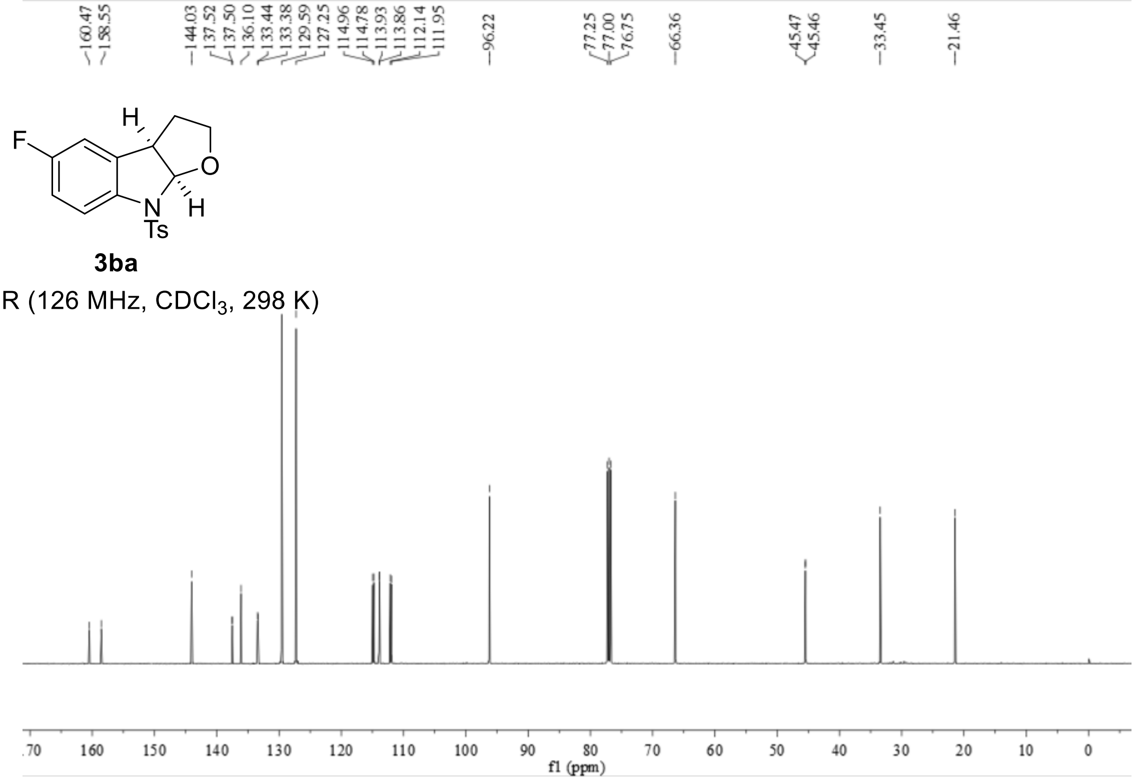

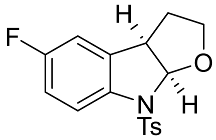

3ba

^{19}F NMR (376 MHz, $CDCl_3$, 298 K)

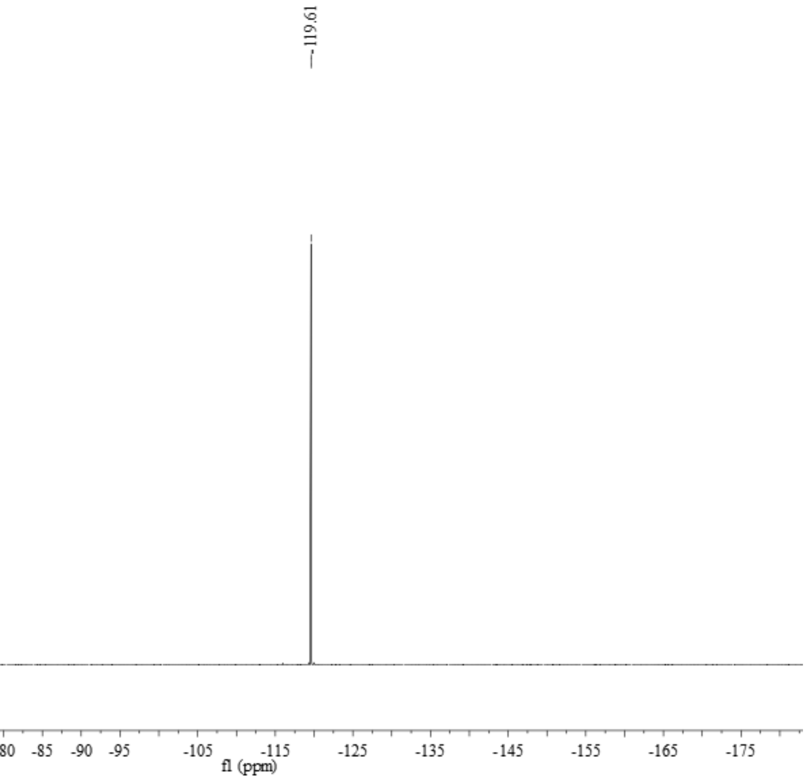

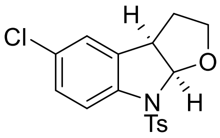

3ca

^{1}H NMR (500 MHz, $CDCl_3$, 298 K)

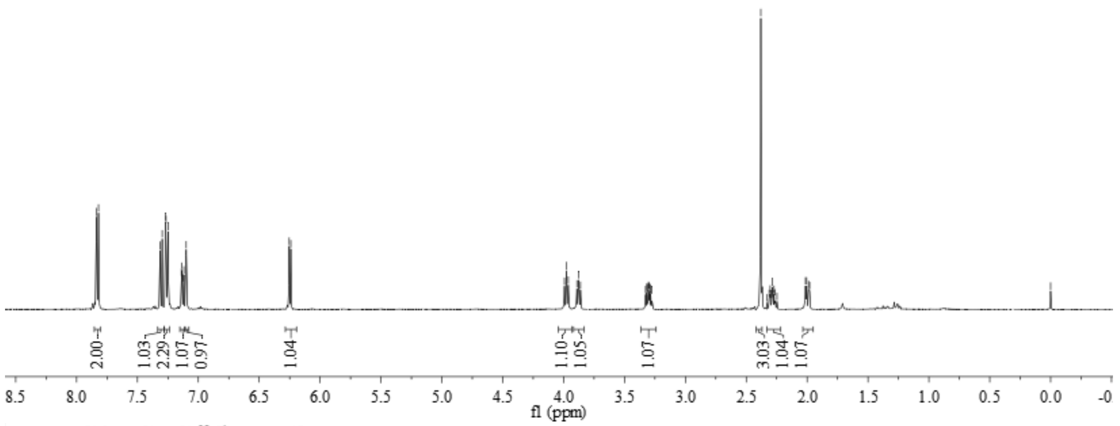

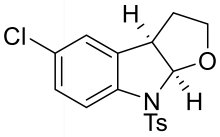

3ca

^{13}C NMR (126 MHz, $CDCl_3$, 298 K)

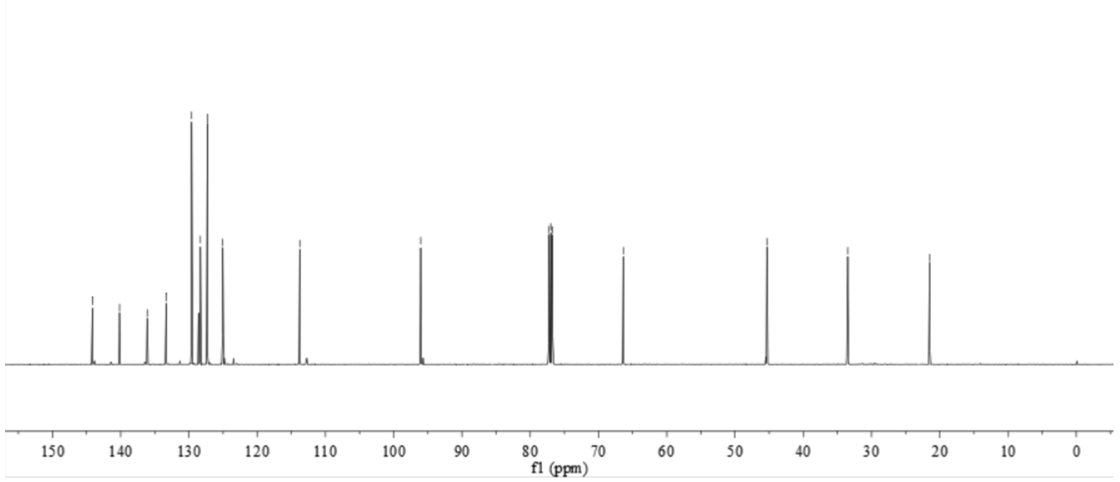

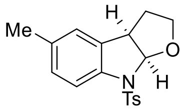

3da

^{1}H NMR (500 MHz, $CDCl_3$, 298 K)

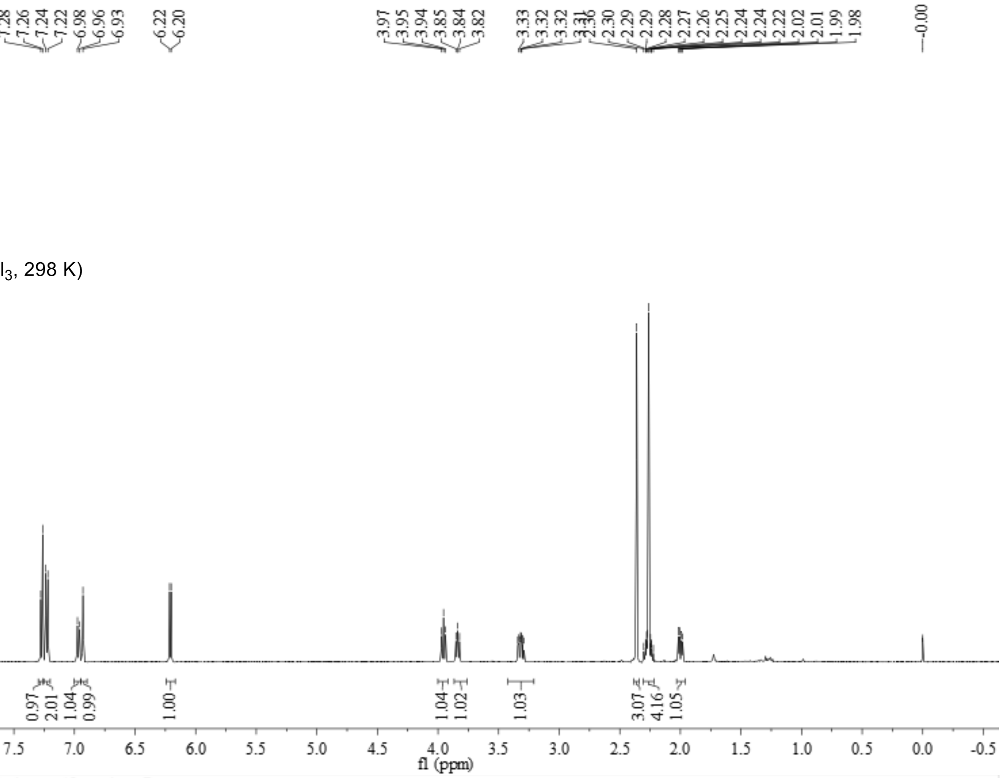

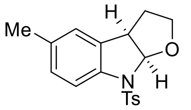

3da

^{13}C NMR (126 MHz, $CDCl_3$, 298 K)

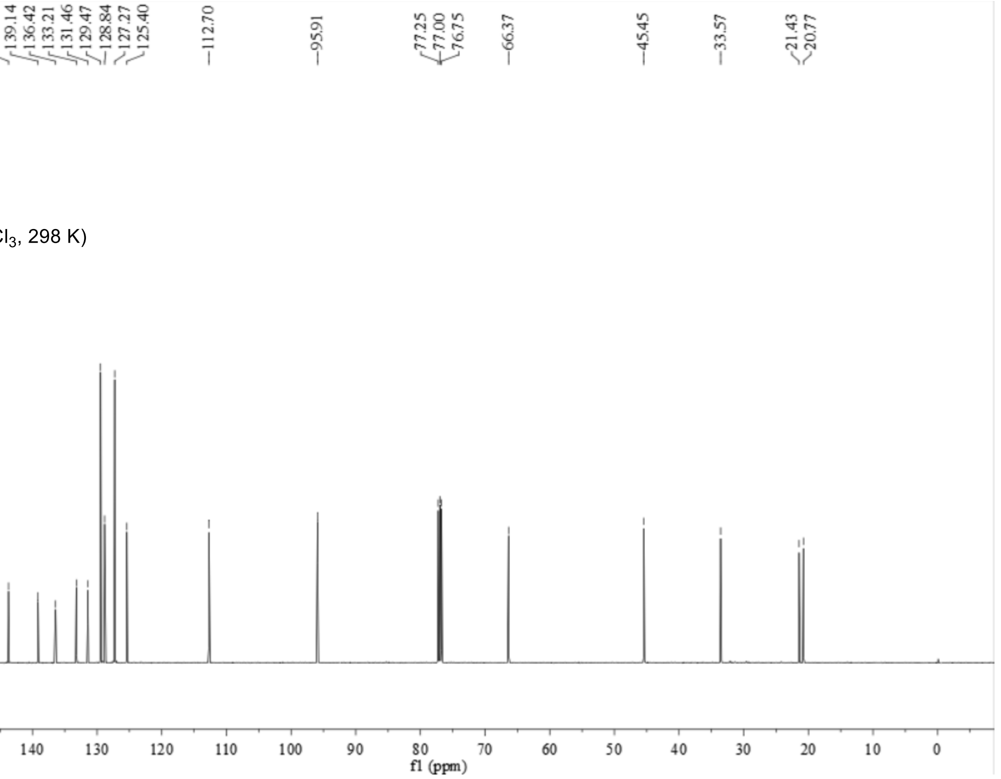

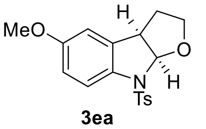

3ea

^{1}H NMR (500 MHz, $CDCl_3$, 298 K)

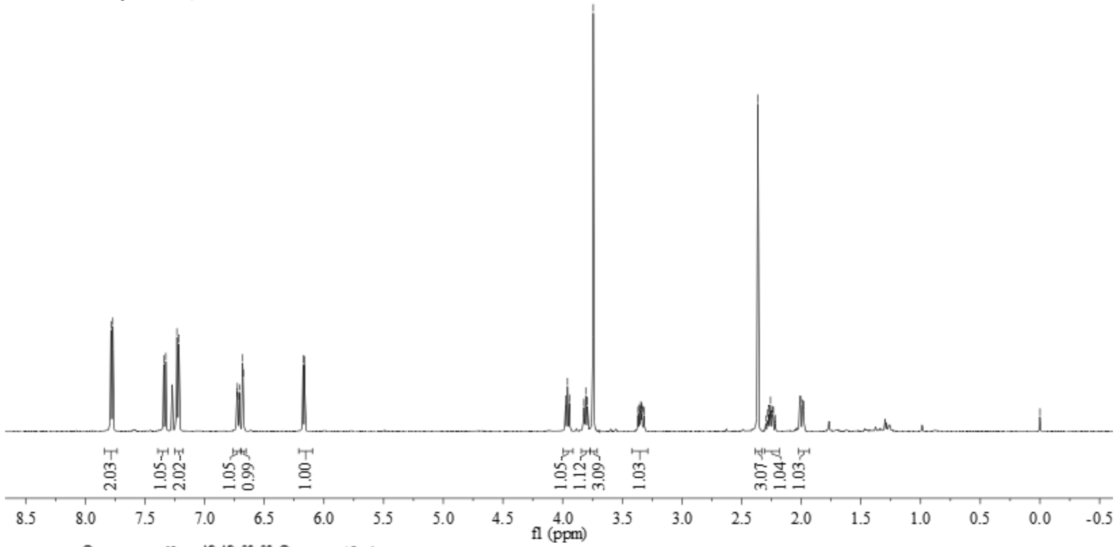

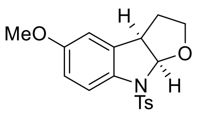

3ea

^{13}C NMR (126 MHz, $CDCl_3$, 298 K)

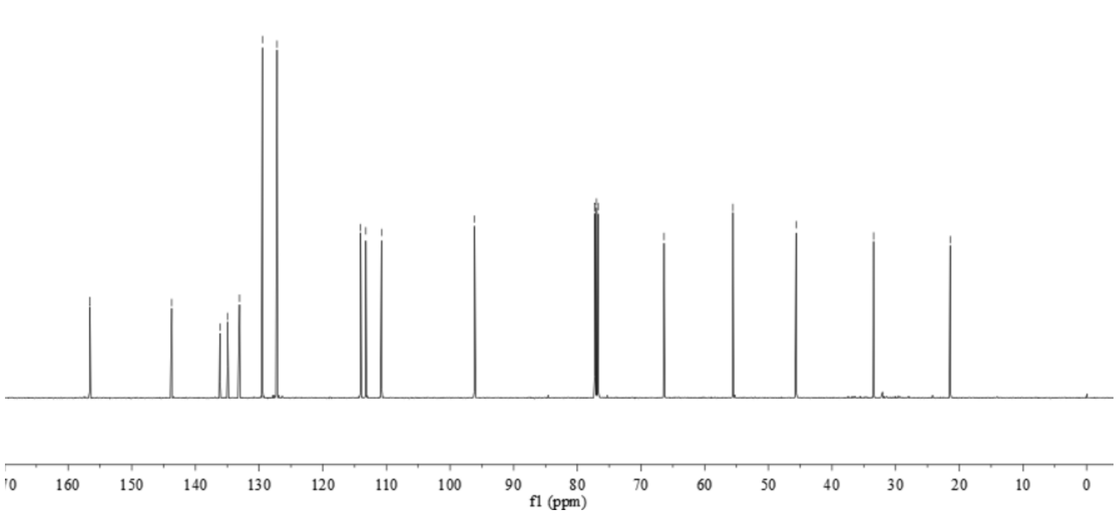

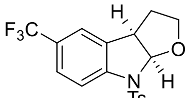

3fa

^{1}H NMR (500 MHz, $CDCl_3$, 298 K)

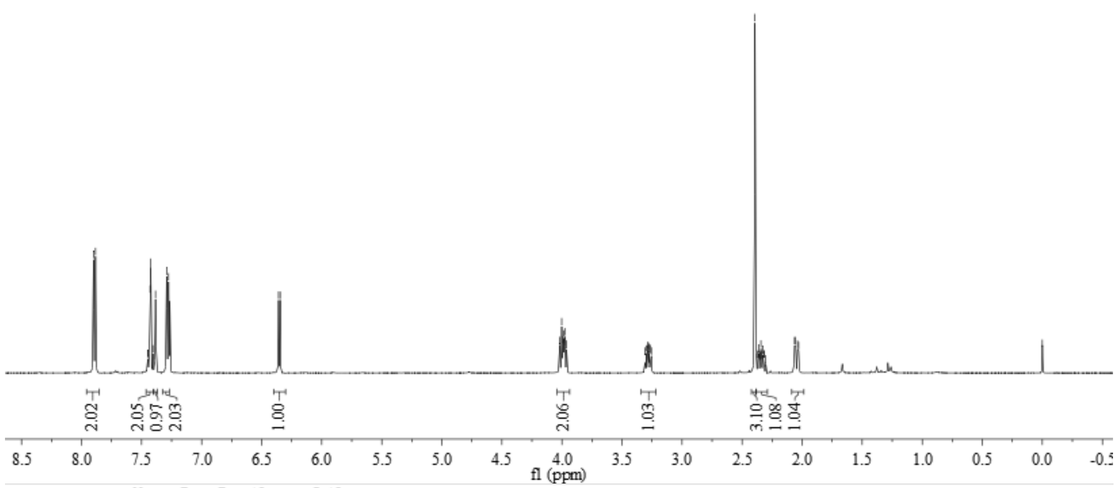

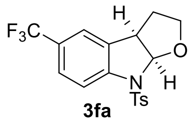

3fa

^{13}C NMR (126 MHz, $CDCl_3$, 298 K)

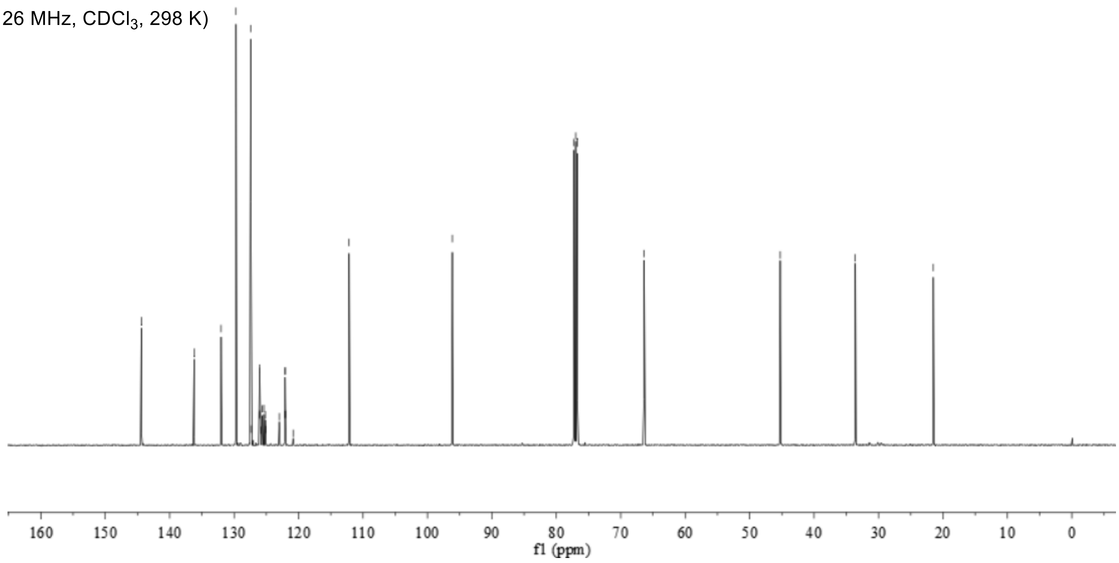

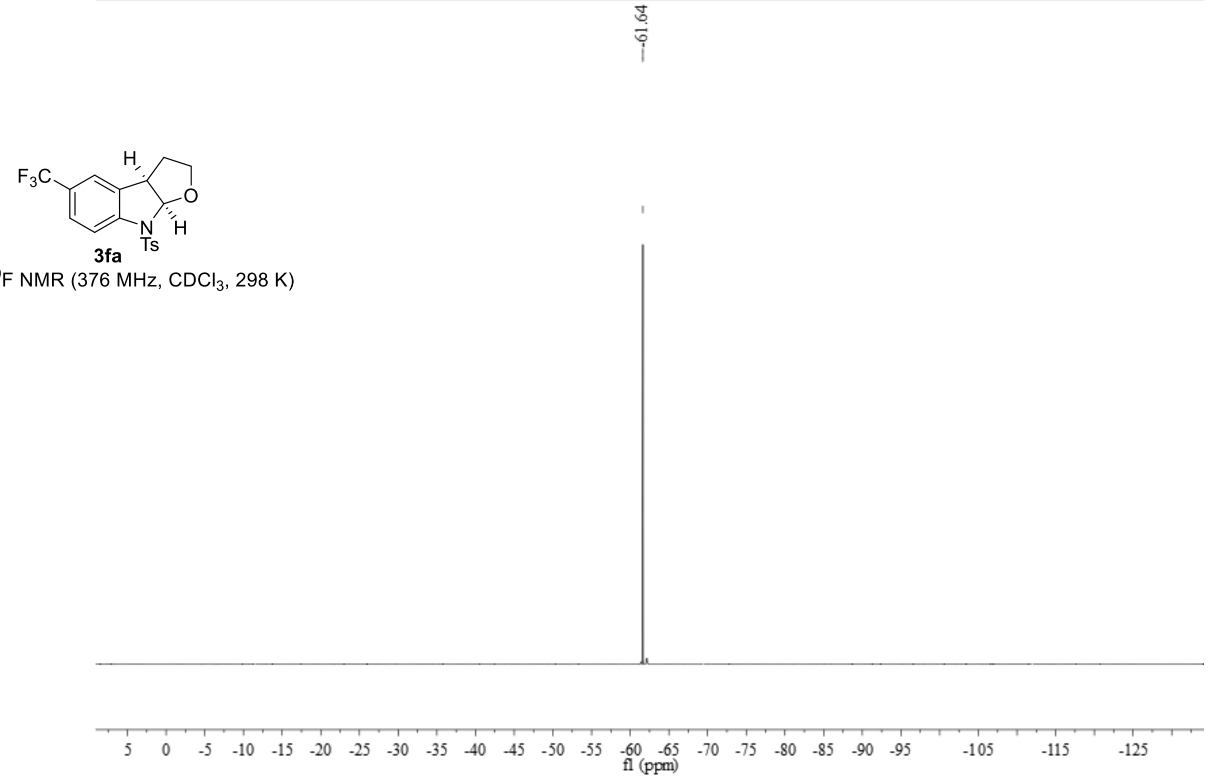

3fa

^{19}F NMR (376 MHz, $CDCl_3$, 298 K)

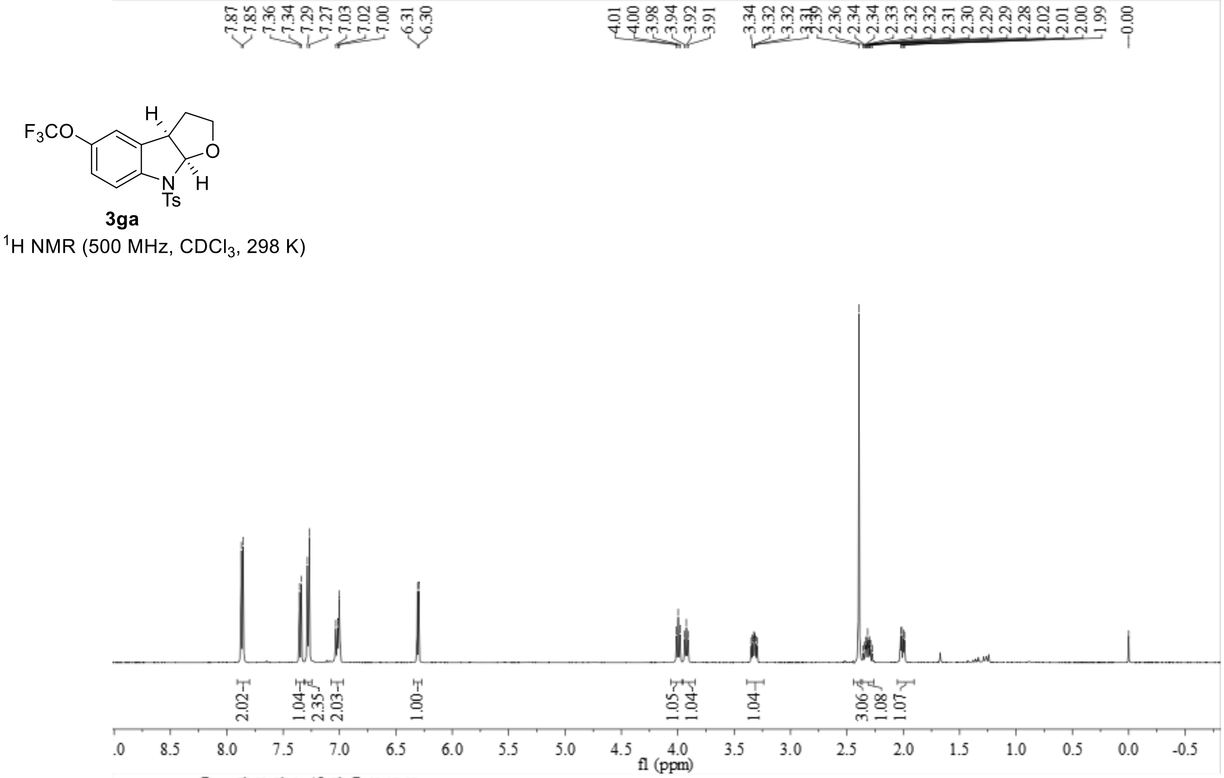
7.87
7.85
7.36
7.34
7.29
7.27
7.03
7.02
7.00
6.31
6.30
4.01
4.00
3.98
3.94
3.92
3.91
3.34
3.32
3.32
3.31
3.30
2.36
2.34
2.34
2.33
2.32
2.32
2.31
2.30
2.29
2.29
2.28
2.02
2.01
2.00
1.99
0.00
F3CO
H
O
N
H
Ts
3ga
1H NMR (500 MHz, CDCl3, 298 K)
2.02
1.04
2.35
2.03
1.00
1.05
1.04
1.04
3.06
1.08
1.07
f1 (ppm)

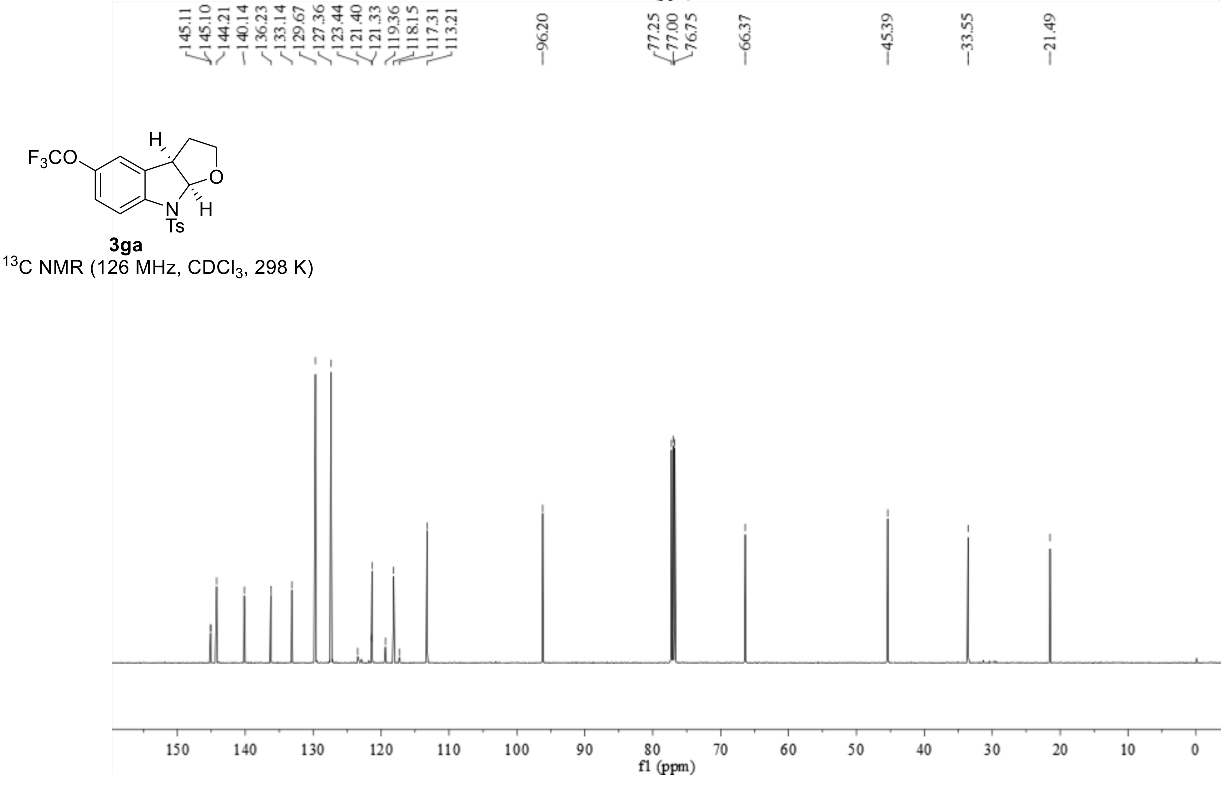
145.11
145.10
144.21
140.14
136.23
133.14
129.67
127.36
123.44
121.40
121.33
119.36
118.15
117.31
113.21
96.20
77.25
77.00
76.75
66.37
45.39
33.55
21.49
F3CO
H
O
N
H
Ts
3ga
13C NMR (126 MHz, CDCl3, 298 K)
f1 (ppm)

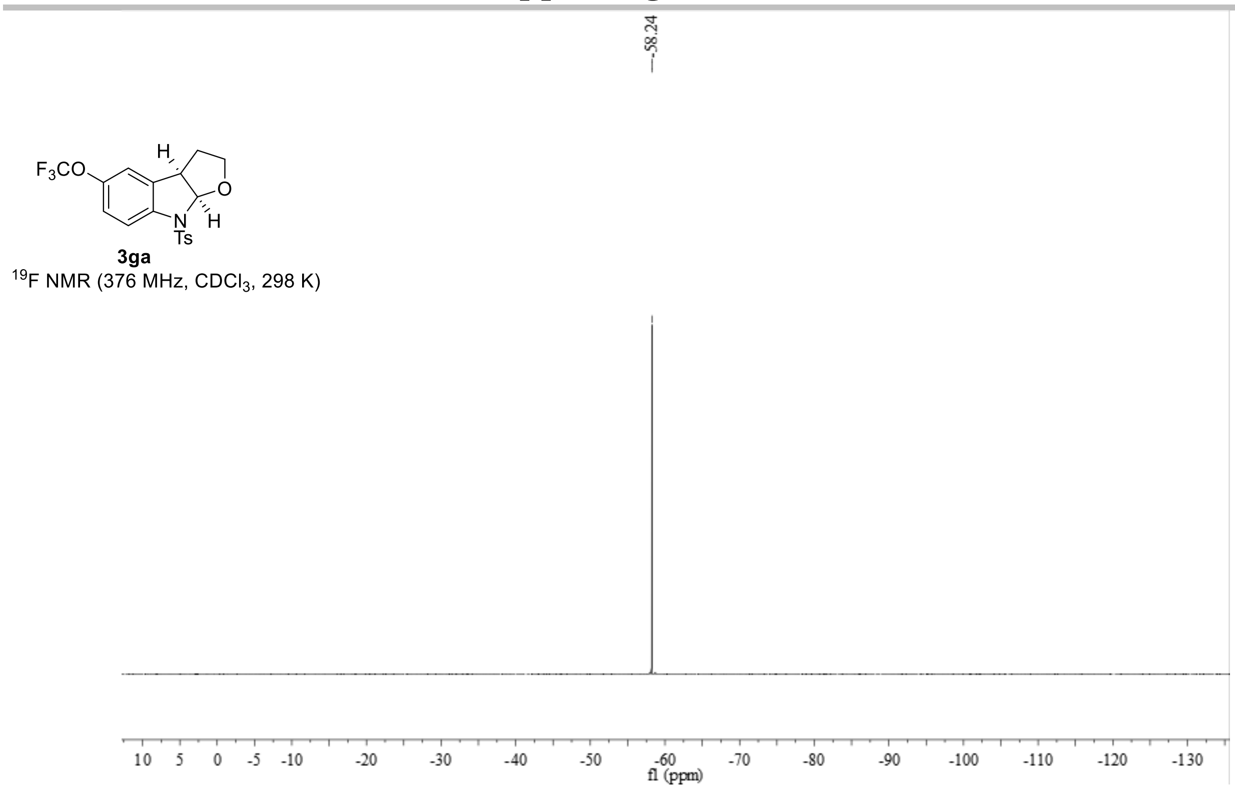
F3CO
H
O
N
H
Ts
3ga
^{19}F NMR (376 MHz, $CDCl_3$, 298 K)
-58.24
10 5 0 -5 -10 -20 -30 -40 -50 -60 -70 -80 -90 -100 -110 -120 -130
f1 (ppm)

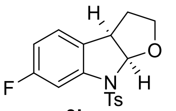

3ha

^{1}H NMR (500 MHz, $CDCl_3$, 298 K)

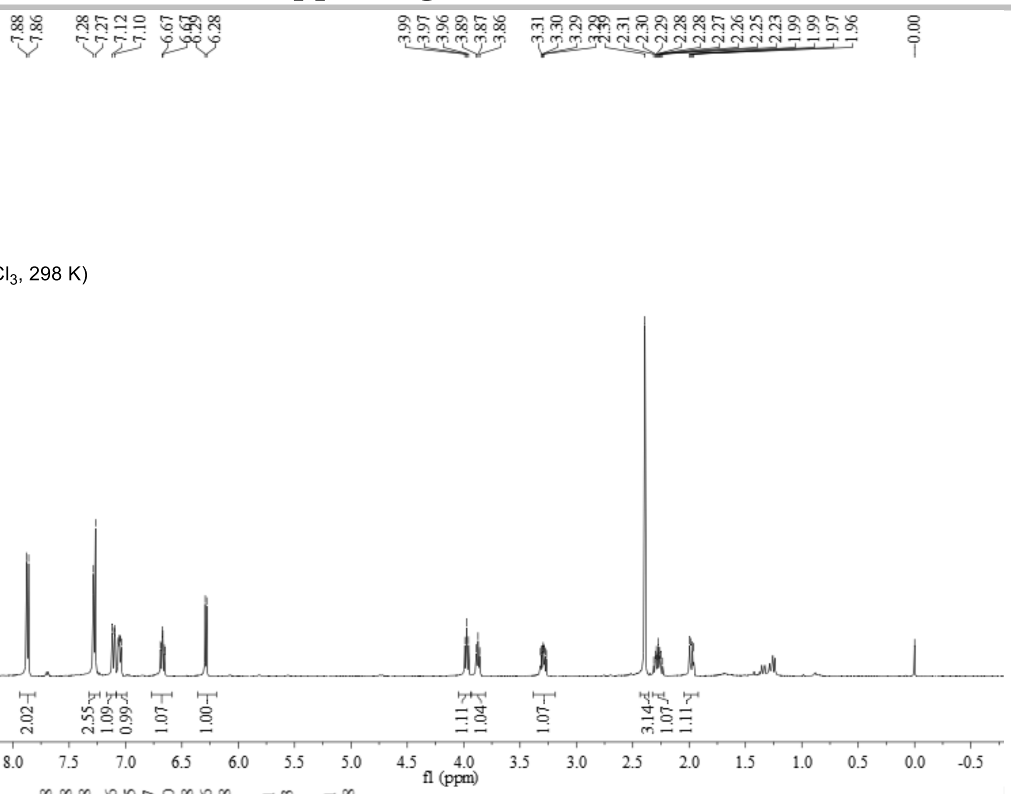

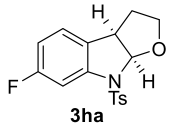

3ha

^{13}C NMR (126 MHz, $CDCl_3$, 298 K)

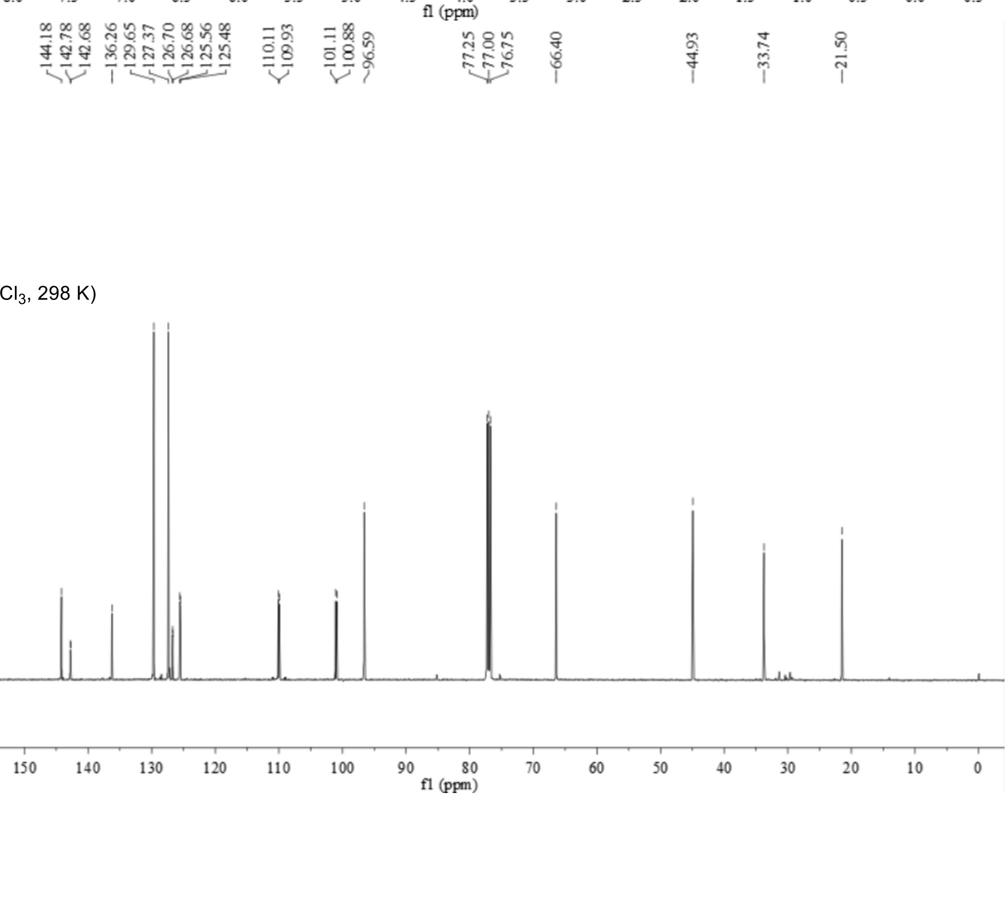

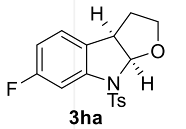

3ha

^{19}F NMR (282 MHz, $CDCl_3$, 298 K)

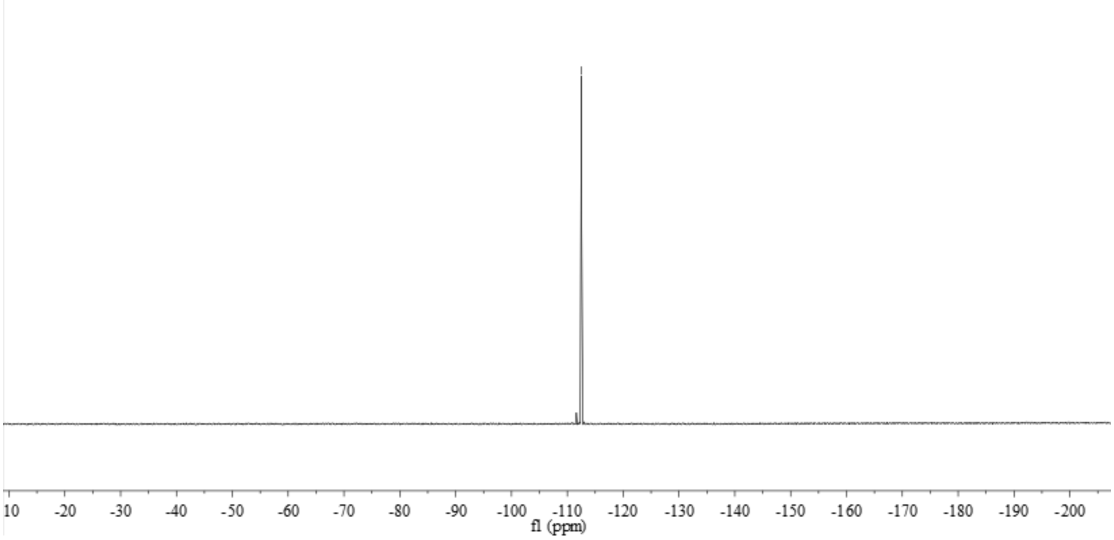

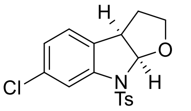

3ia

^{1}H NMR (500 MHz, $CDCl_3$, 298 K)

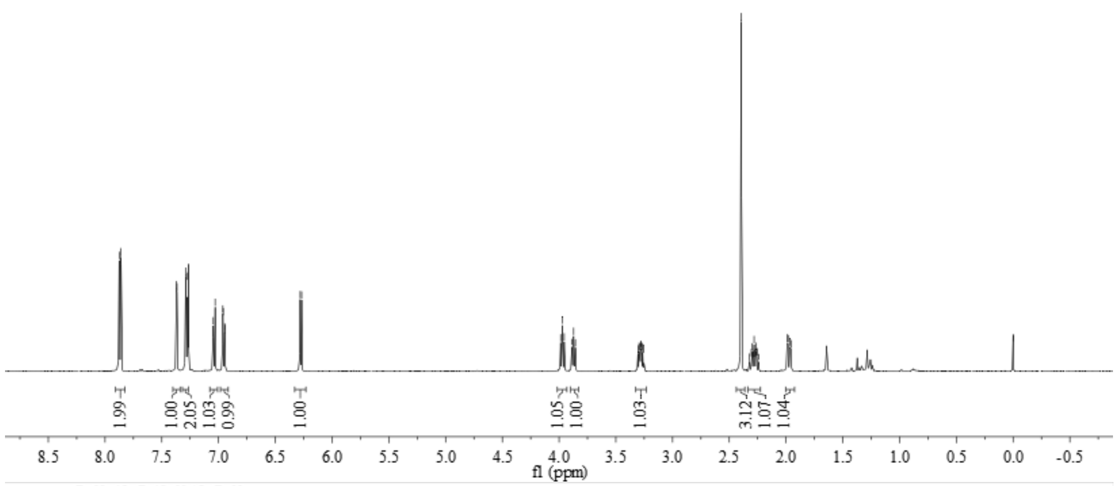

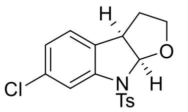

3ia

^{13}C NMR (126 MHz, $CDCl_3$, 298 K)

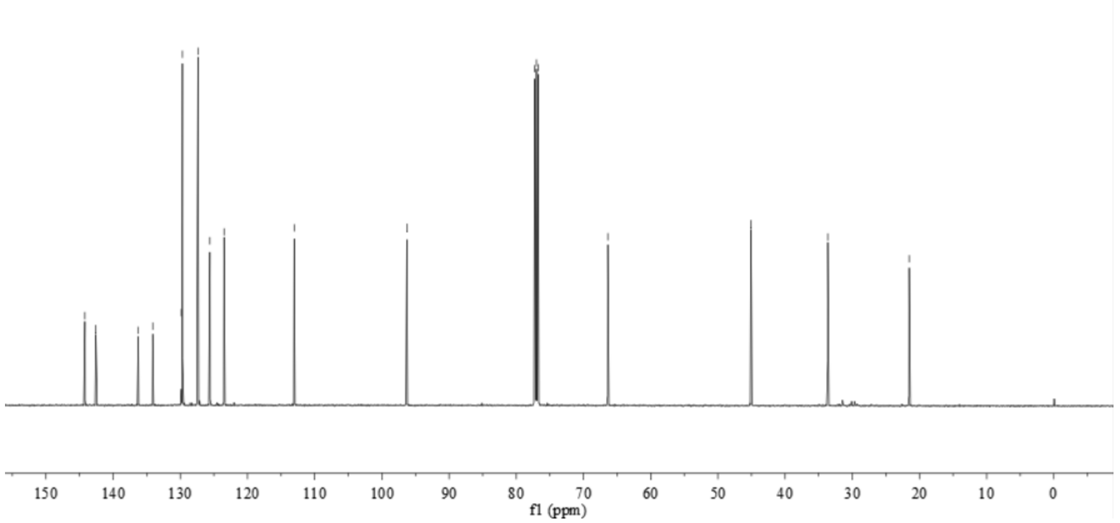

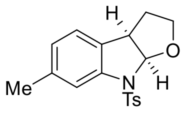

3ja
^{1}H NMR (500 MHz, $CDCl_3$, 298 K)

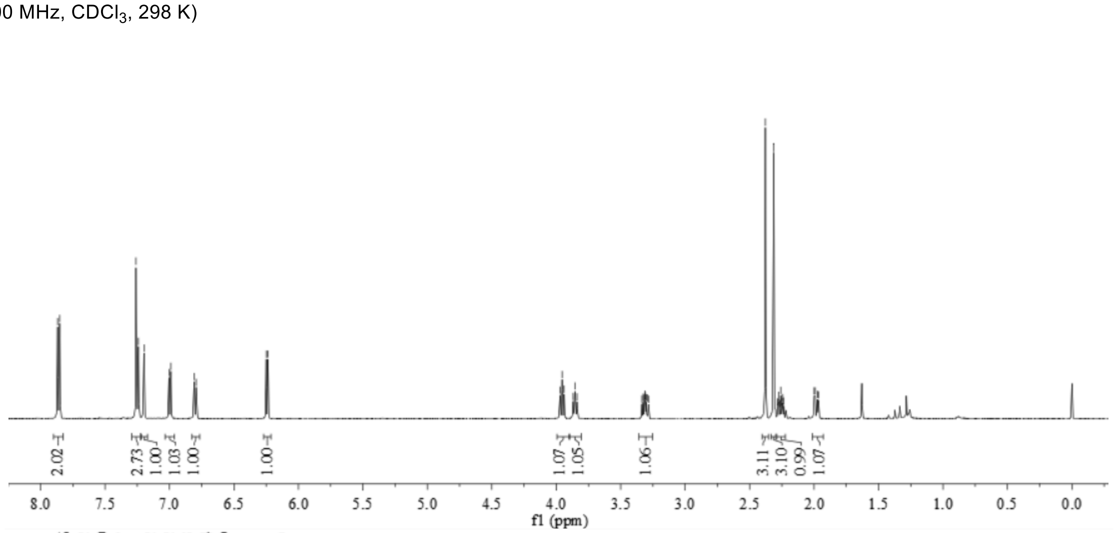

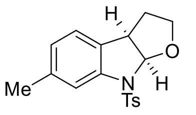

3ja
^{13}C NMR (126 MHz, $CDCl_3$, 298 K)

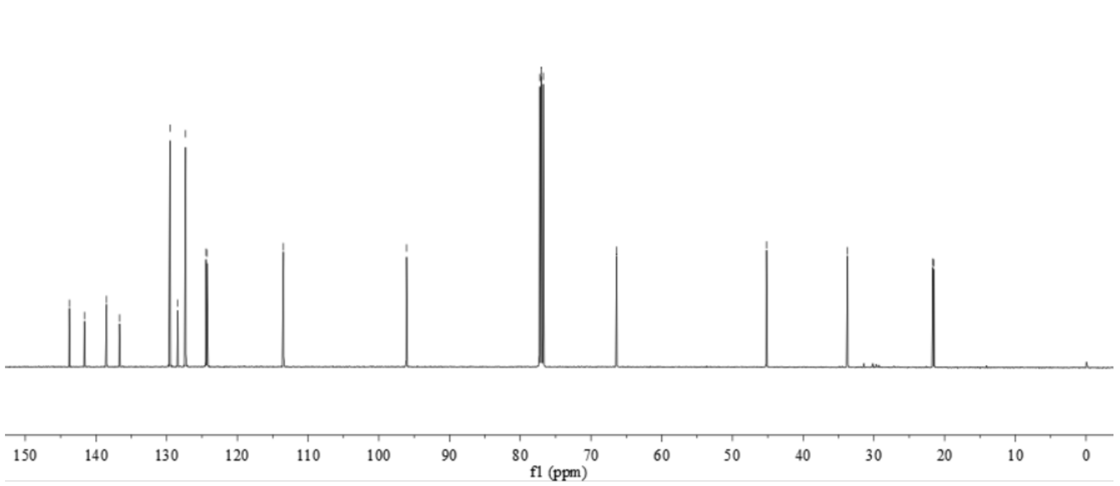

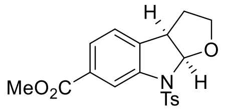

3ka

^{1}H NMR (500 MHz, $CDCl_3$, 298 K)

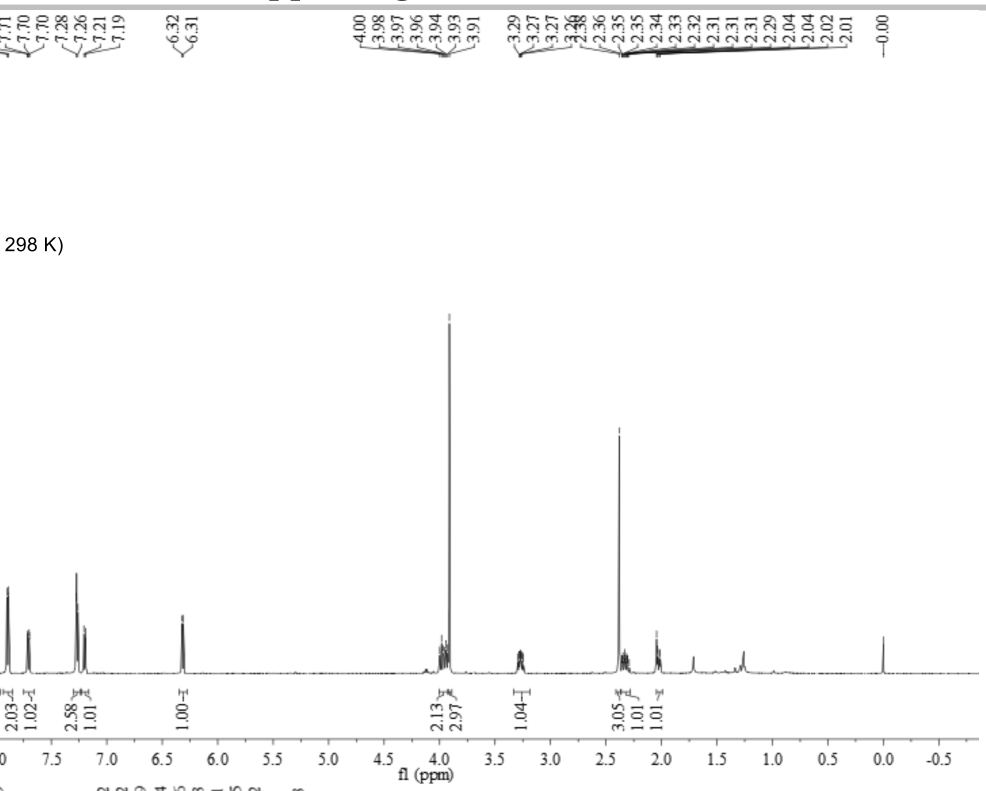

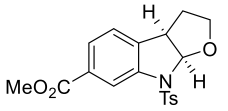

3ka

^{13}C NMR (126 MHz, $CDCl_3$, 298 K)

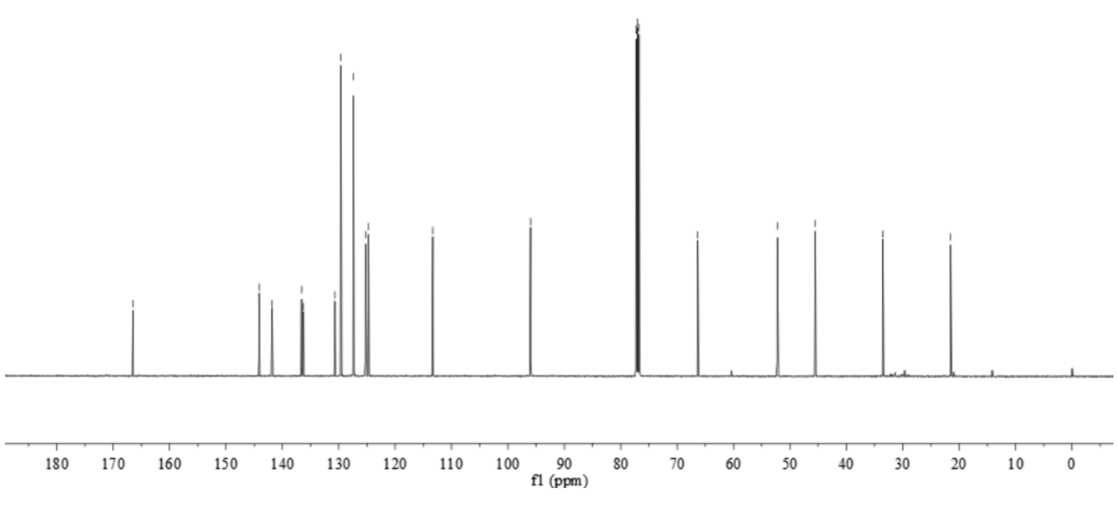

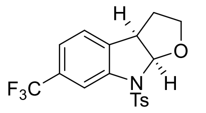

3la

^{1}H NMR (500 MHz, $CDCl_3$, 298 K)

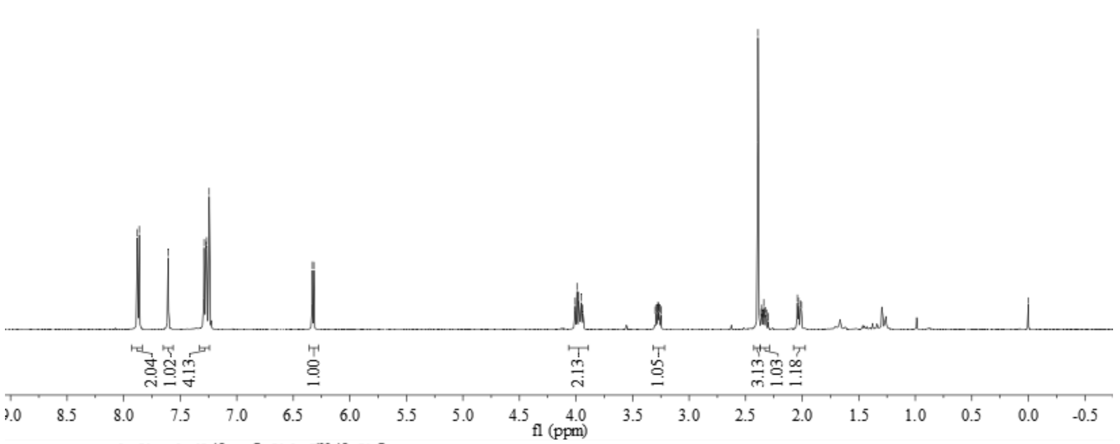

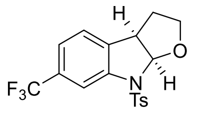

3la

^{13}C NMR (126 MHz, $CDCl_3$, 298 K)

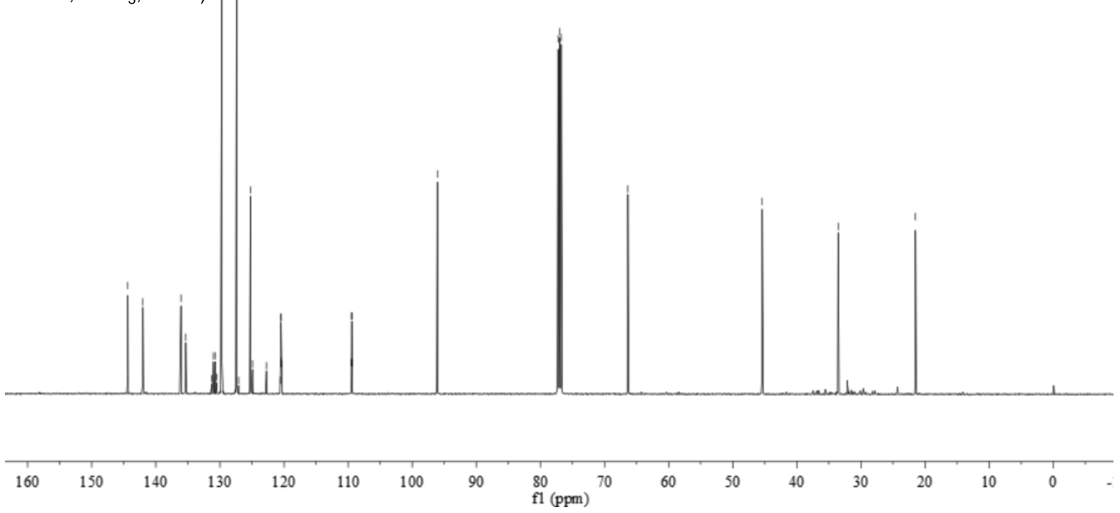

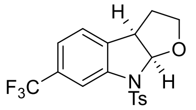

3la

^{19}F NMR (376 MHz, $CDCl_3$, 298 K)

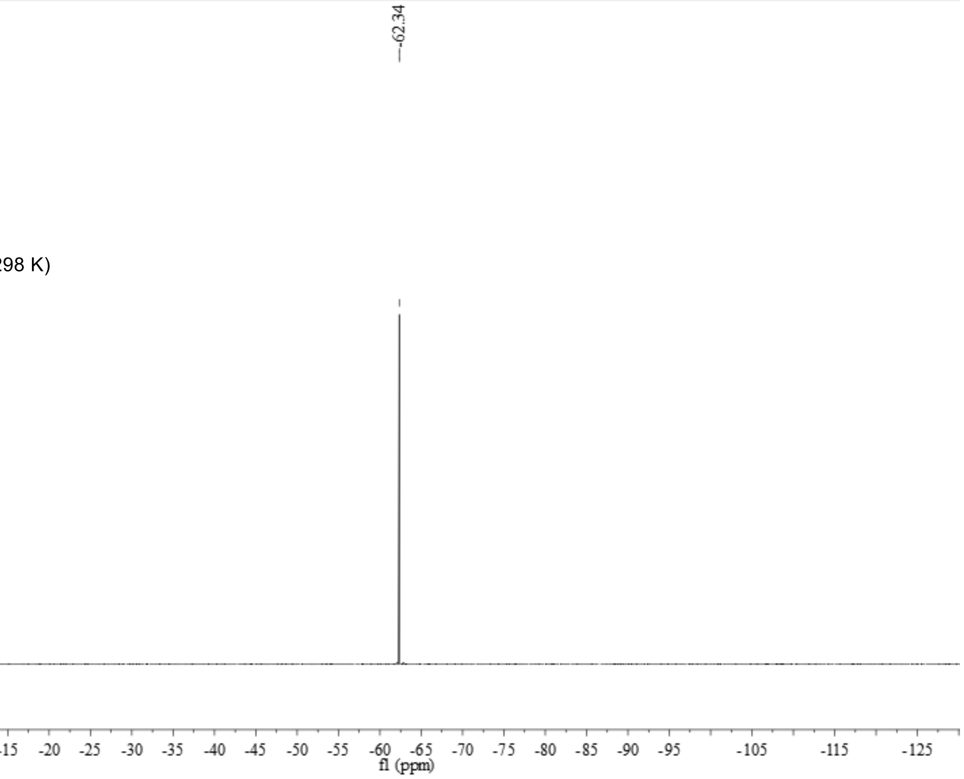

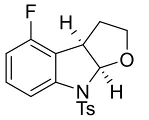

3ma
^{1}H NMR (500 MHz, $CDCl_3$, 298 K)

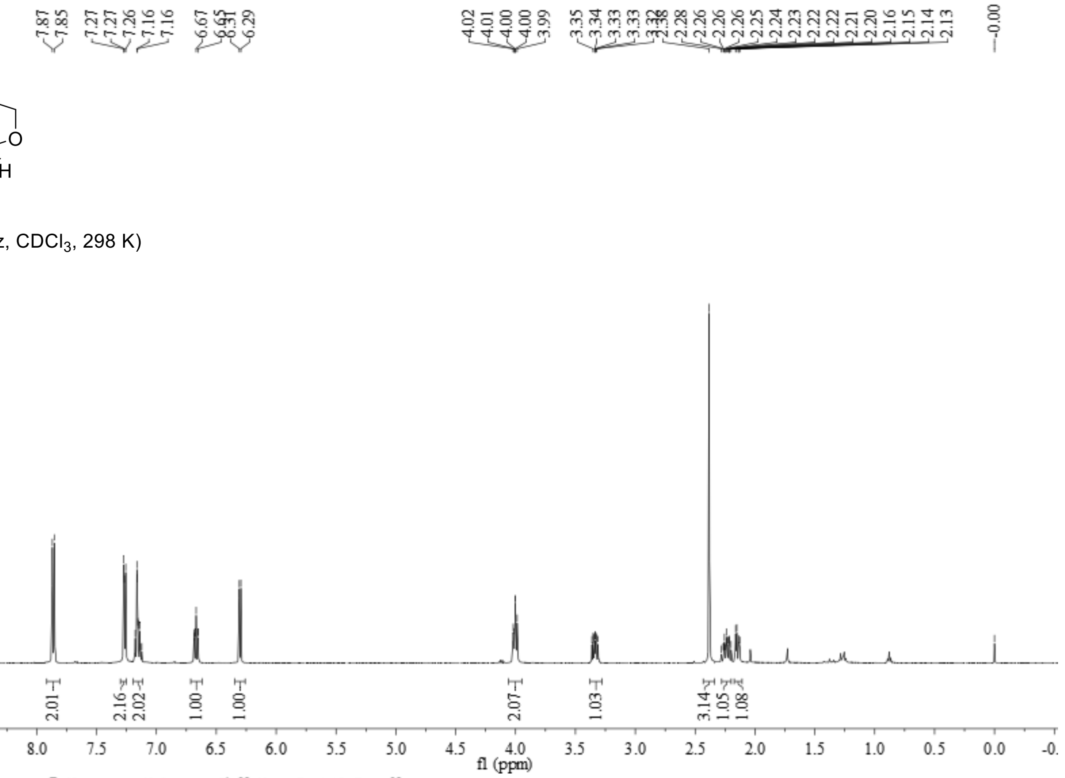

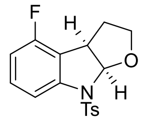

3ma
^{13}C NMR (126 MHz, $CDCl_3$, 298 K)

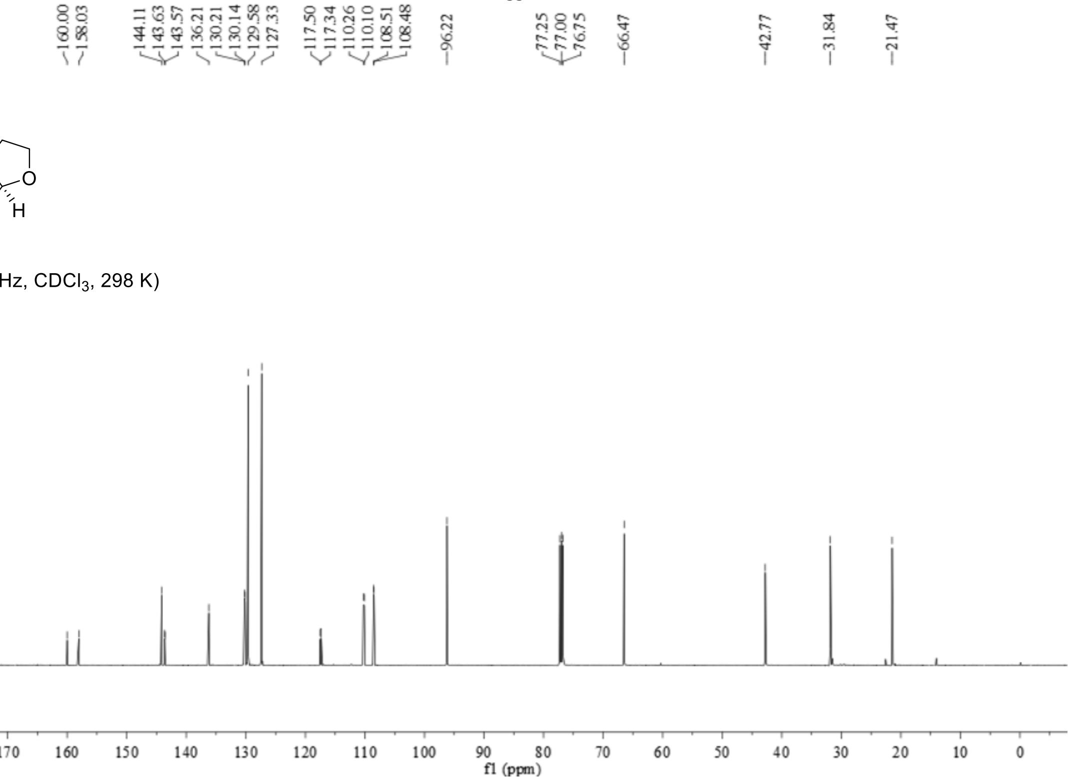

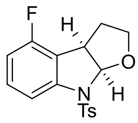

3ma

^{19}F NMR (376 MHz, $CDCl_3$, 298 K)

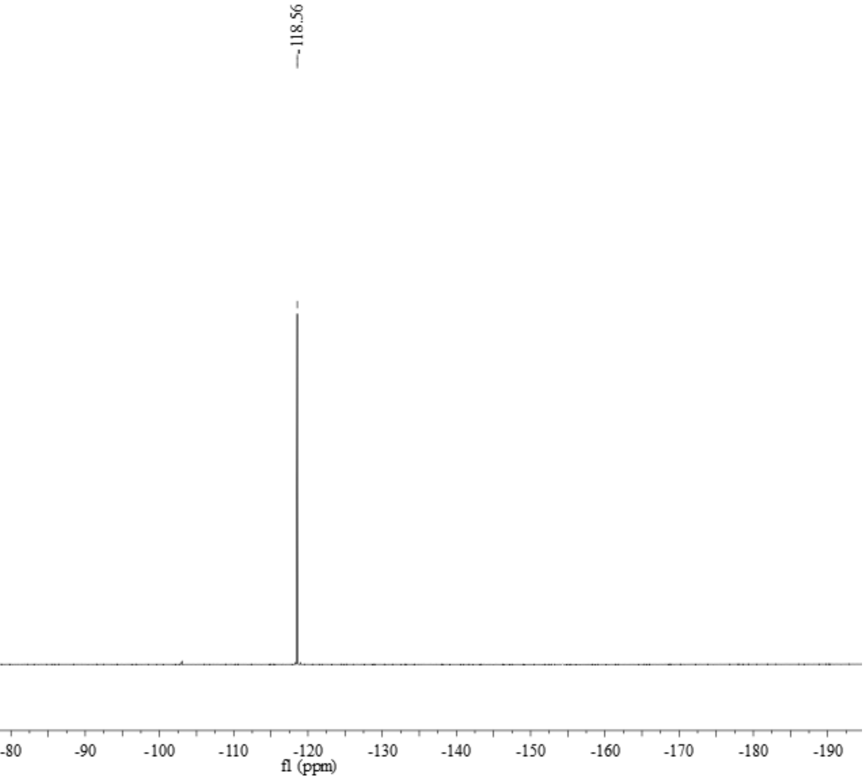

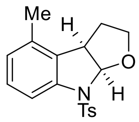

3na

^{1}H NMR (500 MHz, $CDCl_3$, 298 K)

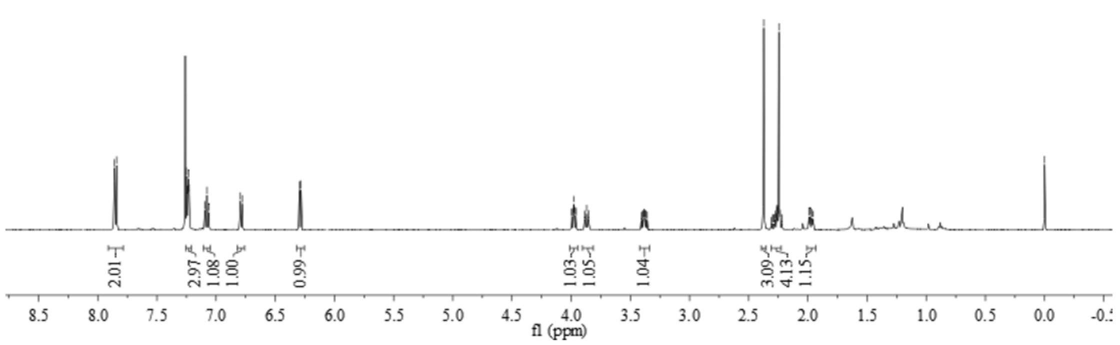

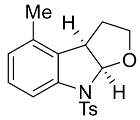

3na

^{13}C NMR (126 MHz, $CDCl_3$, 298 K)

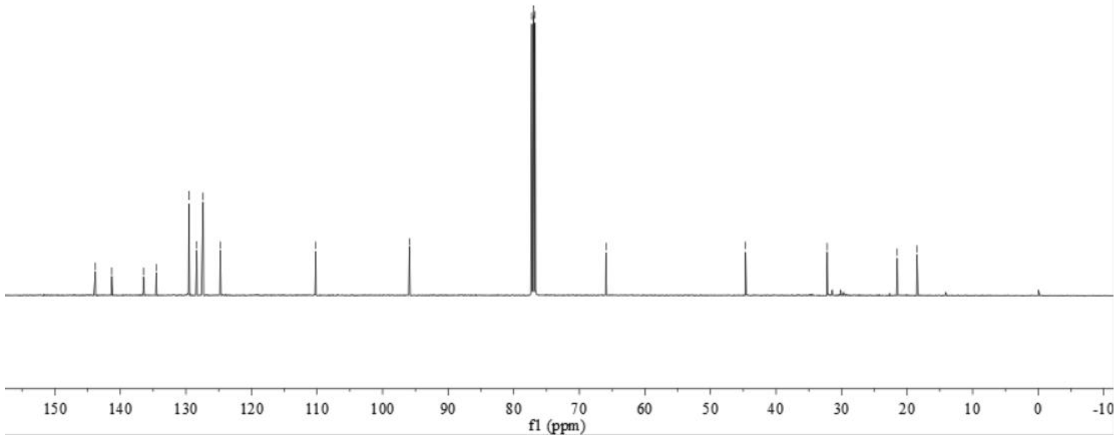

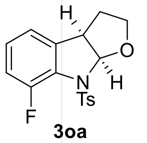

3oa

^{1}H NMR (500 MHz, $CDCl_3$, 298 K)

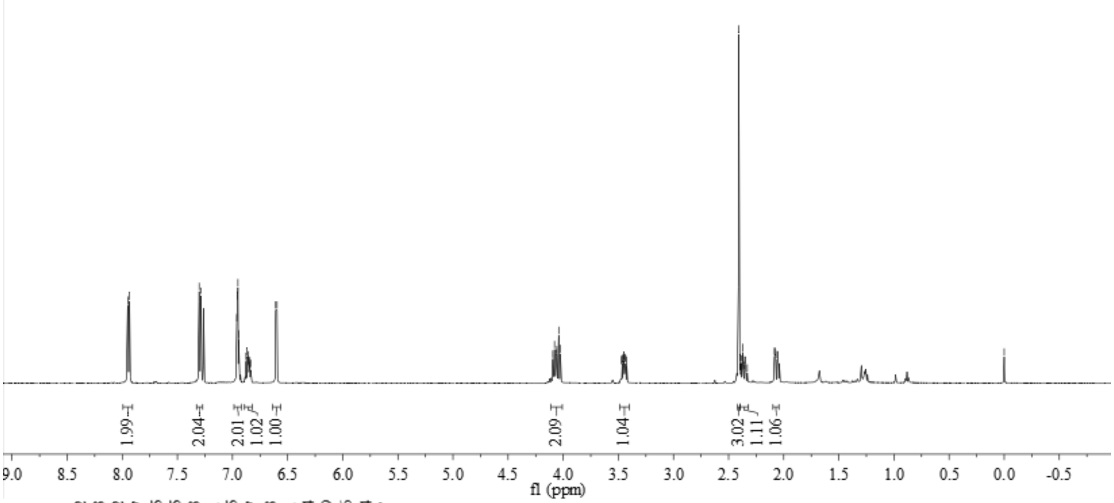

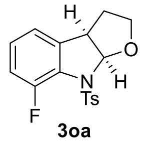

3oa

^{13}C NMR (126 MHz, $CDCl_3$, 298 K)

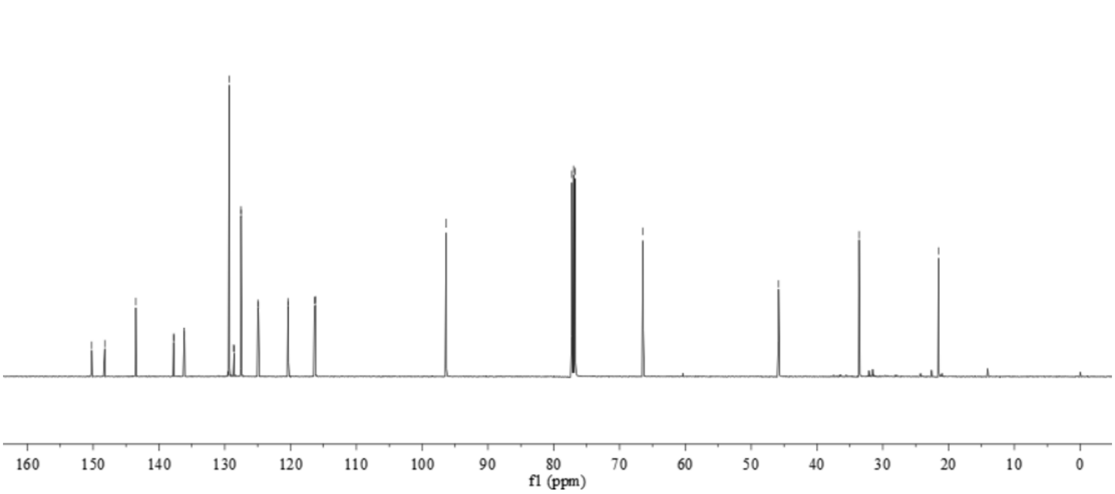

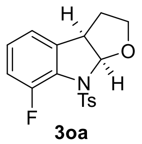

3oa

^{19}F NMR (376 MHz, $CDCl_3$, 298 K)

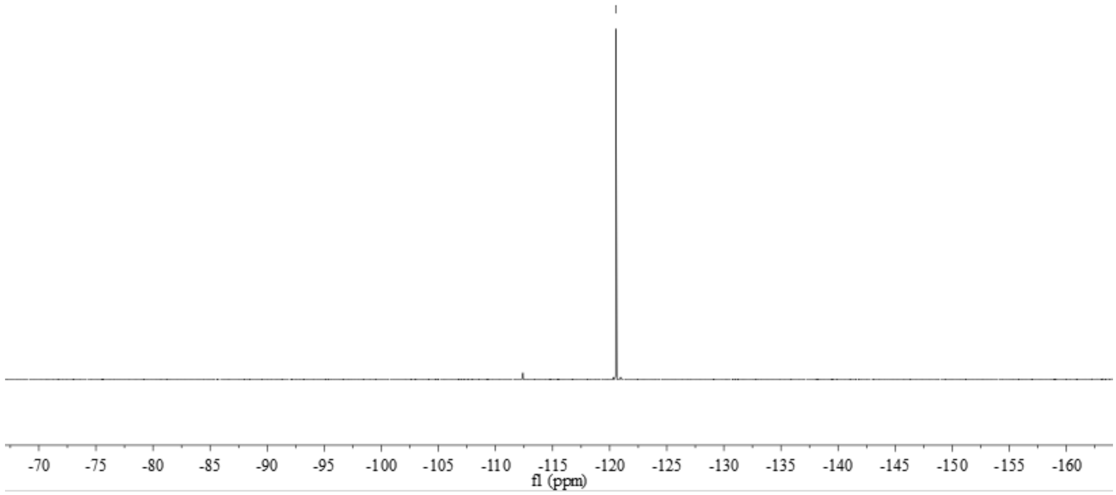

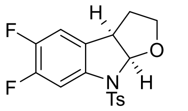

3pa

^{1}H NMR (500 MHz, $CDCl_3$, 298 K)

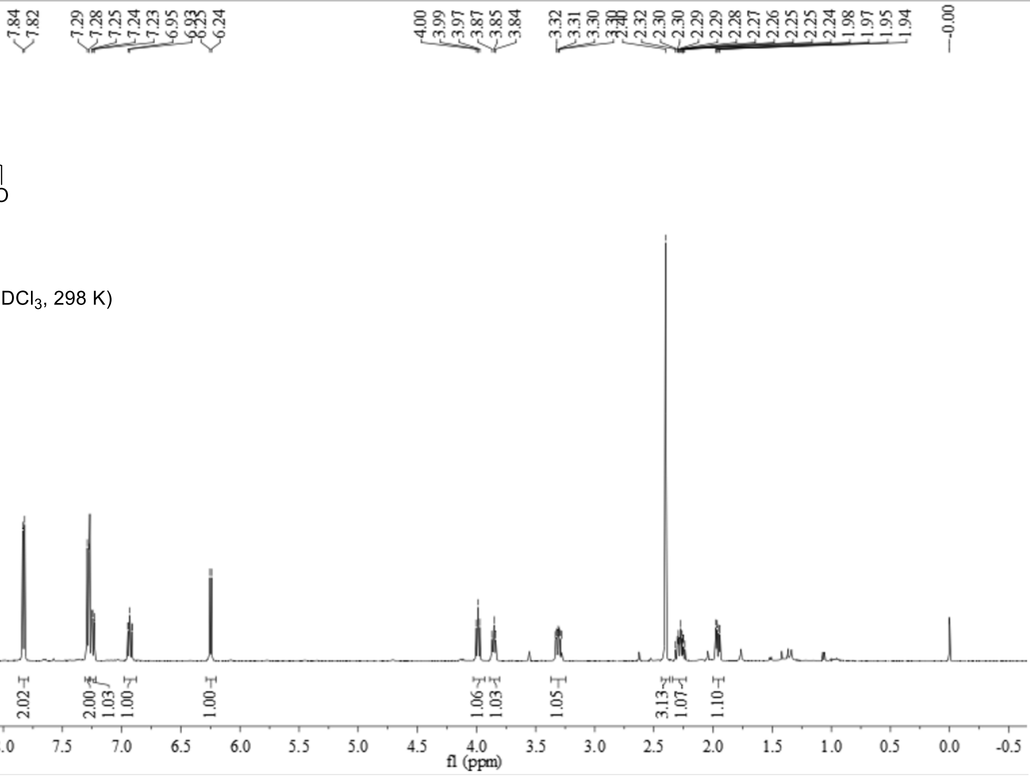

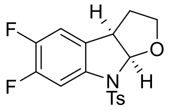

3pa

^{13}C NMR (126 MHz, $CDCl_3$, 298 K)

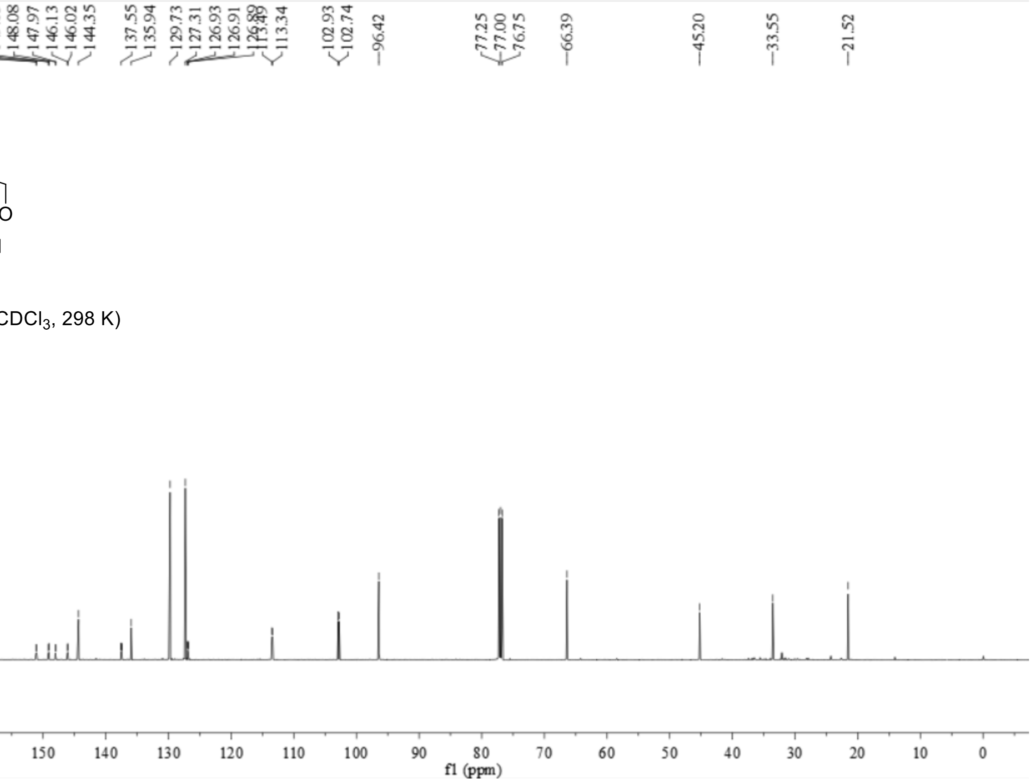

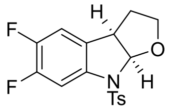

3pa

^{19}F NMR (376 MHz, $CDCl_3$, 298 K)

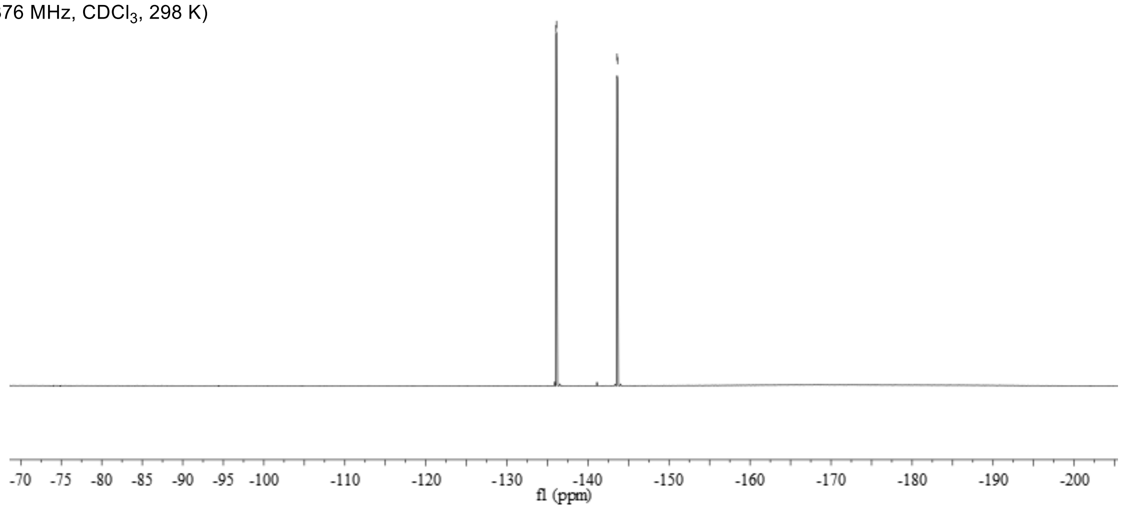

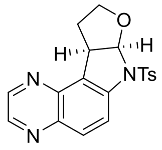

3qa

^{1}H NMR (500 MHz, $CDCl_3$, 298 K)

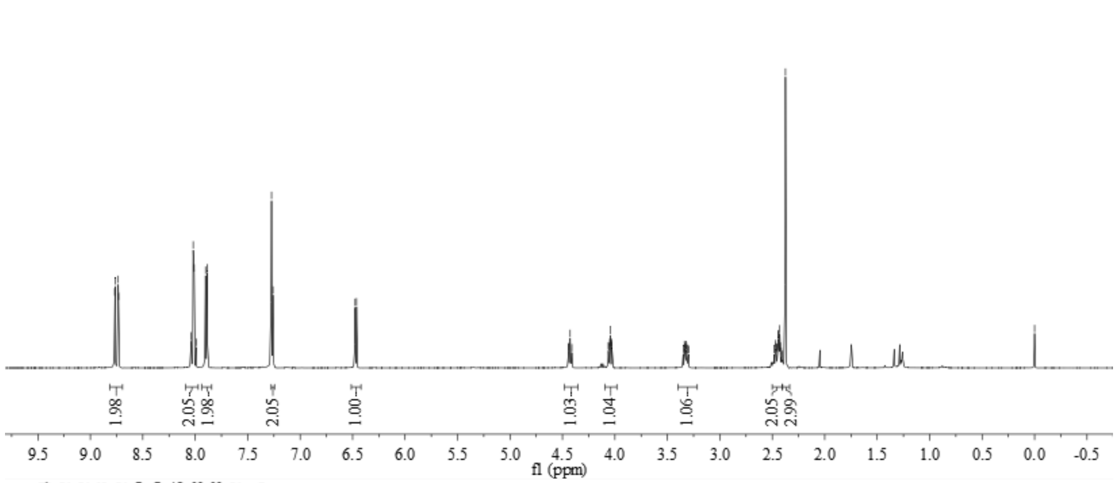

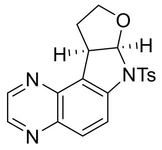

3qa

^{13}C NMR (126 MHz, $CDCl_3$, 298 K)

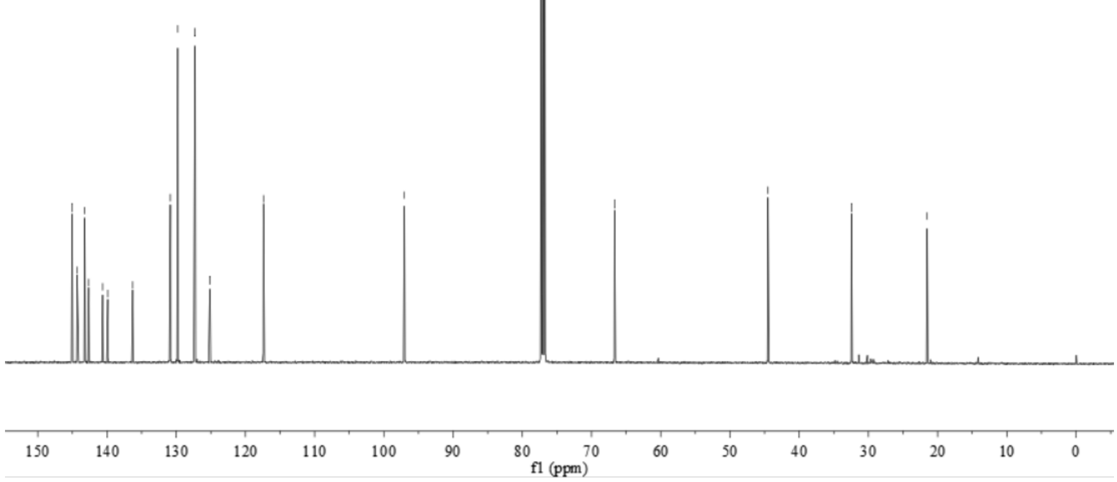

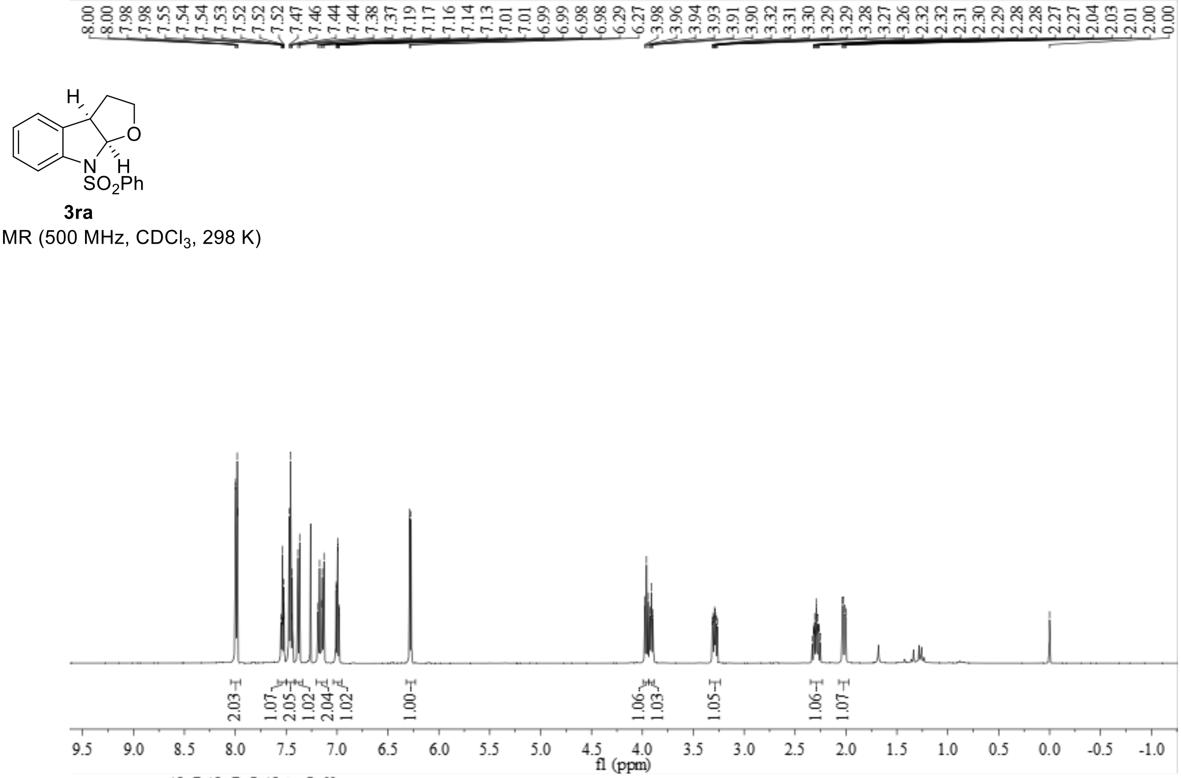

3ra

^{1}H NMR (500 MHz, $CDCl_3$, 298 K)

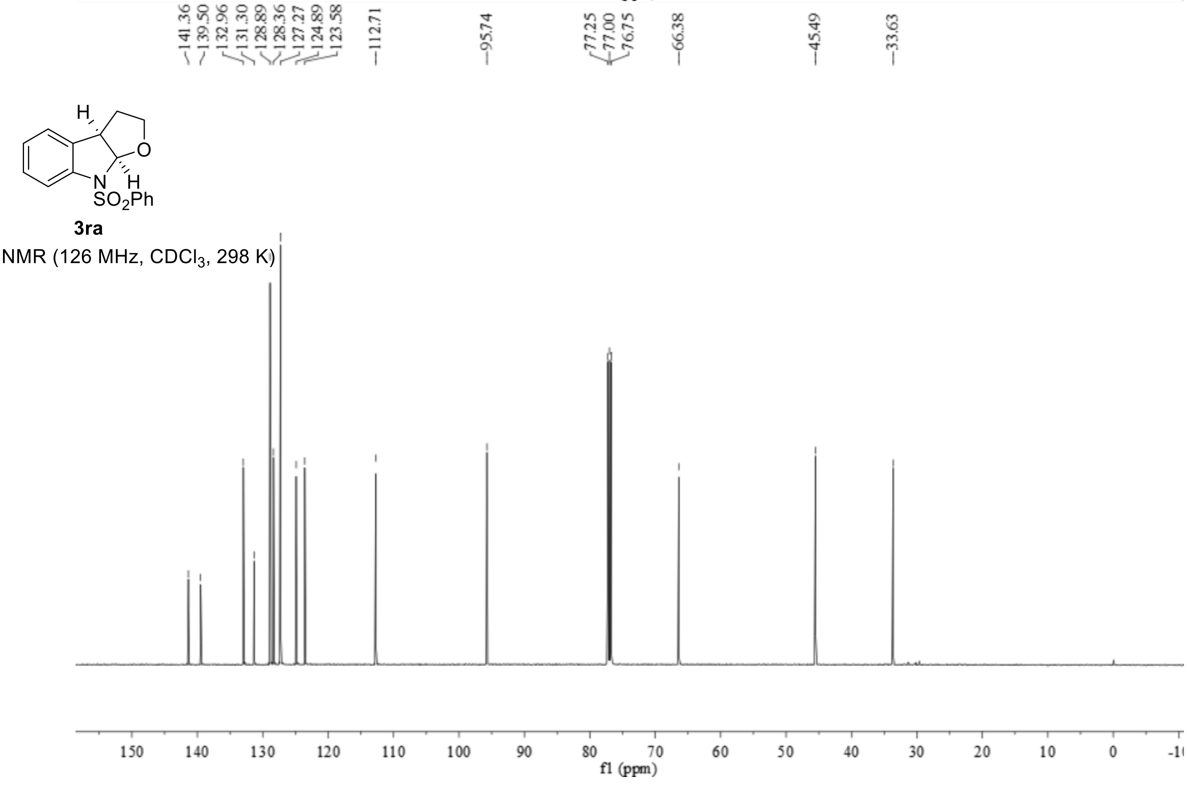

3ra

^{13}C NMR (126 MHz, $CDCl_3$, 298 K)

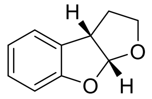

6aa

^{1}H NMR (500 MHz, $CDCl_3$, 298 K)

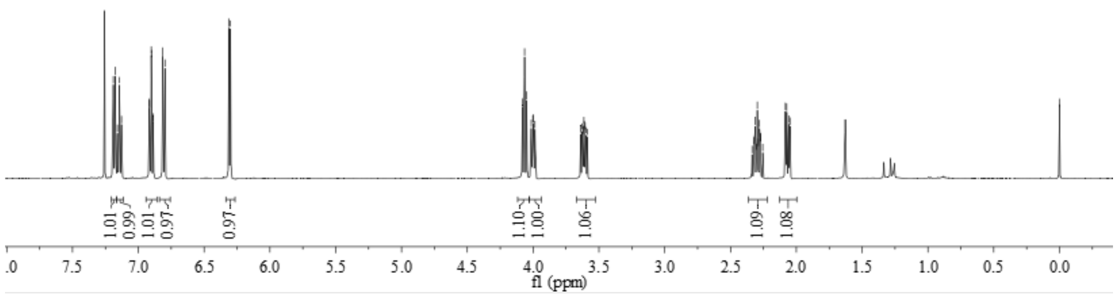

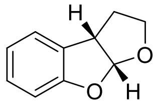

6aa

^{13}C NMR (126 MHz, $CDCl_3$, 298 K)

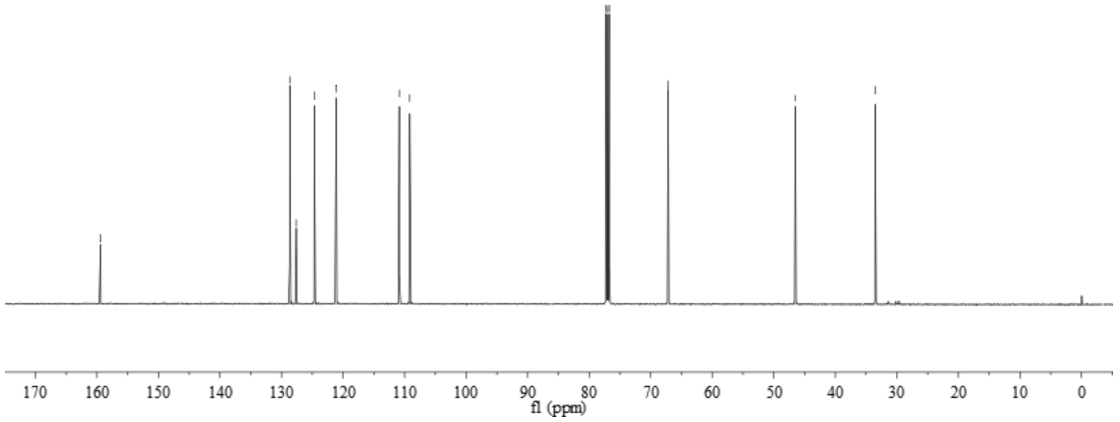

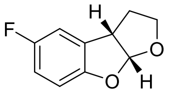

6ba

^{1}H NMR (500 MHz, $CDCl_3$, 298 K)

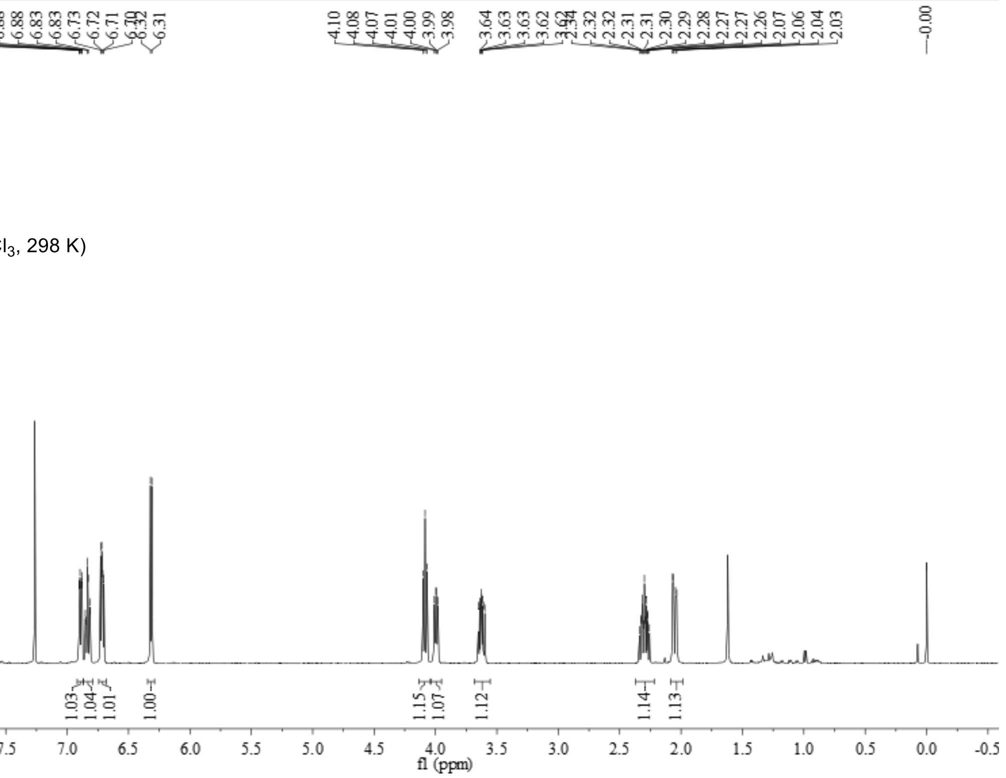

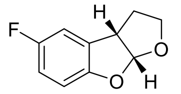

6ba

^{13}C NMR (126 MHz, $CDCl_3$, 298 K)

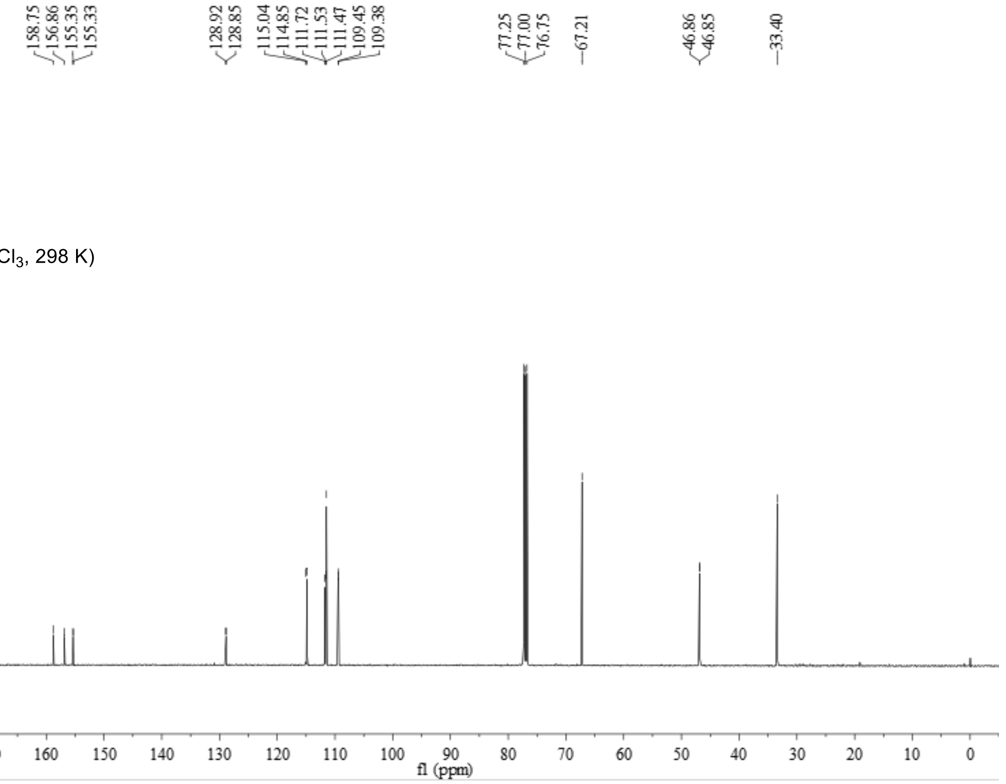

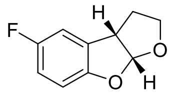

6ba

^{19}F NMR (376 MHz, $CDCl_3$, 298 K)

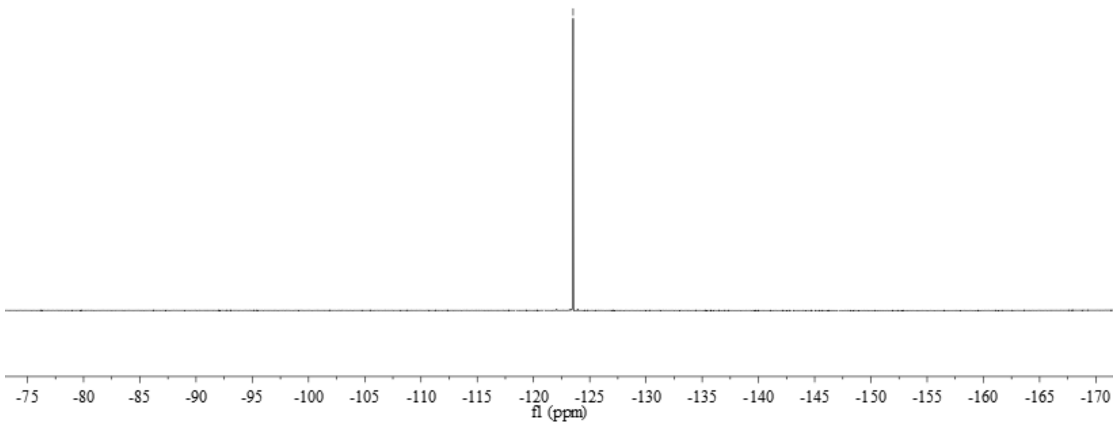

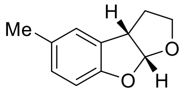

6ca

^{1}H NMR (500 MHz, $CDCl_3$, 298 K)

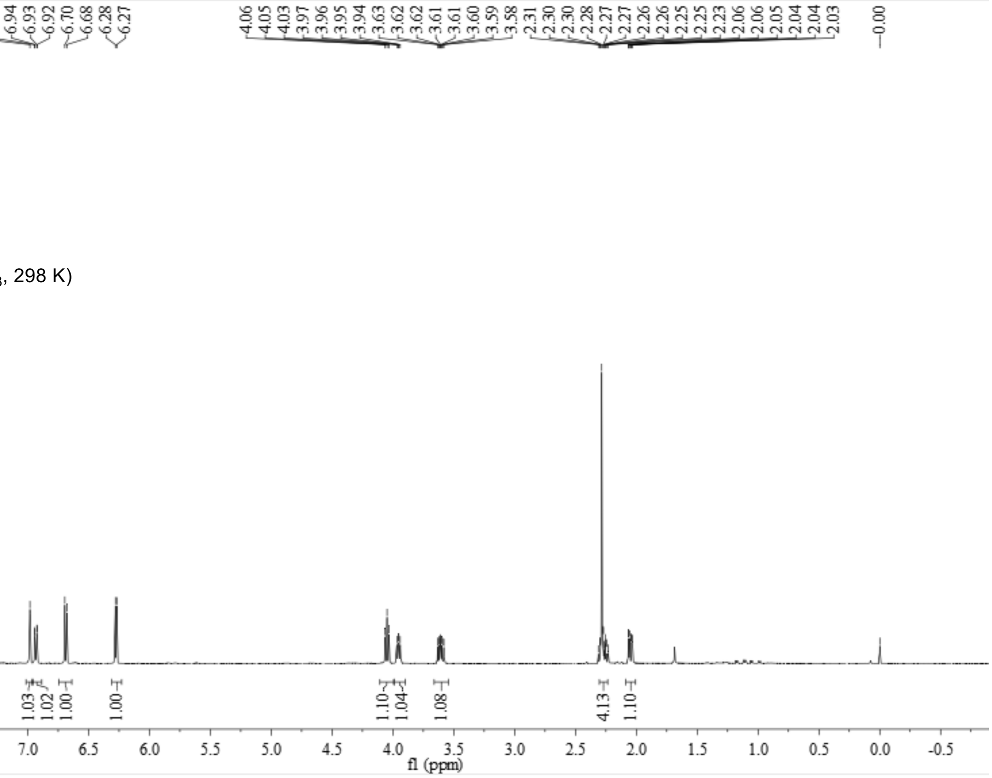

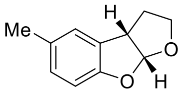

6ca

^{13}C NMR (126 MHz, $CDCl_3$, 298 K)

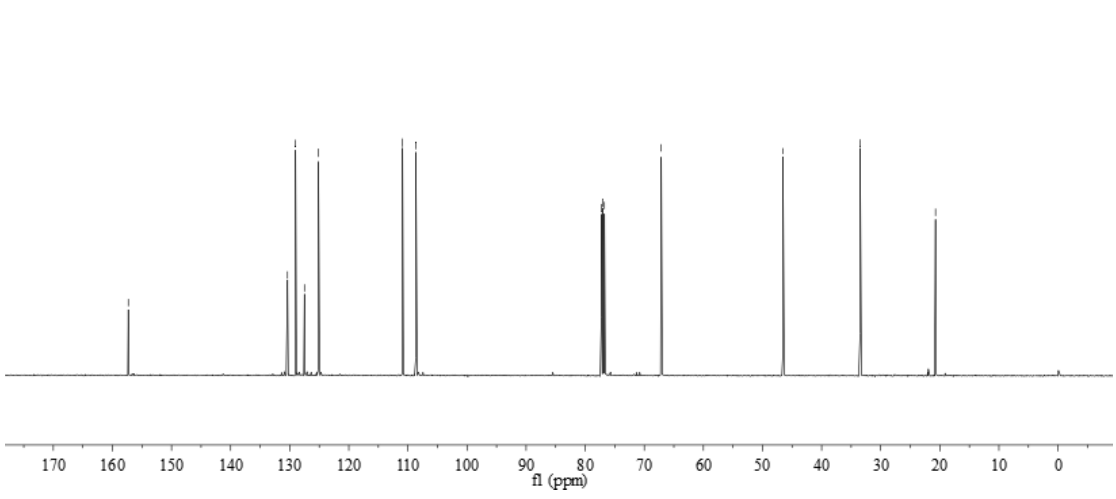

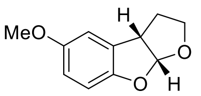

6da

1H NMR (500 MHz, $CDCl_3$, 298 K)

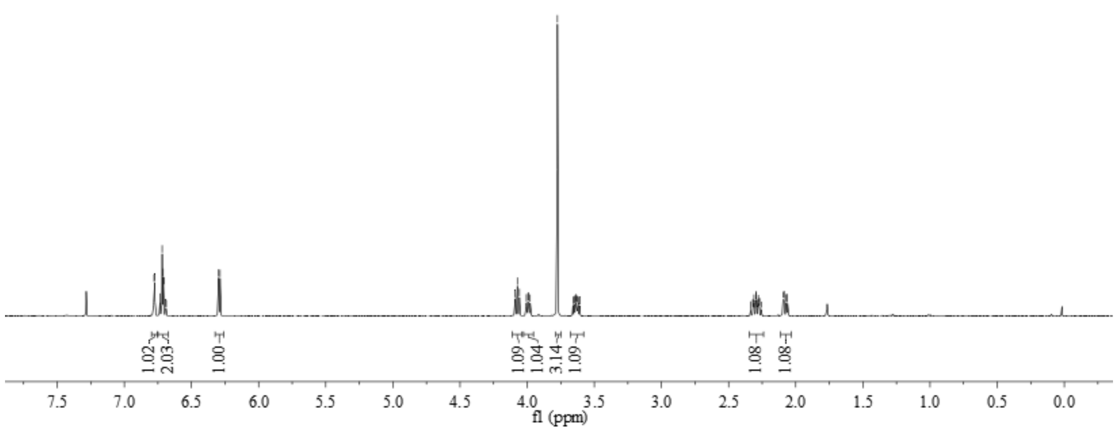

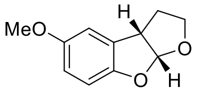

6da

13C NMR (126 MHz, $CDCl_3$, 298 K)

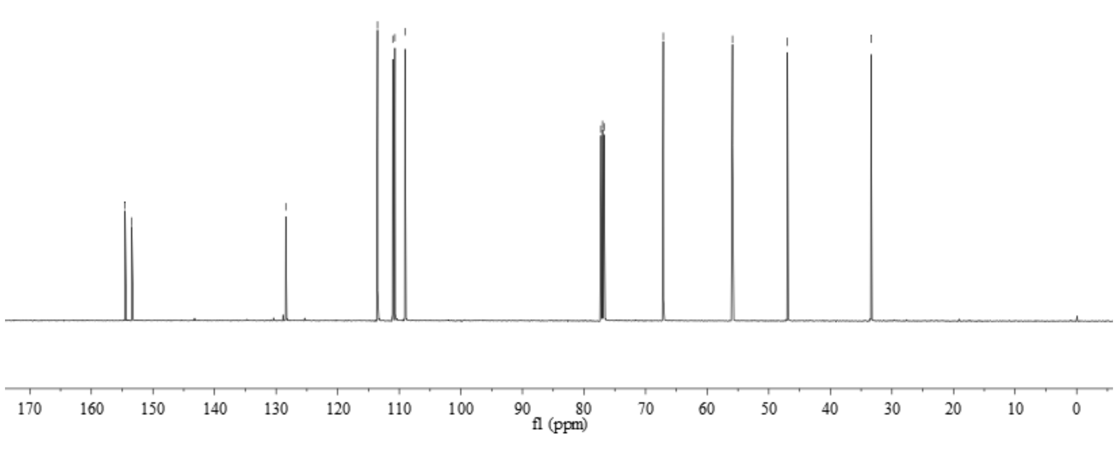

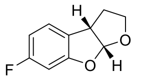

6ea

^{1}H NMR (500 MHz, CDCl$_3$, 298 K)

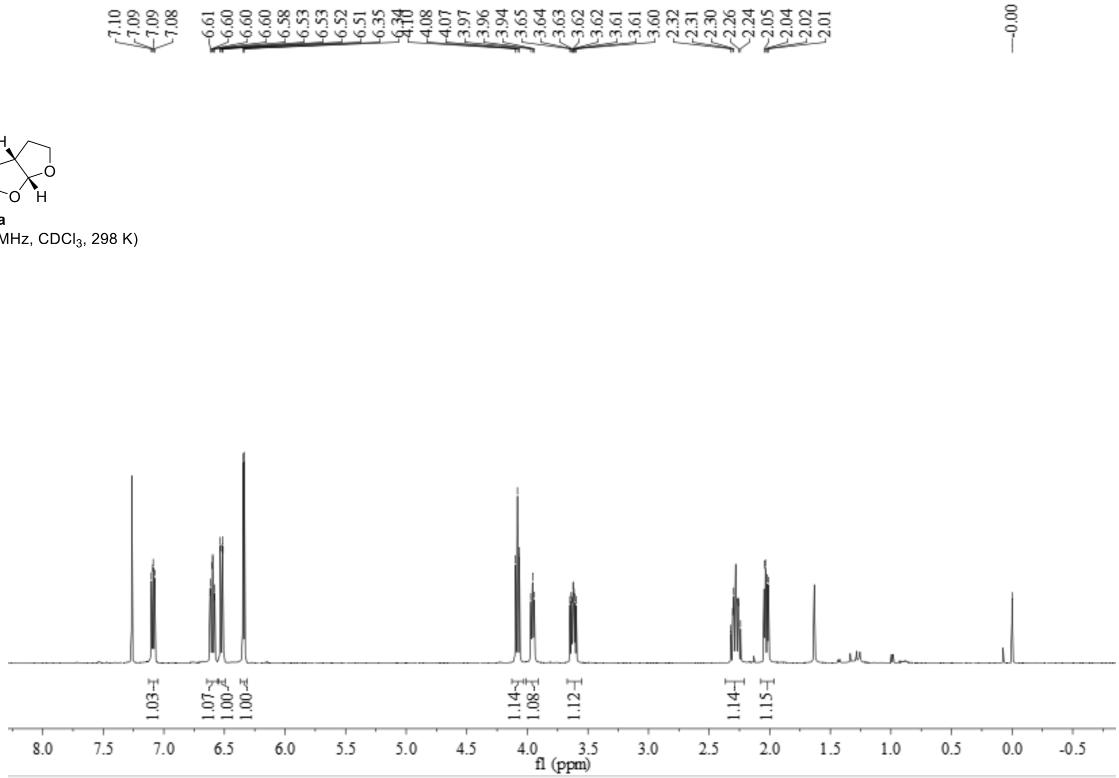

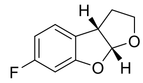

6ea

^{13}C NMR (126 MHz, CDCl$_3$, 298 K)

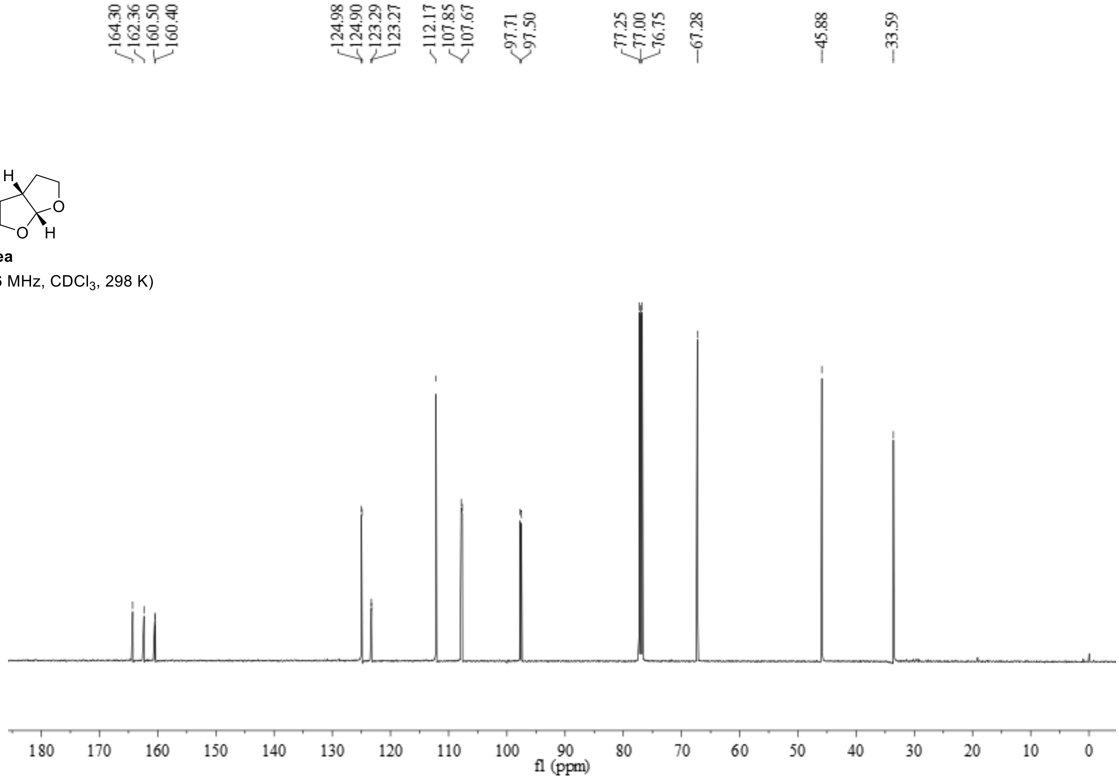

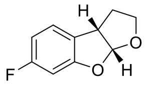

6ea

^{19}F NMR (376 MHz, $CDCl_3$, 298 K)

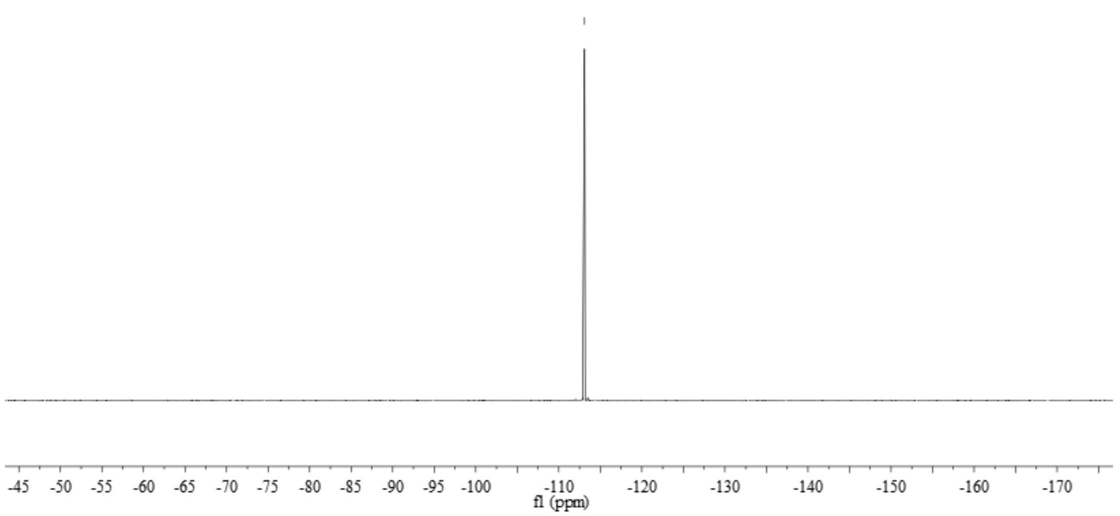

6fa

^{1}H NMR (500 MHz, $CDCl_3$, 298 K)

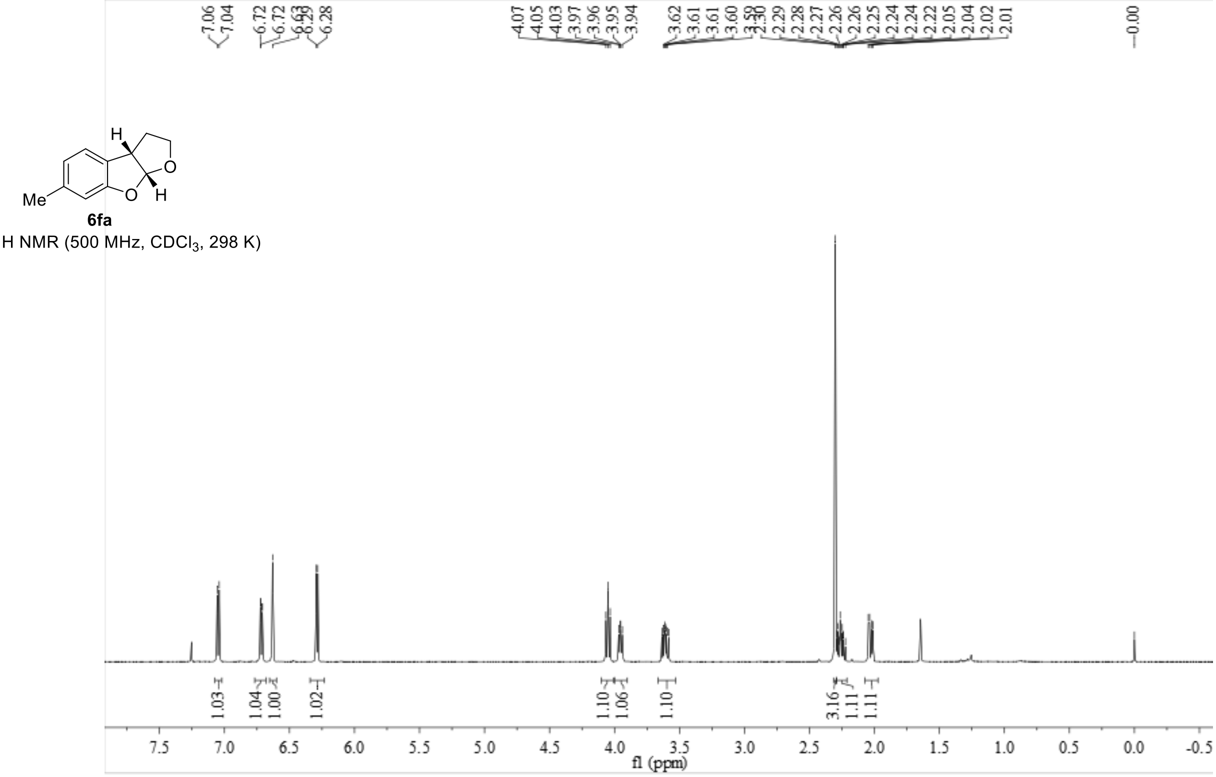

6fa

^{13}C NMR (126 MHz, $CDCl_3$, 298 K)

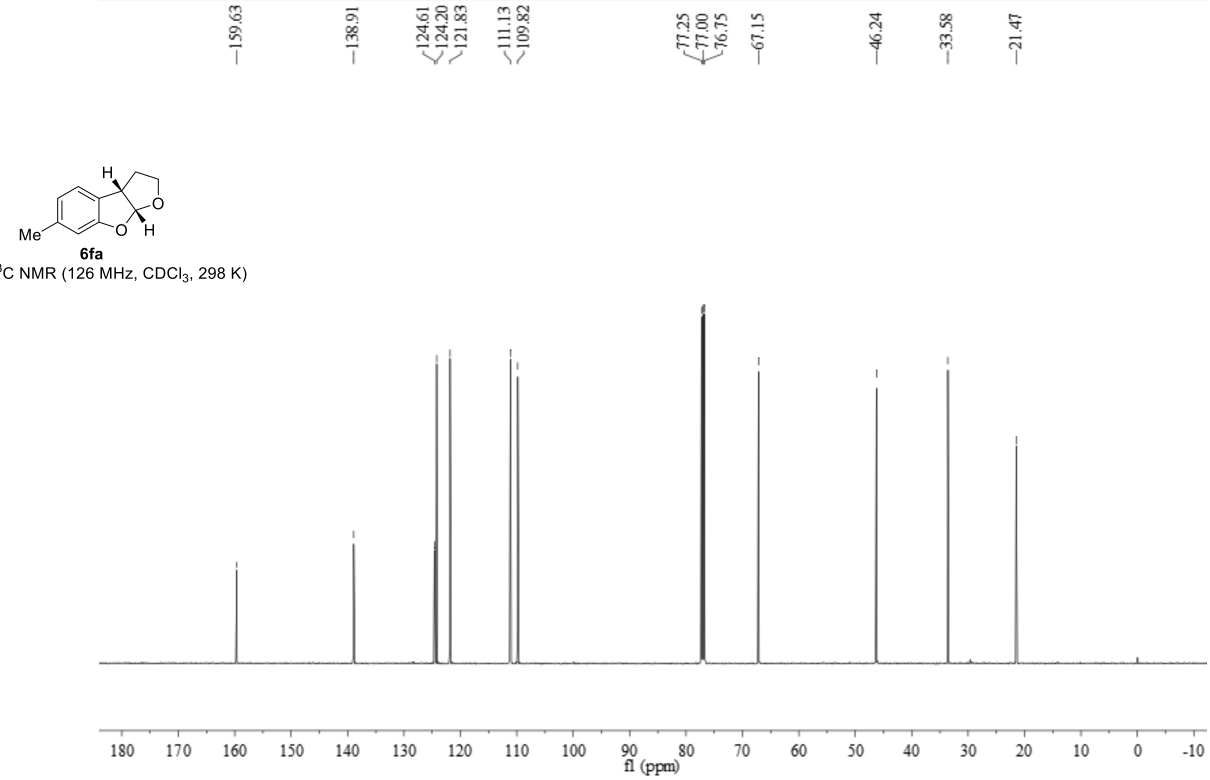

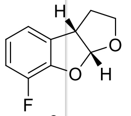

6ga

^{1}H NMR (500 MHz, $CDCl_3$, 298 K)

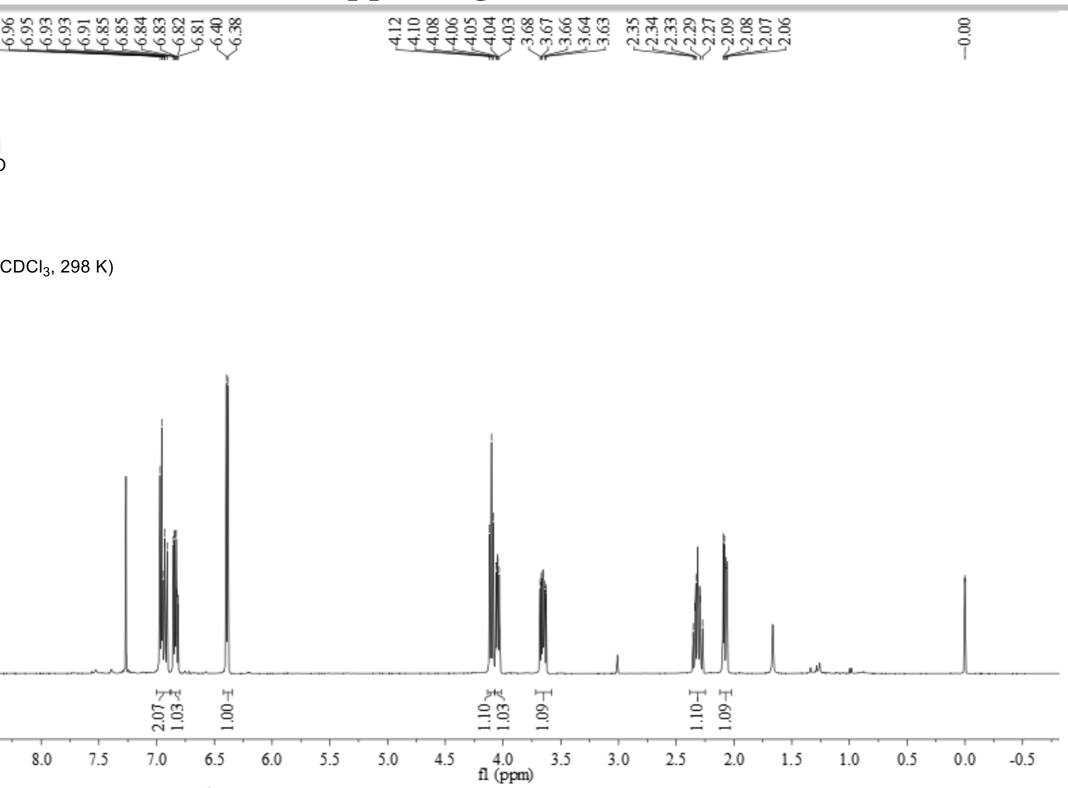

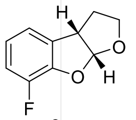

6ga

^{13}C NMR (126 MHz, $CDCl_3$, 298 K)

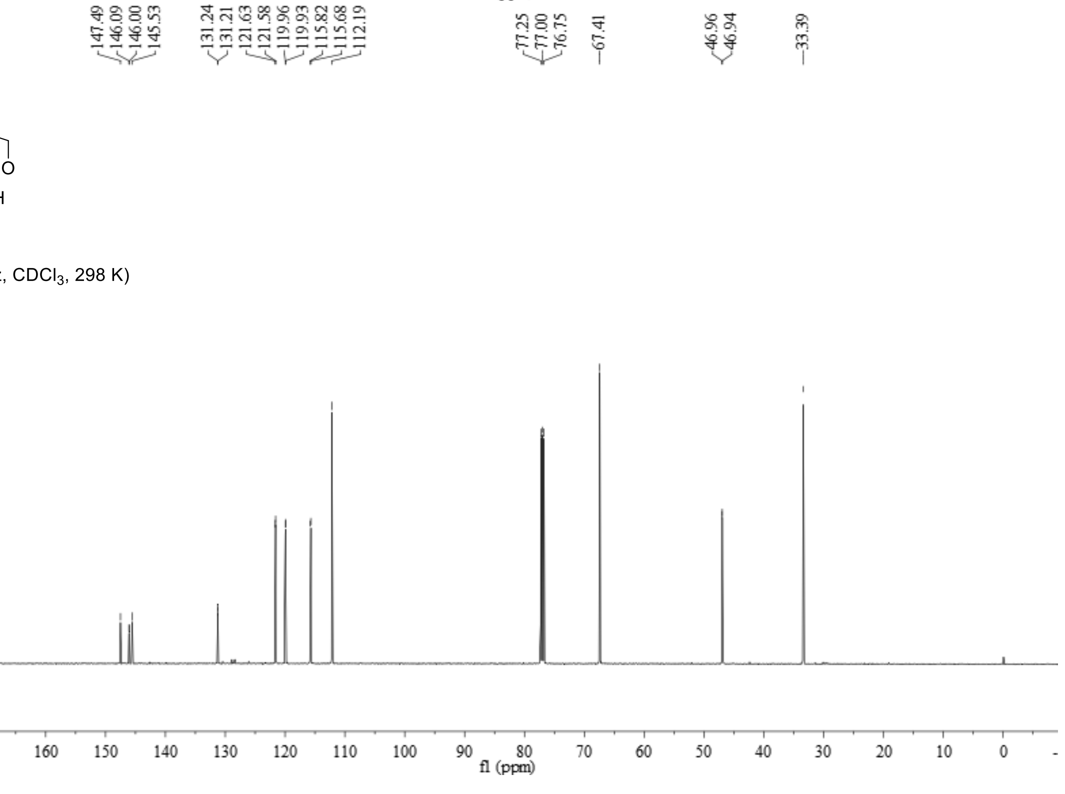

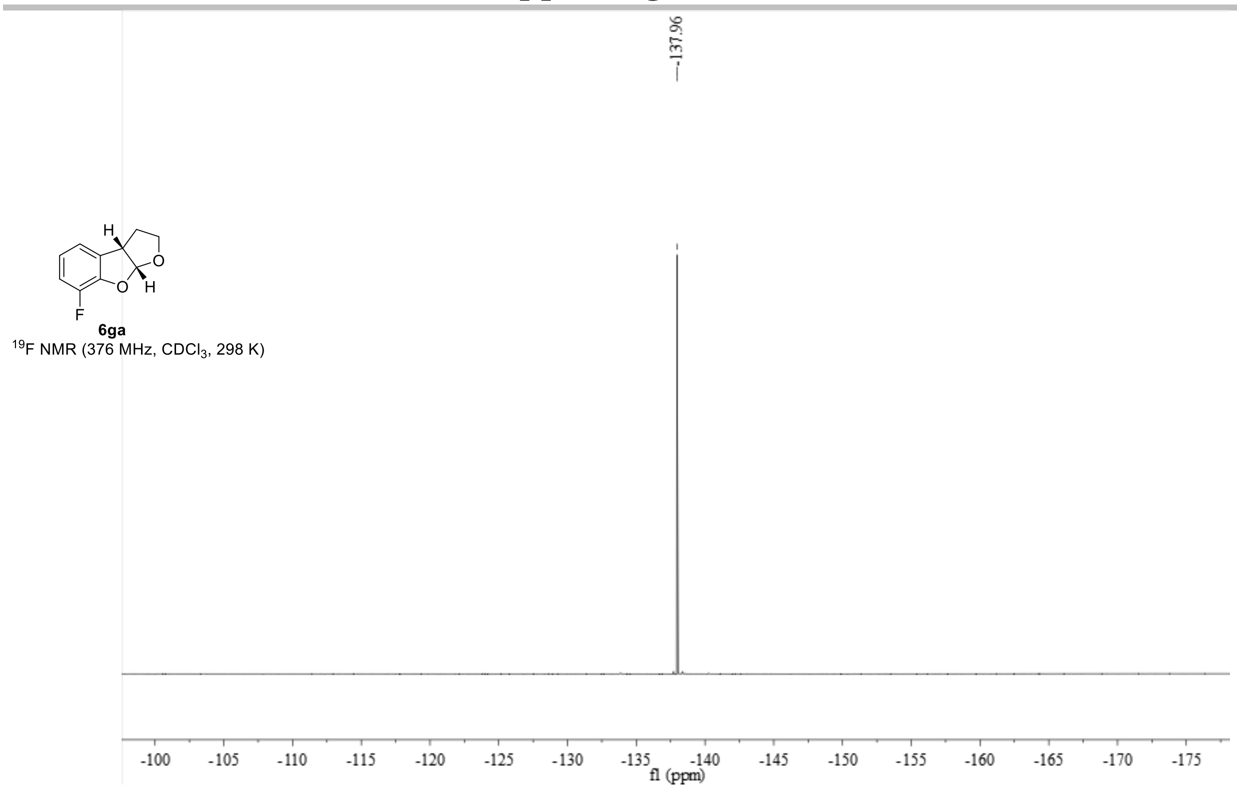
-137.96
F
O
O
H
H
6ga
19F NMR (376 MHz, CDCl3, 298 K)
-100 -105 -110 -115 -120 -125 -130 -135 -140 -145 -150 -155 -160 -165 -170 -175
f1 (ppm)

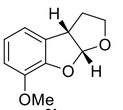

6ha

^{1}H NMR (500 MHz, $CDCl_3$, 298 K)

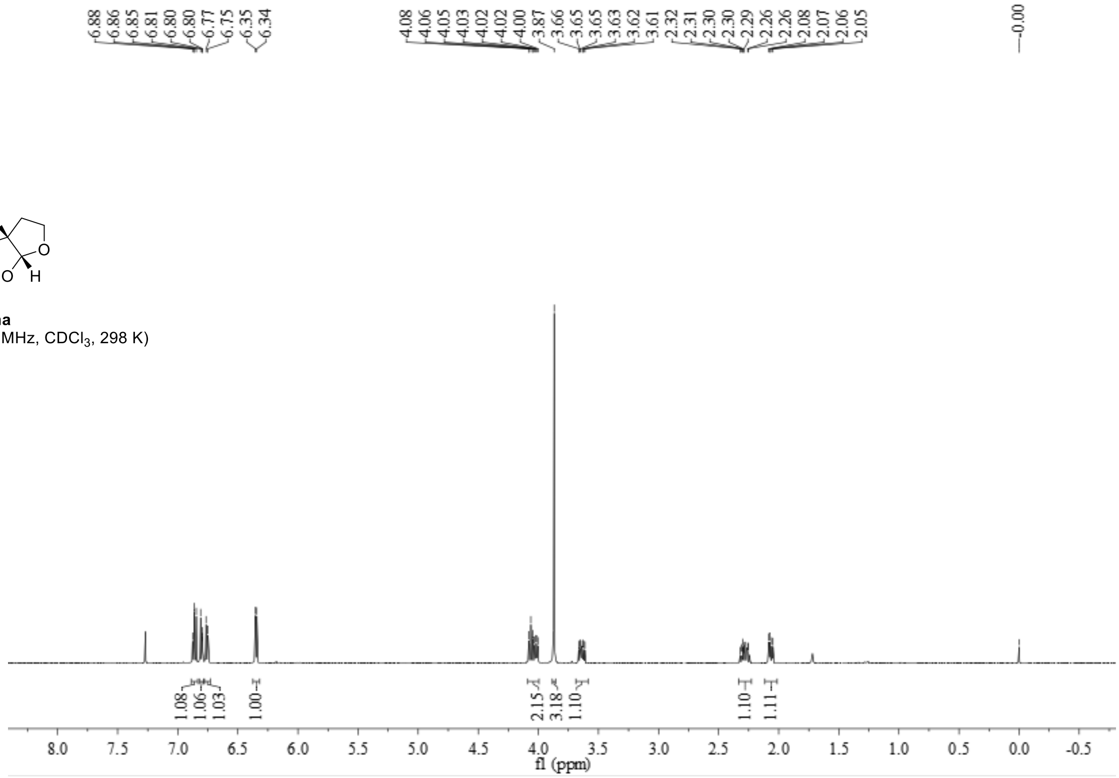

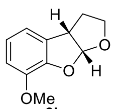

6ha

^{13}C NMR (126 MHz, $CDCl_3$, 298 K)

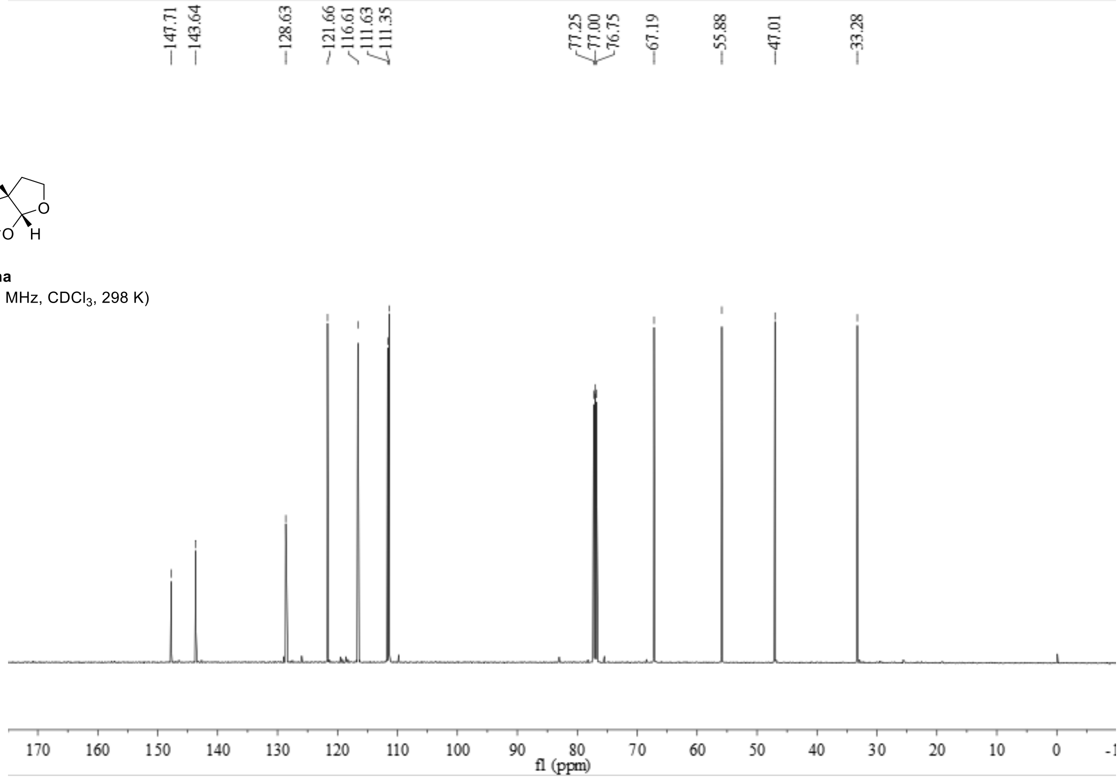

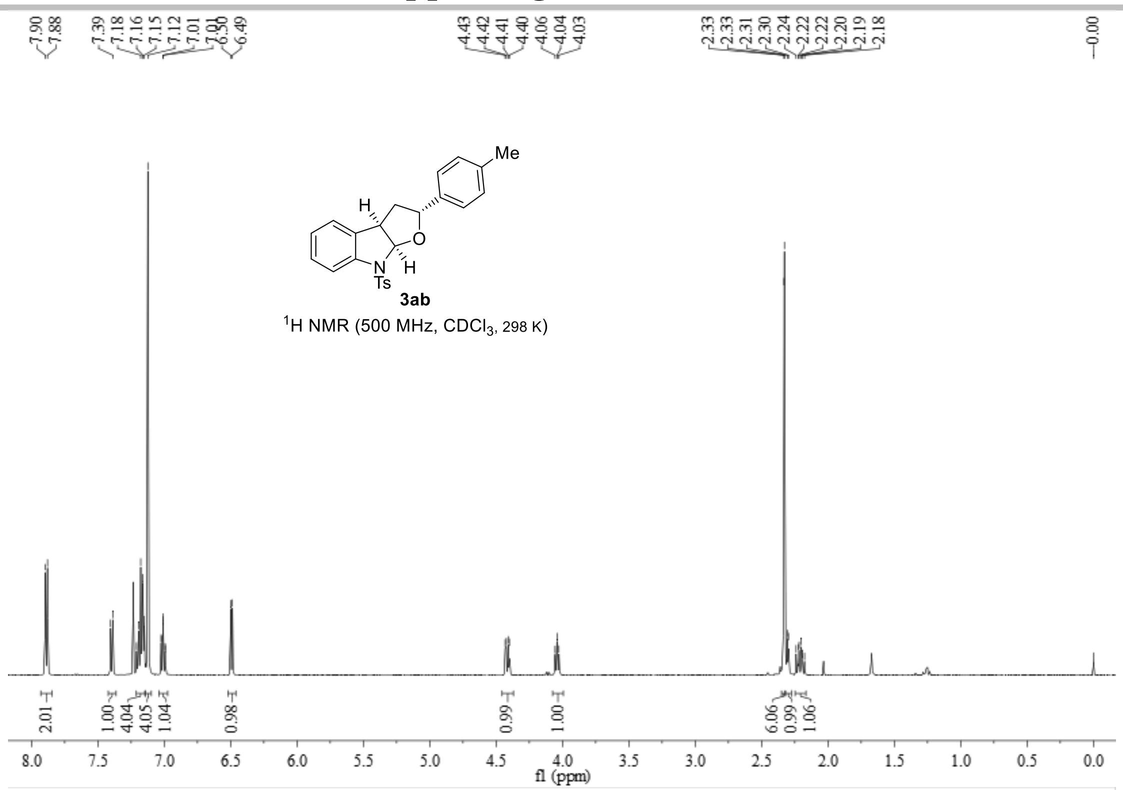
7.90
7.88
7.39
7.18
7.16
7.15
7.12
7.01
7.01
6.50
6.49
4.43
4.42
4.41
4.40
4.06
4.04
4.03
2.33
2.33
2.31
2.30
2.24
2.22
2.22
2.20
2.19
2.18
0.00
Me
H
O
N
H
Ts
3ab
1H NMR (500 MHz, CDCl3, 298 K)
2.01
1.00
4.04
4.05
1.04
0.98
0.99
1.00
6.06
0.99
1.06
fl (ppm)

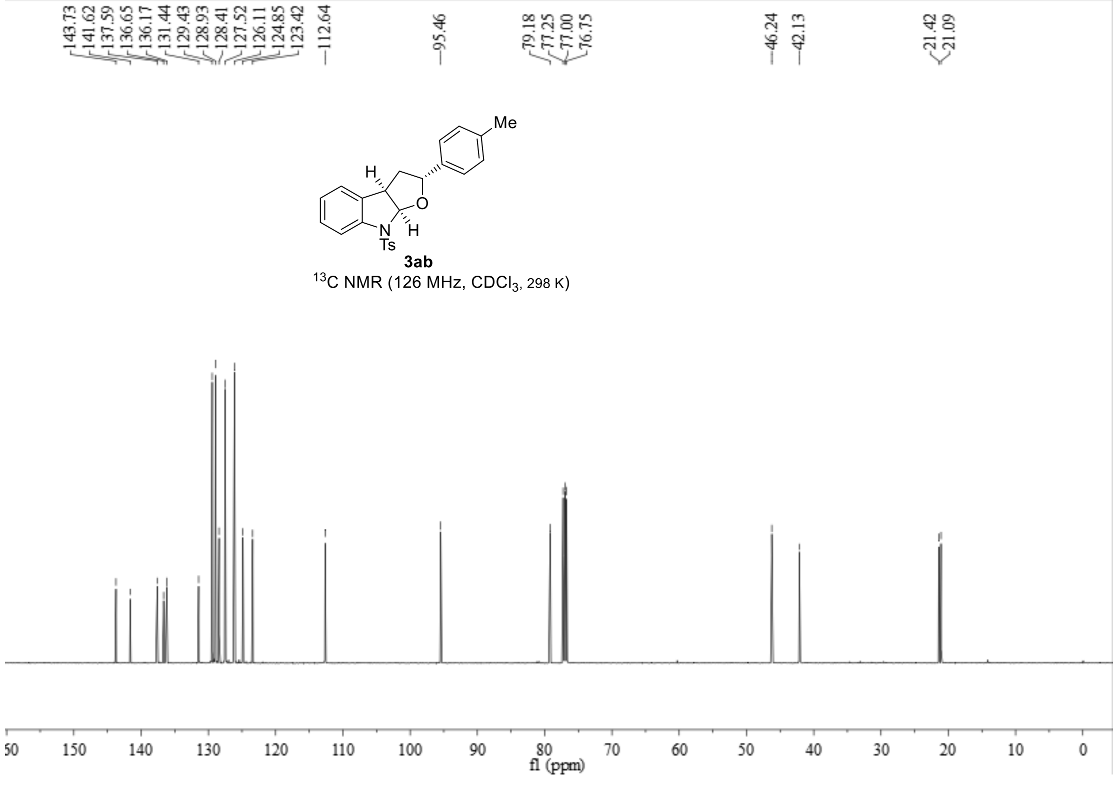
143.73
141.62
137.59
136.65
136.17
131.44
129.43
128.93
128.41
127.52
126.11
124.85
123.42
112.64
95.46
79.18
77.25
77.00
76.75
46.24
42.13
21.42
21.09
Me
H
O
N
H
Ts
3ab
13C NMR (126 MHz, CDCl3, 298 K)
fl (ppm)

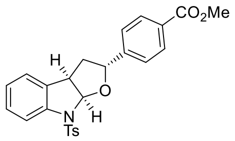

3ac

^{1}H NMR (500 MHz, $CDCl_3$, 298 K)

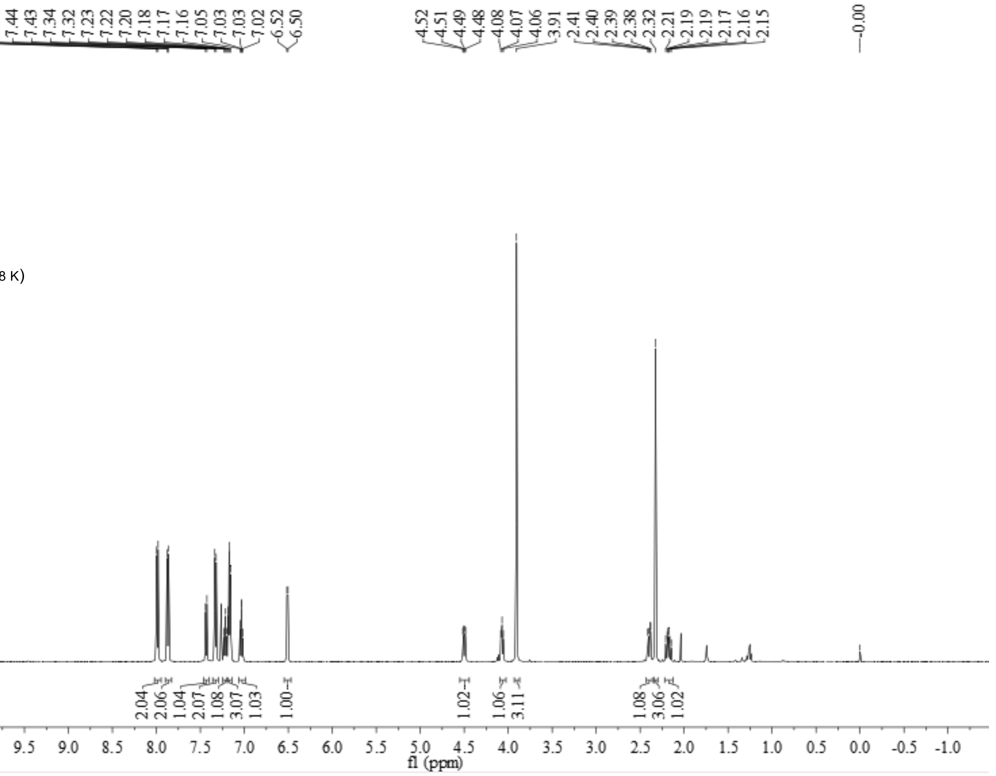

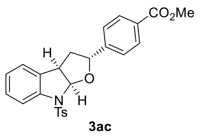

3ac

^{13}C NMR (126 MHz, $CDCl_3$, 298 K)

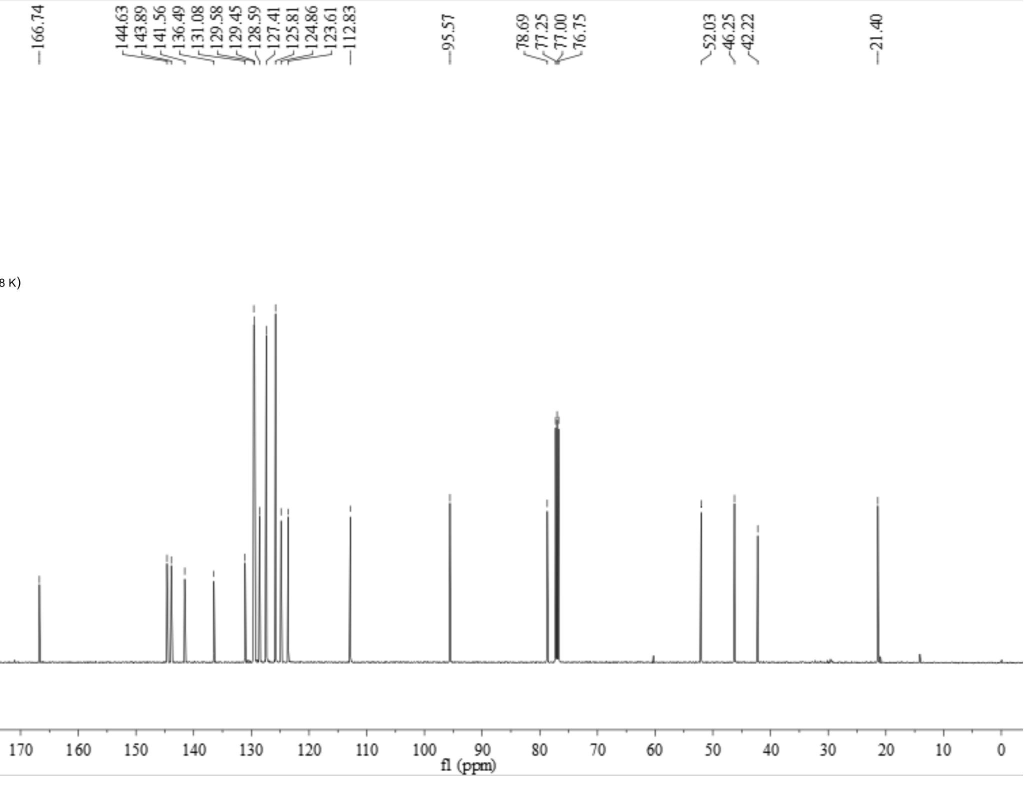

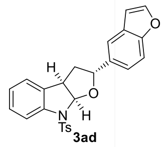

3ad

^{1}H NMR (500 MHz, $CDCl_3$, 298 K)

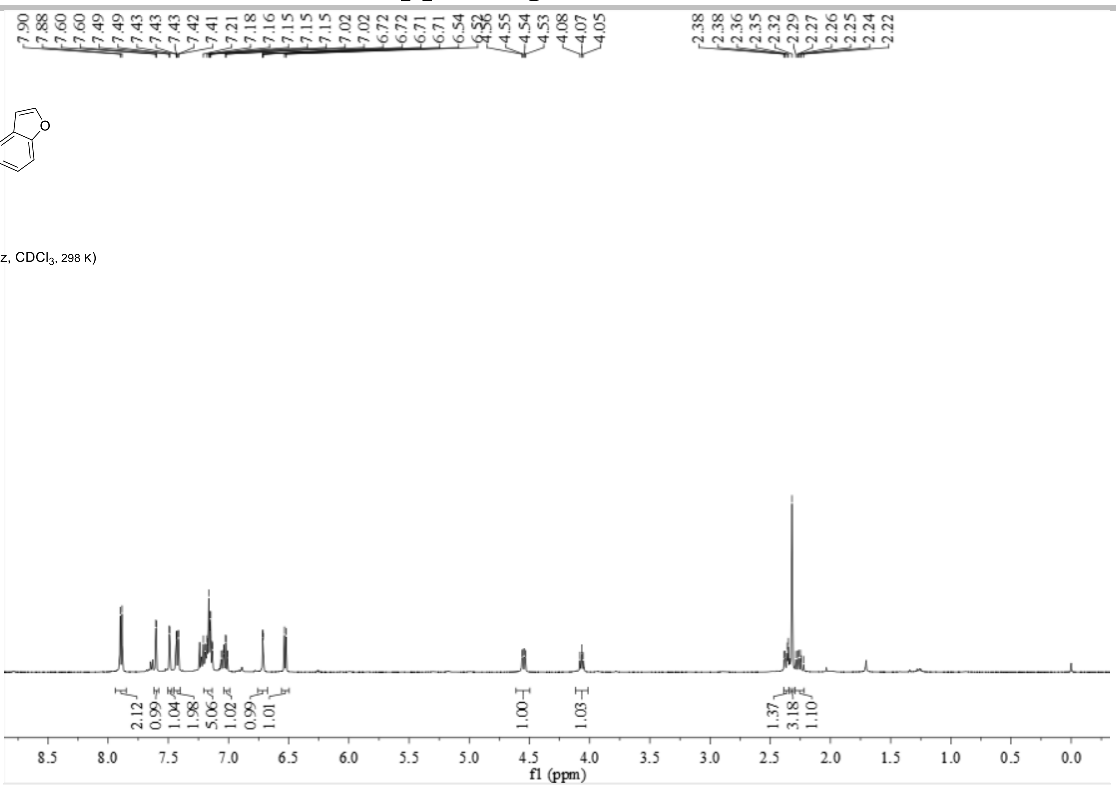

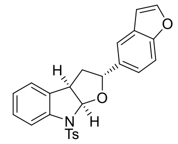

3ad

^{13}C NMR (126 MHz, $CDCl_3$, 298 K)

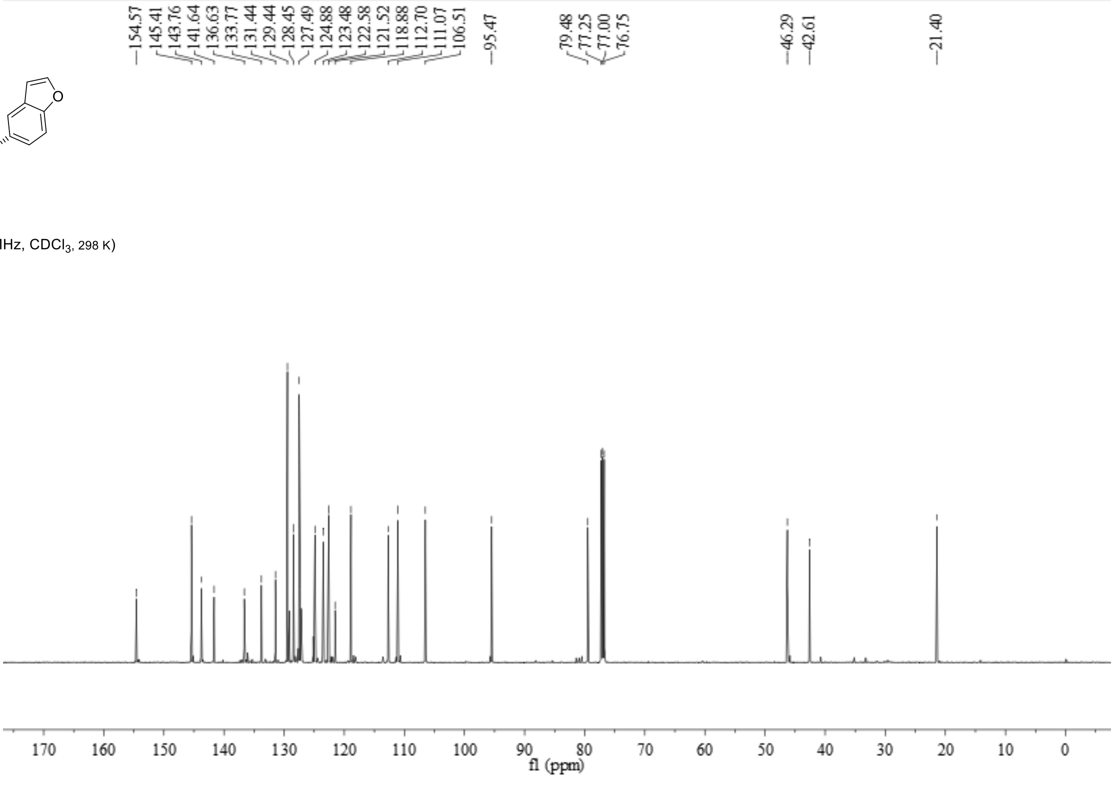

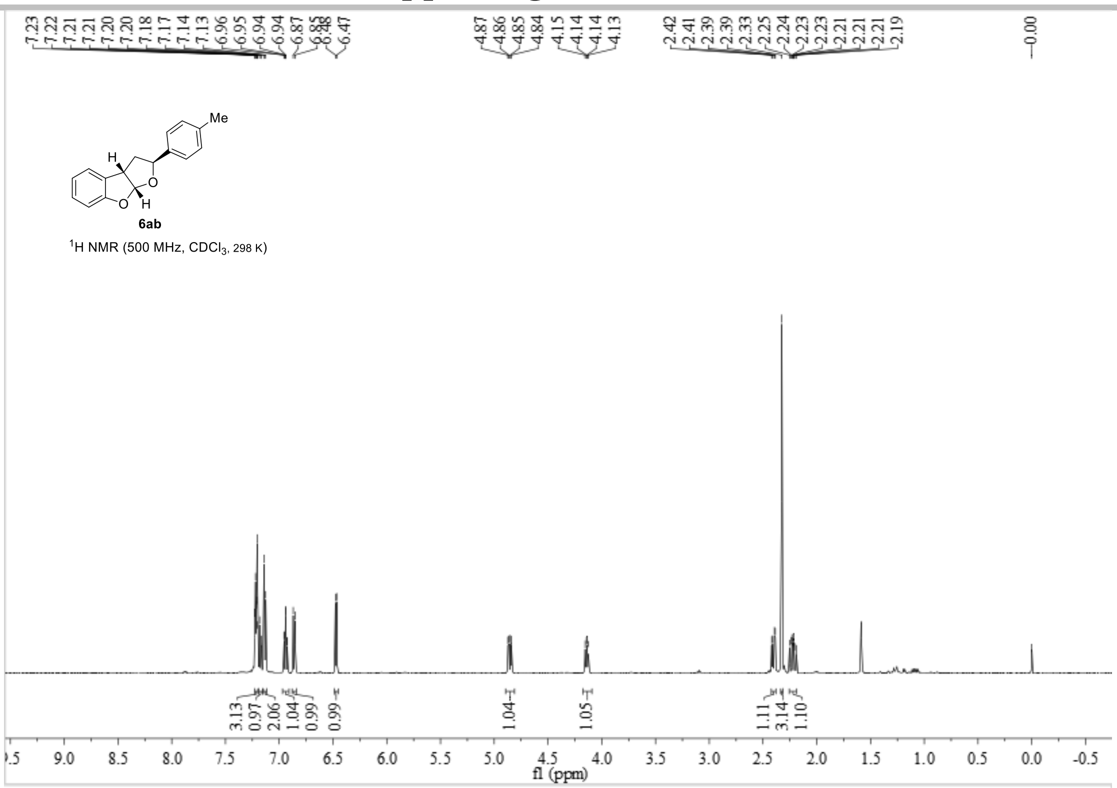
7.23 7.22 7.21 7.21 7.20 7.20 7.18 7.17 7.14 7.13 6.96 6.95 6.94 6.94 6.87 6.85 6.48 6.47
4.87 4.86 4.85 4.84 4.15 4.14 4.14 4.13
2.42 2.41 2.39 2.39 2.33 2.25 2.24 2.23 2.23 2.21 2.21 2.21 2.19
0.00
Me
H
O
O
H
6ab
1H NMR (500 MHz, CDCl3, 298 K)
3.13 0.97 2.06 1.04 0.99 0.99 1.04 1.05 1.11 3.14 1.10
f1 (ppm)

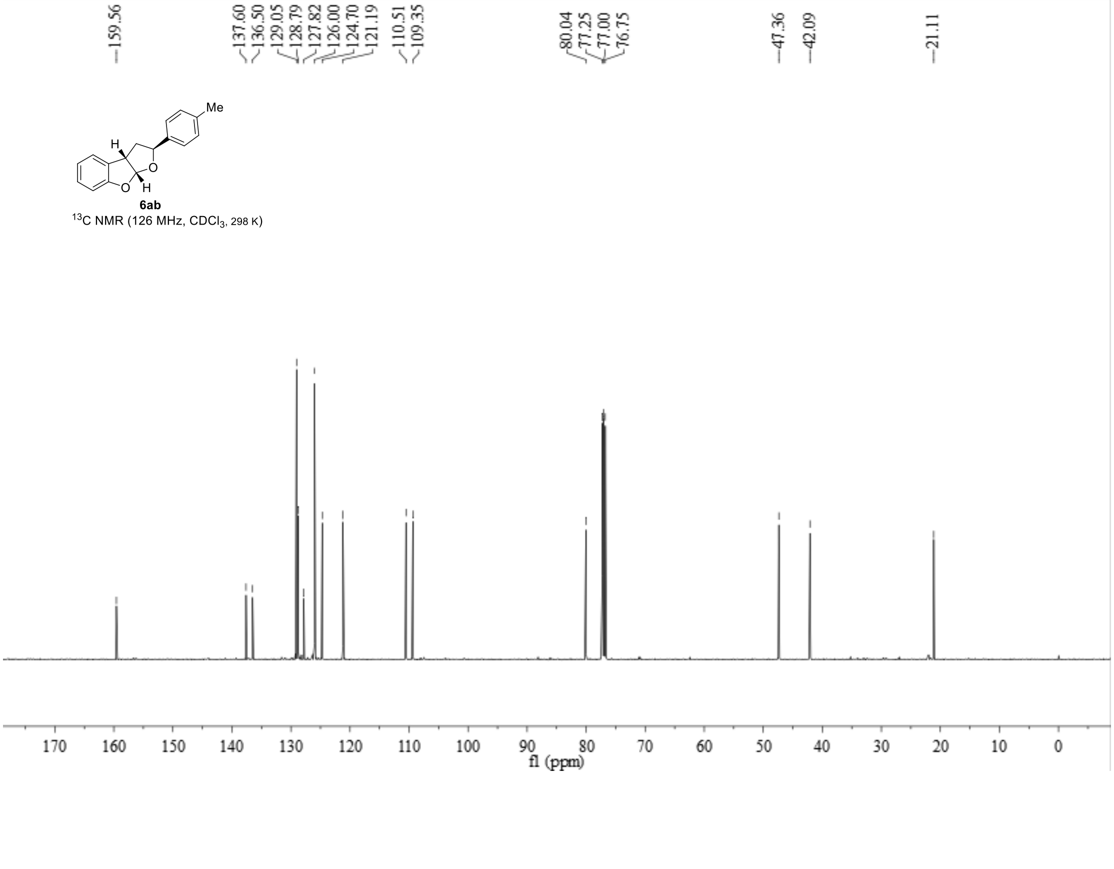
159.56
137.60 136.50 129.05 128.79 127.82 126.00 124.70 121.19 110.51 109.35
80.04 77.25 77.00 76.75
47.36 42.09 21.11
Me
H
O
O
H
6ab
13C NMR (126 MHz, CDCl3, 298 K)
f1 (ppm)

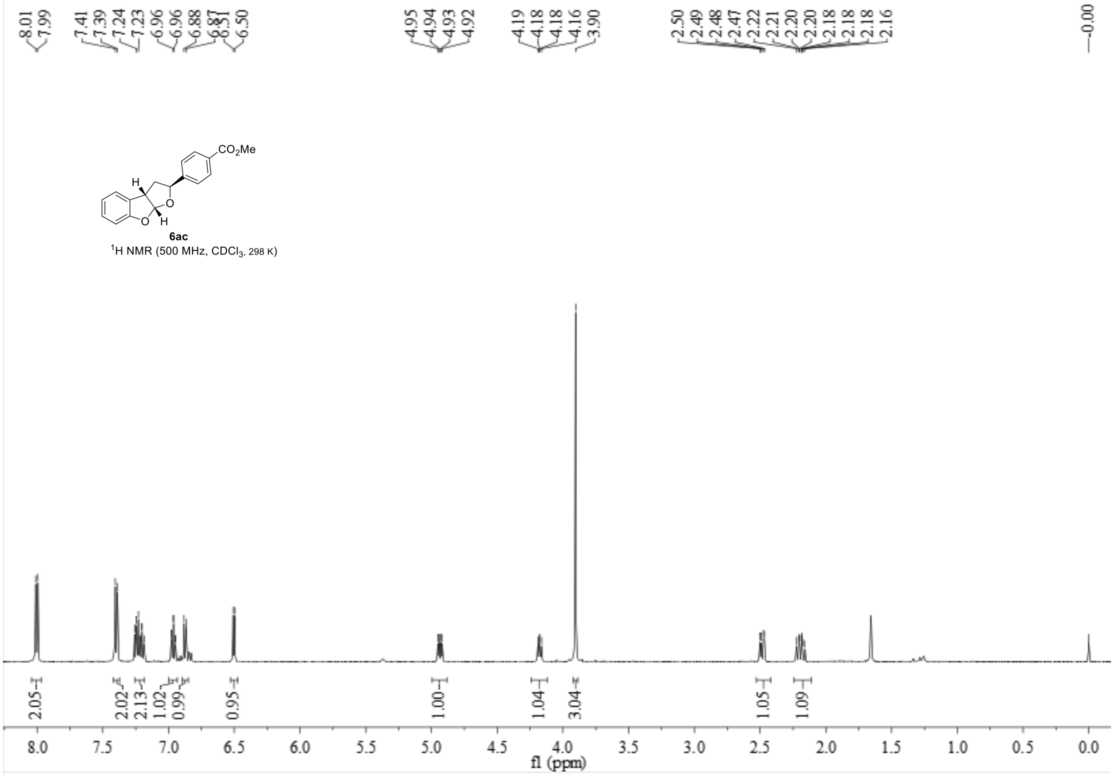
8.01 7.99 7.41 7.39 7.24 7.23 6.96 6.96 6.88 6.87 6.51 6.50 4.95 4.94 4.93 4.92 4.19 4.18 4.18 4.16 3.90 2.50 2.49 2.48 2.47 2.22 2.21 2.20 2.20 2.18 2.18 2.18 2.16 0.00
CO2Me
H
O
O
H
6ac
1H NMR (500 MHz, CDCl3, 298 K)
2.05 2.02 2.13 1.02 0.99 0.95 1.00 1.04 3.04 1.05 1.09
8.0 7.5 7.0 6.5 6.0 5.5 5.0 4.5 4.0 3.5 3.0 2.5 2.0 1.5 1.0 0.5 0.0
f1 (ppm)

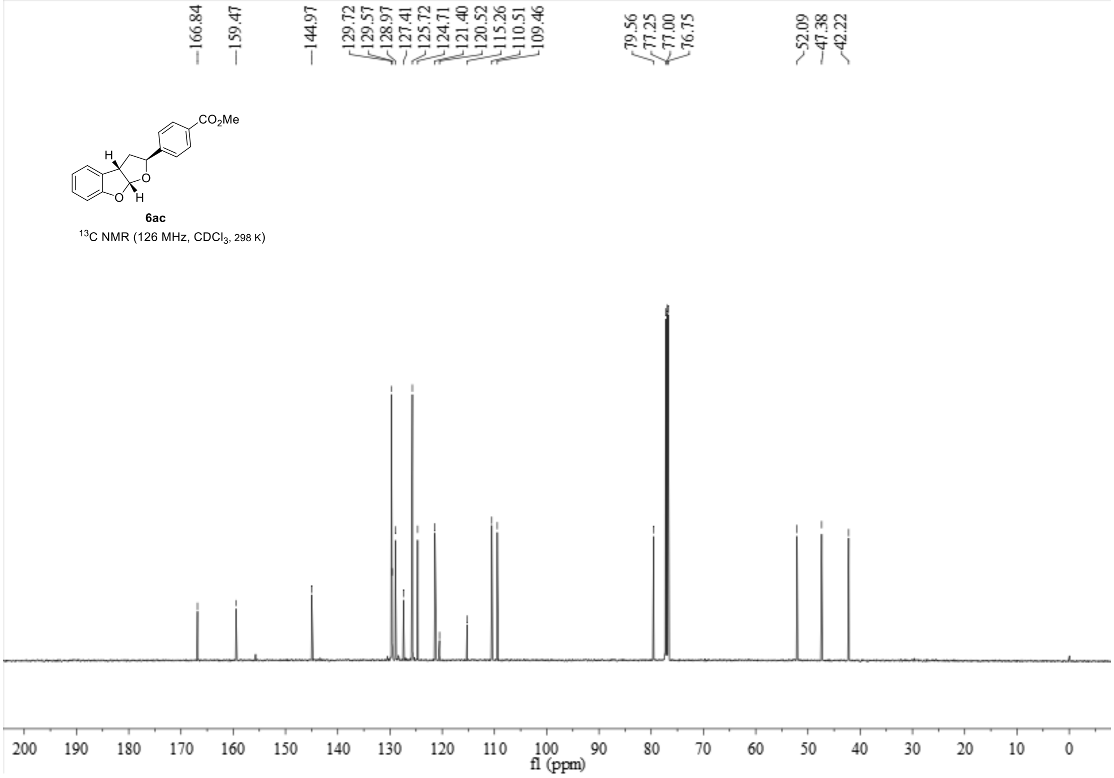
166.84 159.47 144.97 129.72 129.57 128.97 127.41 125.72 124.71 121.40 120.52 115.26 110.51 109.46 79.56 77.25 77.00 76.75 52.09 47.38 42.22
CO2Me
H
O
O
H
6ac
13C NMR (126 MHz, CDCl3, 298 K)
200 190 180 170 160 150 140 130 120 110 100 90 80 70 60 50 40 30 20 10 0
f1 (ppm)

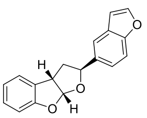

6ad

^{1}H NMR (500 MHz, $CDCl_3$, 298 K)

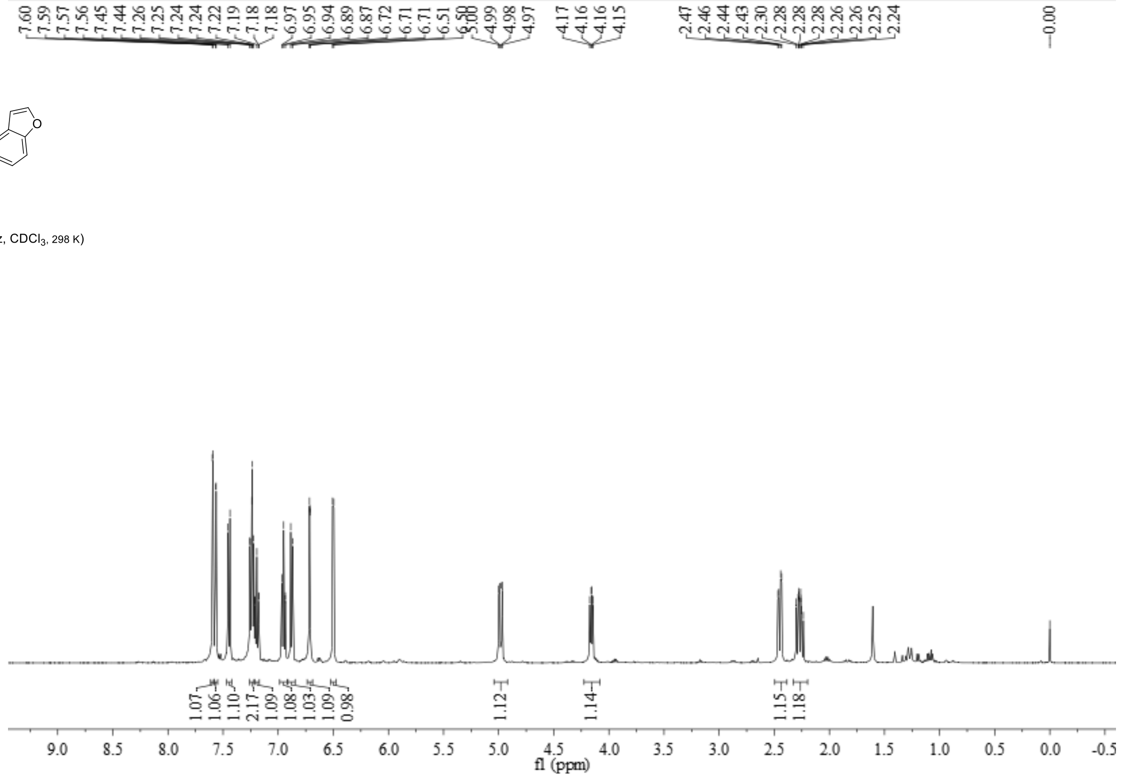

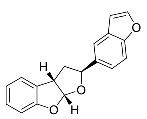

6ad

^{13}C NMR (126 MHz, $CDCl_3$, 298 K)

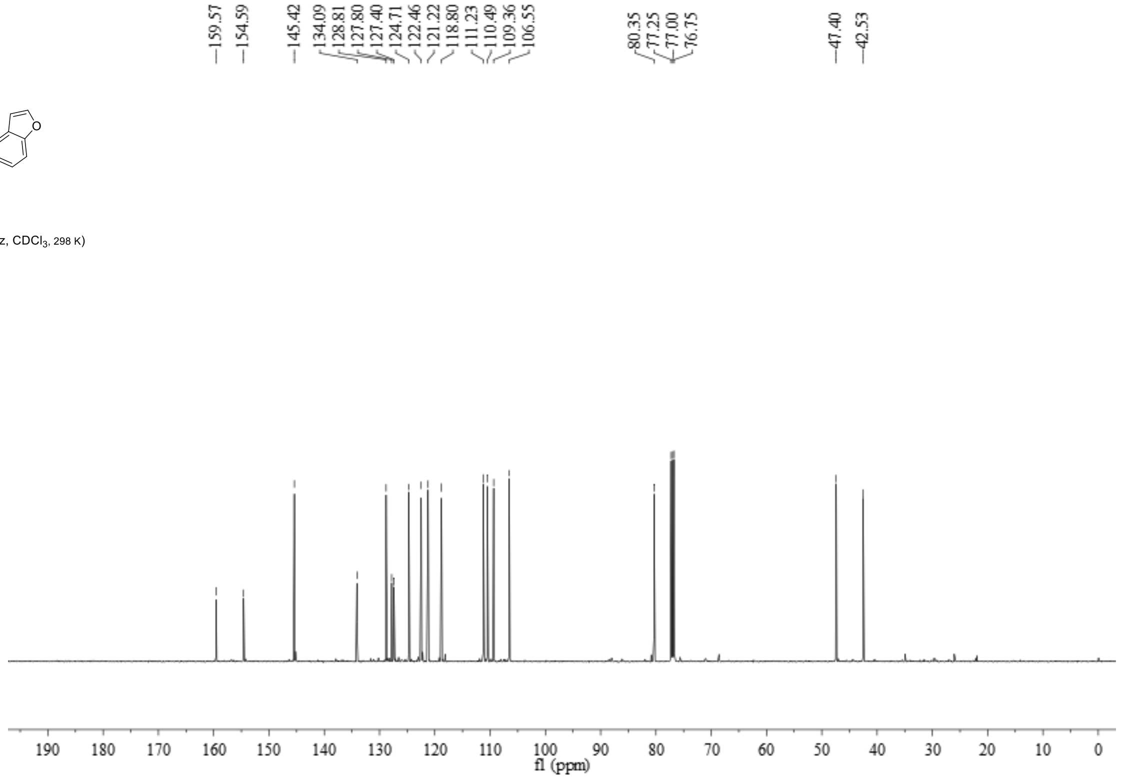

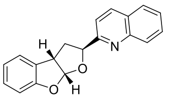

6ae

^{1}H NMR (500 MHz, $CDCl_3$, 298 K)

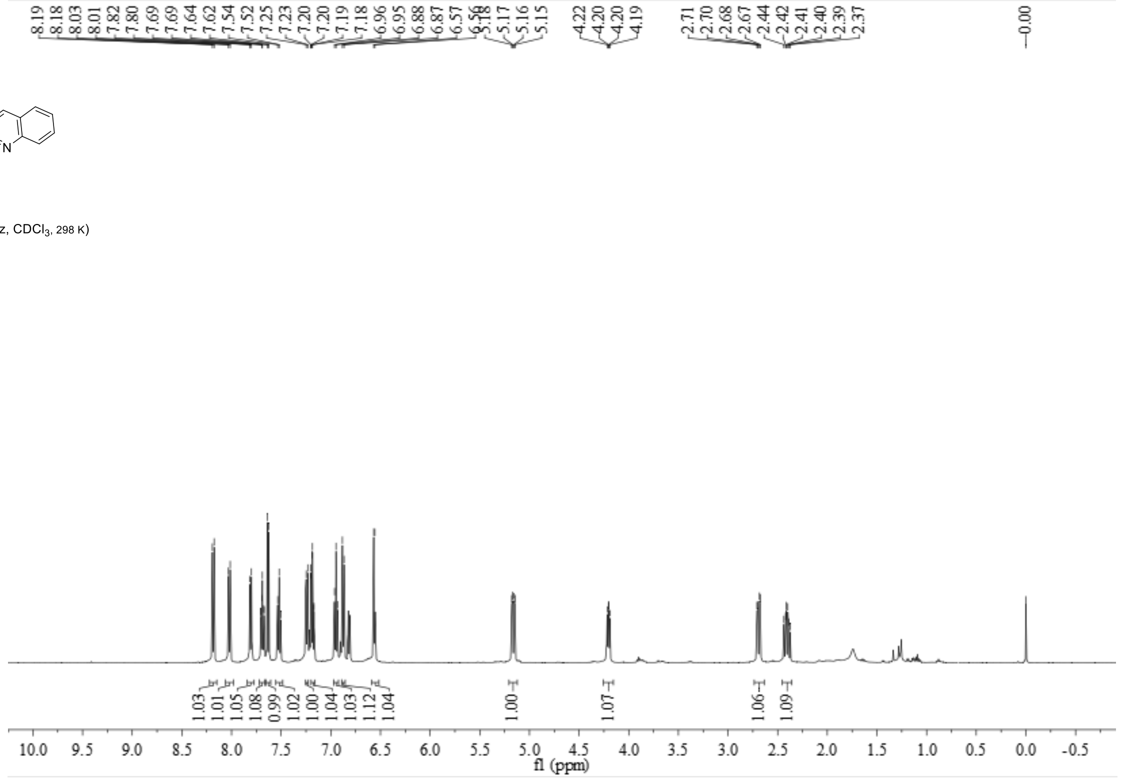

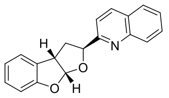

6ae

^{13}C NMR (126 MHz, $CDCl_3$, 298 K)

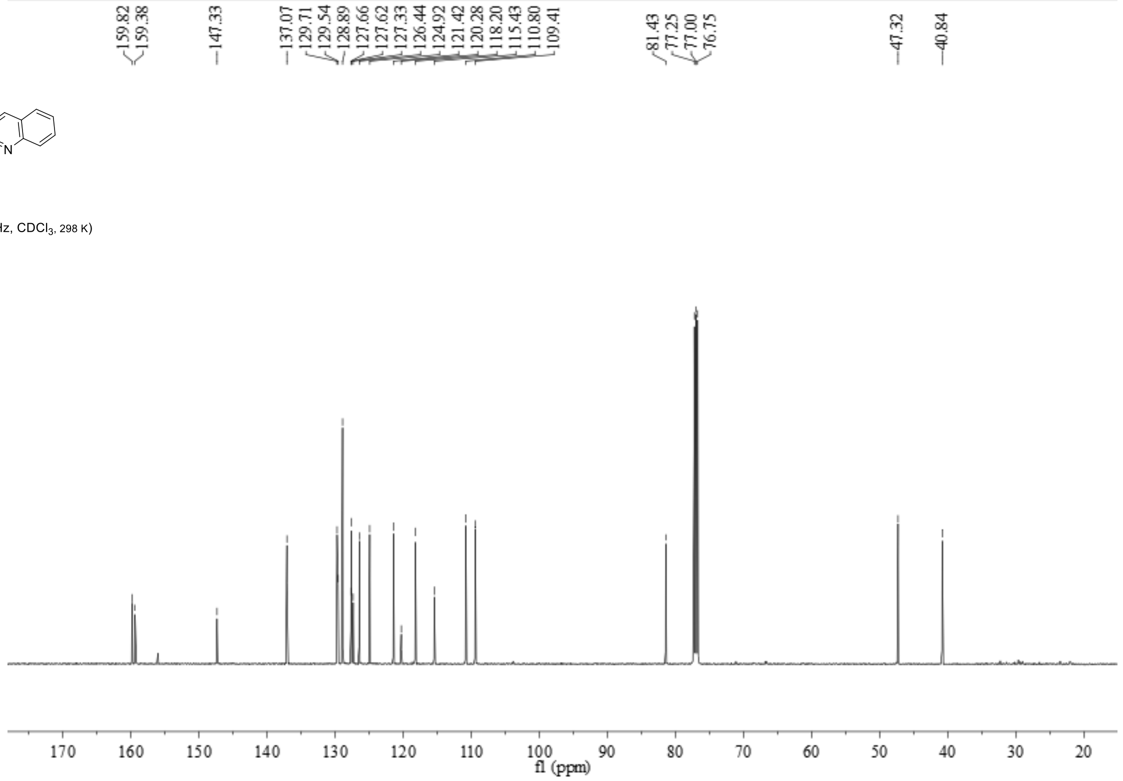

Supplement: Supplementary file 1 [file SC-011-D0SC01391A-s001.pdf]
